# Revisiting the Gold–Carbon Stretching Frequency: Spectroscopic and Computational Analysis of *N*‑Heterocyclic Carbene Monolayers on Nanostructured Gold Surfaces

**DOI:** 10.1021/acs.nanolett.6c01487

**Published:** 2026-08-03

**Authors:** Matteo Albino, Jonathan P. Wojciechowski, Molly M. Stevens

**Affiliations:** † Department of Materials, Department of Bioengineering, and Institute of Biomedical Engineering, 4615 Imperial College London, London, SW7 2AZ, United Kingdom; ‡ Department of Physiology, Anatomy and Genetics, Department of Engineering Science, Kavli Institute for Nanoscience Discovery, 6396 University of Oxford, Sherrington Rd, Oxford, OX1 3QU, United Kingdom

**Keywords:** *N*-Heterocyclic carbene, nanostructured gold, Hammett constant, SERS

## Abstract

*N*-Heterocyclic carbenes (NHCs) are notable ligands in surface and nanomaterial chemistry due to their improved stability compared with thiol self-assembled monolayers (SAMs). This stability originates from a strong carbon–gold bond; hence, insights into binding of NHCs on metal surfaces are valuable, with implications in many fields. In this work, 5-functionalized 1,3-diisopropyl benzimidazolium NHCs were deposited on nanostructured gold and analyzed using surface-enhanced Raman spectroscopy (SERS) to study the tuning of the carbon–gold stretching frequency (*ca*. 800 cm^–1^) based on 11 derivatives. Density functional theory (DFT) analysis demonstrated that the C–Au bond strength is not reflected in the SERS vibration, but the R-group does influence the ligand–Au interaction, with more electron-donating groups associated with increased bond strength (up to 4.63 kcal mol^–1^). Therefore, the C–Au stretching frequency from the SERS spectra is not a direct measure of the carbon–gold bond strength.

## Graphical abstract

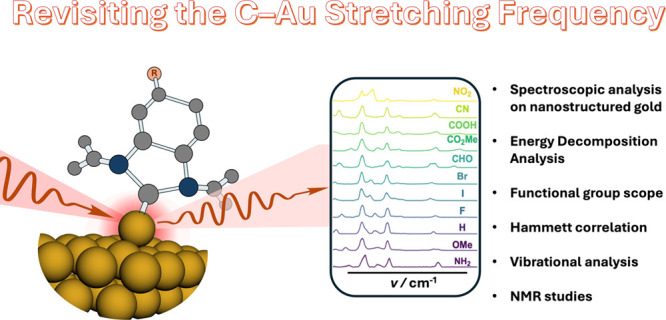

Since their initial report,^1–3^ *N*-heterocyclic carbene self-assembled monolayers (NHC-SAMs) have been demonstrated as promising ligands for the modification of precious metal surfaces when utilized for catalysis,^4,5^ molecular junctions,^6^ organic field-effect transistors,^7^ biosensors,^8^ and other applications.^9–13^ The impressive physical (*i.e*., extremely high desorption and functional temperatures)^6,14^ and chemical (*i.e*., resistance to acids, bases, organic solvents, and a wide range of reagents)^1,15–18^ properties of the monolayers have prompted significant efforts to understand the binding and electronic interactions of this class of ligands with metallic surfaces.^19–26^

NHC-SAMs have also demonstrated promising applications in nanotechnology.^27^ By modification of nanoparticles with NHCs, properties such as superior thermal and redox stability,^19,28^ the modulation of the electronic properties on gold surfaces,^7^ and improved colloidal stability in biological media have been achieved.^15,29^ Hence, NHC-modified nanomaterials have demonstrated promising applications in catalysis,^3,4,30–32^ optoelectronic materials,^28^ and sensing.^33^ Additionally, NHC-SAMs have the extraordinary capability to restructure gold surfaces, decreasing the overall roughness at the atomic scale once deposited,^34^ or to generate nanostructured architectures via postsynthetic modification of the monolayer,^2,35^ with such impressive precision that ‘writing’ at the nanoscale can also be achieved with these ligands.^13^

It was postulated that the superior physicochemical properties of the NHC-SAMs originated from a strong and robust C–Au bond,^1^ which has been thoroughly studied and experimentally observed at the molecular level.^36^ However, more recent developments have shown that the binding mode,^19^ wingtip modification *via N*,*N*′-alkylation,^19^ orientation of the ligand with respect to the surface,^19,22,23,37^ molecular ordering,^14^ and the type of carbene ligand^38,39^ all influence the stability of the SAM.

The high C–Au bond strength is often correlated with the strong σ-donating capabilities of carbene ligands, Figure 1a.^38^ However, evidence of π-backbonding was proposed in 2018 by DeJesus *et al.*, where compared to the molecular species, vibrations corresponding to the imidazolium rings were red-shifted upon binding to the surface, consistent with donation of electron density from the metal to the C–N σ* orbital.^18^ This was confirmed by Kuster *et al.*, where the Energy Decomposition Analysis–Natural Orbitals for Chemical Valence (EDA-NOCV) revealed that π-backbonding (*i*.*e*., π_1_ and π_2_ combined, Figure 1b and c) accounted for 13–15% of the C–Au bond strength.^25^ Hence, a holistic approach is required to analyze the electronics of NHC binding onto metal surfaces.

**Figure 1:**
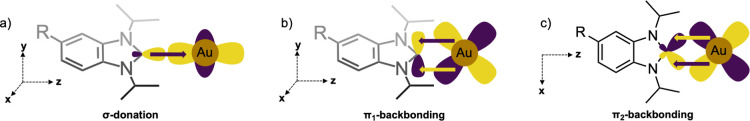
Schematic representation of main orbital interactions between a gold atom on a surface and an NHC ligand based on a 1,3-diisopropyl benzimidazolium, showing **a)** σ-donation, **b)** π_1_-backbonding, and **c)** π_2_-backbonding. Note, 5d orbital nodes not shown for clarity. Arrows indicate electron density donation.

Vibrational spectroscopy is widely utilized to study the interactions of ligands with metals, as changes in stretching frequency can give significant insight into the metal–ligand bonding in terms of bond strength and electronic interactions.^40–45^ Within the NHC-SAMs field, vibrational spectroscopy has also been widely utilized, but primarily to study the binding mode of the ligand with respect to the surface,^19,21,23–25,37,46^ and for process optimization.^18,23,47^ The former was pioneered by Camden and Jenkins where a combinatorial use of Surface-Enhanced Raman Spectroscopy (SERS) and Density Functional Theory (DFT) was utilized to deconvolute the wingtip-dependent orientation of NHC ligands with respect to the gold surface,^37^ to assign vibrations observed in the SERS spectra of NHC-SAMs,^46^ and to determine the C–Au stretching frequency.^24^

In our recent work, we utilized SERS to develop a reliable and reproducible solution-based methodology to deposit a range of NHCs on nanostructured gold surfaces,^47^ more specifically gold nanopillars.^48^ While expanding the scope of R-groups available, we noticed differences in the C–Au stretching frequency at *ca*. 800 cm^–1^ and were intrigued to determine such dependency. Hence, using a combinatorial analysis composed of spectroscopy (SERS and Nuclear Magnetic Resonance, NMR) and computational methods (DFT), we show that the origin of the C–Au stretching frequency shifts are dependent on strong vibrational coupling of the R-group with the carbene backbone, rather than a reflection of the strength of the C–Au bond.

Initially, 5-functionalized 1,3-diisopropyl benzimidazolium monolayers were deposited onto gold using our previously developed methodology and analyzed using SERS.^47^ The substituents chosen span almost the entire range of Hammett constants (σ_p_ ranges from −0.66 to 0.81)^49^ and were either synthetically accessible or were obtained using postsynthetic modifications of the monolayer (*i*.*e*., CO_2_Me hydrolysis to COOH^18^ or NO_2_ reduction to NH_2_).^16^ Figure 2a shows the SERS spectra obtained after depositing 5-functionalized 1,3-diisopropyl benzimidazolium monolayers on gold utilizing our methodology, with Figure 2b focusing on the region at *ca*. 800 cm^–1^, where the dominant vibration was found by Camden and Jenkins to correspond to a mode with a significant C–Au bond component.^24,46^ Compared to other characteristic vibrations of these SAMs (see Section S4), the peak at *ca*. 800 cm^–1^ shows significant shifts as a function of the R-group. Next, we plotted the stretching frequency versus the Hammett parameter, σ_p_, as a representative Hammett parameter (Figure 2c, *vide infra* for choice of constant), but no clear correlation was observed. No correlation was found either with any other Hammett parameter or vibration studied; see Section S4. However, the plot clearly shows that the R-group on the carbene backbone has the ability to tune the C–Au stretching frequency, with the bromide, iodide, and nitro derivatives showing a significant blue-shift relative to the unsubstituted benzimidazolium. Additionally, the measured shifts are well-simulated by DFT, Figure 2d, where the experimental and predicted ν_C–Au_ show excellent correlation (and generally with the whole SERS spectrum, Section S5), confirming that DFT can predict such shifts and is a suitable tool to study their origins.

**Figure 2:**
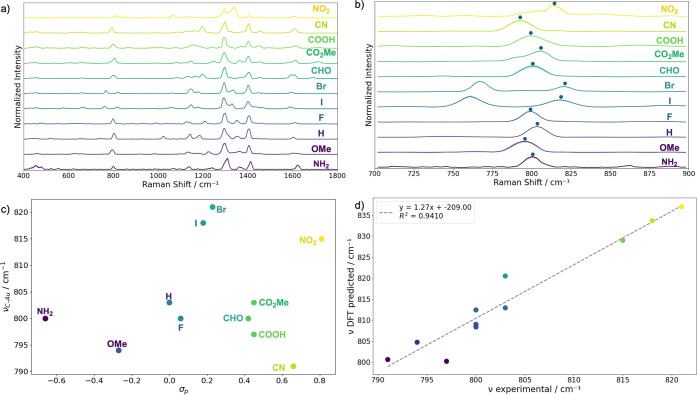
**a)** Stacked SERS spectra for 5-functionalized 1,3-diisopropyl benzimidazolium NHCs deposited on gold with varying R-groups and **b)** zoomed in between 700 and 900 cm^–1^ from panel a, with the maximum labeled in each spectrum (data shown as mean of *N* = 3, *n* = 5 spectra). The peaks at *ca.* 760 cm^–1^ for R = I and R = Br are different vibrations (see Section S5 for details). Reproduced from Albino *et al.* ^47^ Copyright 2025 American Chemical Society. **c)** Experimental C–Au stretching frequency versus Hammett parameter σ_p_ and **d)** experimental versus DFT-predicted C–Au stretching frequency at the BP86/genecp­(Au, I = Def2TVZP; C, H, N, O = Def2SVP)/D3BJ level of theory; see SI Section S3.1 for further details. All Hammett values for this work were obtained from the work of Taft and colleagues.^49^

To further understand the observed shift in C–Au stretching frequency, we characterized the electronic properties of these derivatives. The ^1^ *J*_C–H_ values obtained from NMR between the C-2 atom and proton of the cationic precursor have been found to correlate well with the σ-donating properties of the respective carbenes, with larger ^1^ *J*_C–H_ values implying a weaker donor.^50–52^ Meanwhile, a decreased LUMO energy as predicted by DFT suggests a stronger π-acceptor.^50^

We recorded proton-coupled ^13^C NMR spectra of the benzimidazolium mesylate precursors to obtain ^1^ *J*_C–H_ values (Figure 3a) and plotted them as a function of σ_p_ (Figure 3b; other Hammett parameters did not show as good of a correlation, Figures S35–S37). As expected, electron-donating substituents increase the σ-donating properties of the ligand. Meanwhile, DFT was employed to study the π-accepting properties, where the opposite trend was observed: electron-withdrawing substituents lower the energy of the LUMO, therefore increasing the π-accepting properties of the ligand (Figure 3c). Interestingly, compared to previous literature, we found an excellent correlation between the σ-donating properties and the energy of the carbene lone pair (Figure 3c).^50^ We postulate this is because the molecules studied all belong to the same class of ligand, while different frameworks were studied by Ganter and colleagues.^50^ It is also interesting to note that the LUMO energies span a wider range of values (*ca*. −1.9 eV), versus the carbene lone pair (*ca*. 0.7 eV).^50^ This is consistent with the π orbitals of the R-group being orthogonal to the carbene lone pair and therefore not modifying its energy significantly, but doing the opposite for the LUMO by overlapping with the π-system on the benzimidazole framework. This is clearly demonstrated in the depiction of the relevant molecular orbitals (Figure 3d-i). As such, a strong mesomeric interaction from the R-group requires utilization of σ_p–_ or σ_p+_;^49^ the former does in fact yield better linearity than σ_p_ or other constants (Figures S38–S41). The electronic characterization of the ligands is therefore consistent with previous reports in the literature.^53^

**Figure 3:**
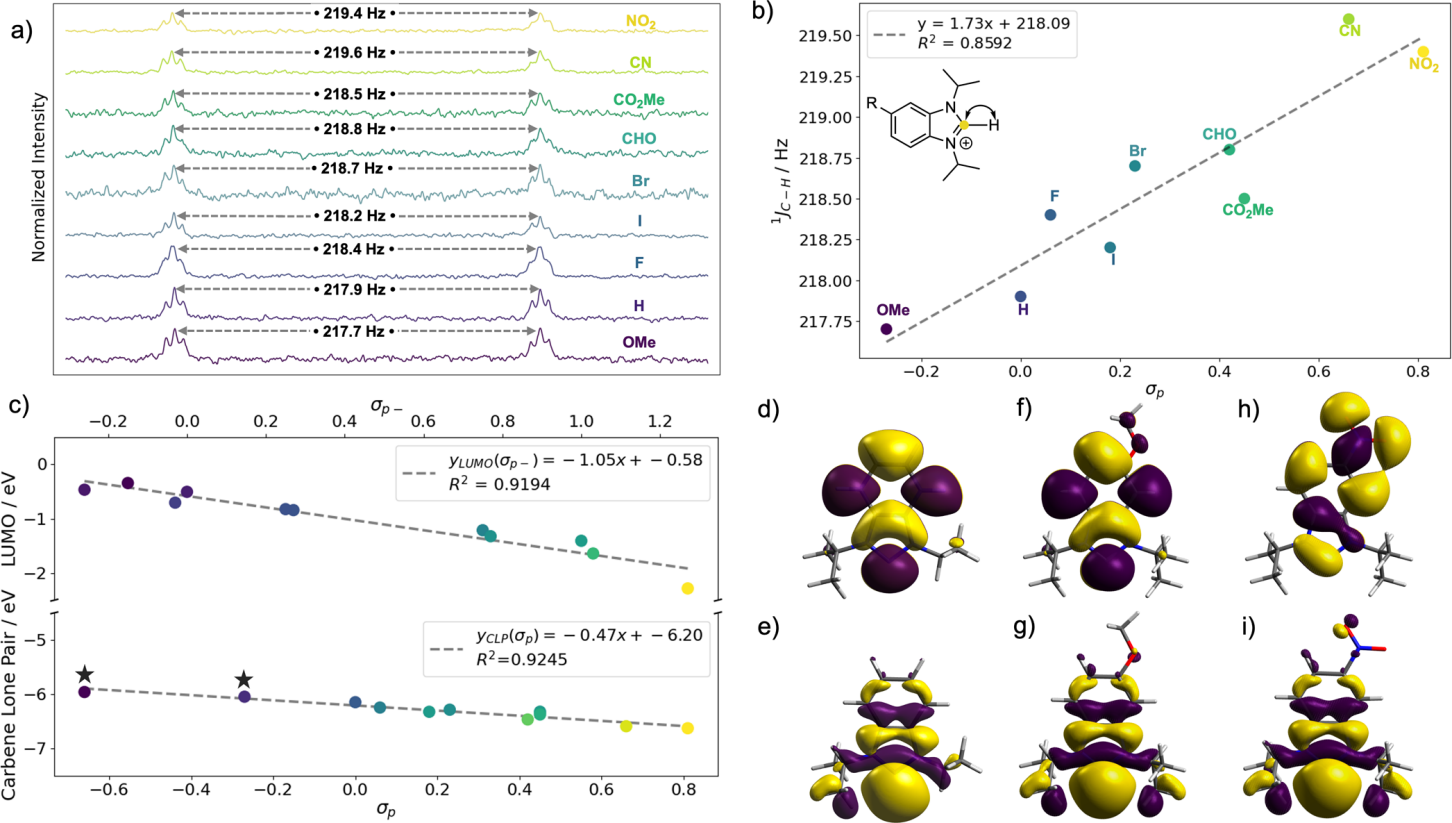
**a)** Zoomed ^1^H-coupled ^13^C NMR of the benzimidazolium precursors showing the ^1^ *J*_C–H_ value between the C-2 atom and proton (full spectra shown in Figure S34). Because of different δ­(^13^C), the signals were arbitrarily aligned. This does not influence the ^1^ *J*_C–H_ value calculated. **b)** ^1^ *J*_C–H_ plotted as a function of σ_p_. **c)** Carbene Lone Pair (CLP) and LUMO energy values for the carbene ligands as a function of σ_p_ and σ_p–_, respectively, at the PBE0/Def2TZVP/D3BJ level of theory. The data points labeled with a star (R = NH_2_ and OMe) indicate that the value presented are for the HOMO–1, which corresponds to the carbene lone pair. The HOMO was an orbital with π-symmetry (see SI, Figures S42 and S45). **d)**, **f)**, and **h)** Depiction of the LUMO and **e)**, **g)**, and **i)** of the carbene lone pair for R = H, OMe, and NO_2_, respectively, at the same level of theory. Hydrogen atoms are depicted in white, carbon in gray, nitrogen in blue, and oxygen in red; isovalue = 0.015 e bohr^–3^.

Hence, these plots seem to suggest the R-groups at the 5-position behave as *para* substituents and justify the choice of σ_p_ as the primary constant utilized in this work. Nevertheless, as shown schematically in Figure 1, the π-acidity of the ligand will be influenced by the energy of the LUMO, which is better described as a function of σ_p–_. Therefore, the latter will be used for studying the π_1_-accepting (see Figure 1b) properties of different derivatives.

Having shown that the R-group influences the electronic properties of carbenes, we sought to gain further insight into the binding strengths of different derivatives as a function of σ_p_, or σ_p–_ for π-acidity, by employing EDA-NOCV,^25^ where the total bond energy is separated into the electrostatic component, the exchange repulsion, the dispersion contributions, and the orbital energy. The latter can be deconvoluted further to look at specific interactions (σ-donation and π-backbonding, among others).^54,55^

Although the degree by which the gold adatom is ‘pulled out’ from the surface varies, especially between different wingtip modifications on the NHC ligands,^26^ the SERS spectra of 1,3-diisopropyl benzimidazolium-based monolayers are dominated by structures where the NHC binds to tips and adatoms on the surface, as proven by Camden, Jenkins, and co-workers.^21^ Hence, to computationally study the binding of this specific ligand to gold surfaces, we utilized a previously published cluster where the NHC is bound to an adatom and the isopropyl wingtips are facing down. While this is a finite cluster, it provides good agreement with experimental results.^21,37,46^

The computational results, summarized in Figure 4a, are in good agreement with the previous characterization. Notably, more electron-donating groups increase the strength of the C–Au bond, Figure 4b. This is due to both a significant increase in the strength of the electrostatic interaction (Δ*E* = −7.61 kcal mol^–1^ across the series, Figure S61) and, to a lesser extent, the orbital interaction (Δ*E* = −1.34 kcal mol^–1^ across the series, Figure 4c). We find σ-donation is the predominant interaction that accounts for *ca*. half of the total orbital energy. The interaction energy decreases as the σ_p_ decreases (Figure 4d and h), consistent with the trend in ^1^ *J*_C–H_ values (*vide supra*). Electron-withdrawing groups increase both the π- and σ-accepting properties of the ligand (Figure 4e–g and i–k), but in isolation, these account for a significantly smaller proportion of the orbital energy, and therefore the bond strength. This also explains the minimal variability in the vibrations at *ca*. 1300 and 1400 cm^–1^ in the SERS spectra of the different ligands deposited on the nanostructured gold (Figures S6–S13). Although these vibrations have contributions from the C–N bonds within the benzimidazolium ring, and therefore different levels of π_2_-backbonding should modulate the bond strength by varying the level of electron density in the σ* orbital, the values only vary by 1.08 kcal mol^–1^ across the series of σ_p_ values; hence the change in bond strength should not be significant. Other significant interactions (electrostatic interaction, exchange repulsion, dispersion contributions, and other orbital interactions) are summarized and plotted as a function of σ_p_ in Section S8.

**Figure 4:**
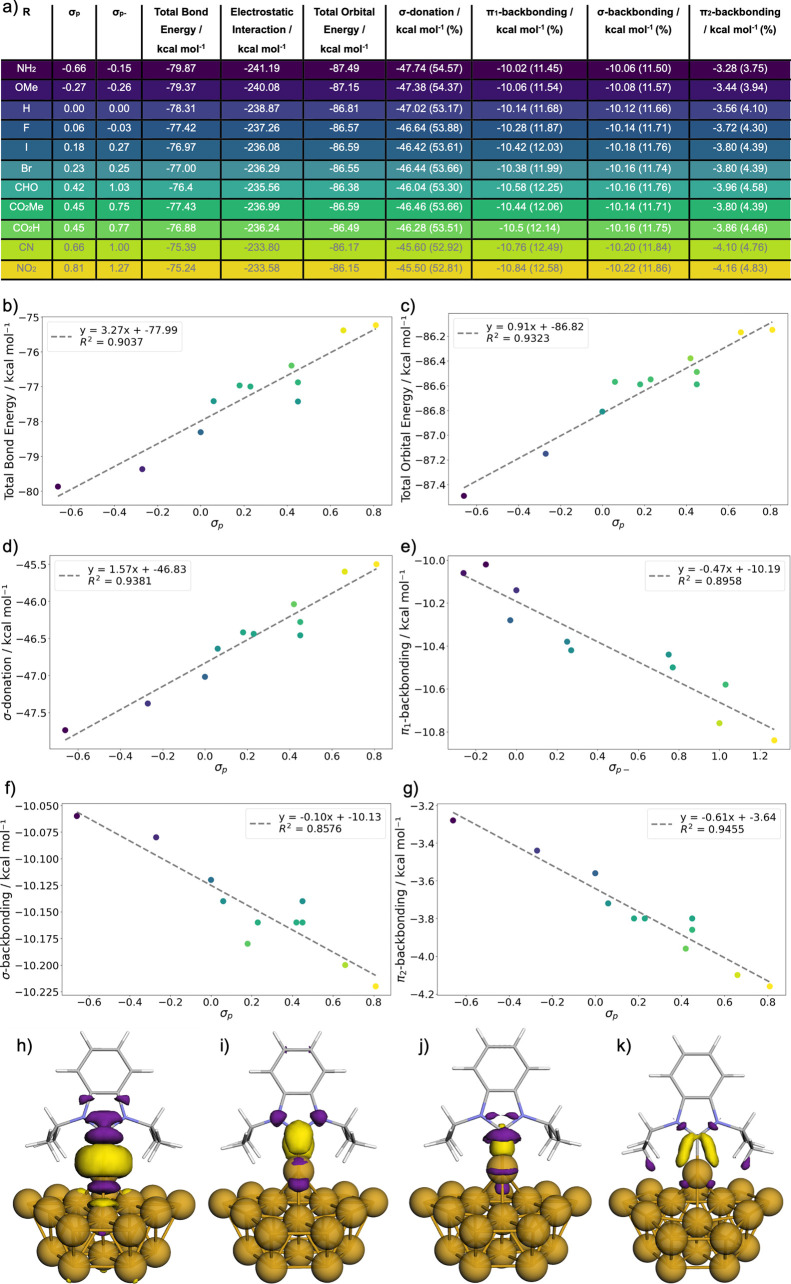
**a)** Table summarizing the EDA-NOCV results in terms of total bond energy, electrostatic interaction, total orbital energy, σ-donation, π_1_-backbonding, σ-backbonding, and π_2_-backbonding. The values in parentheses are the % contribution of the specific interaction with respect to the total orbital energy. **b)**, **c)**, **d)**, **e)**, **f)**, **g)** Values of total bond energy, total orbital energy, σ-donation, σ-backbonding, and π_2_-backbonding as a function of σ_p_ and π_1_-backbonding as a function of σ_p–_. **h)**, **i)**, **j)**, **k)** Plot of the deformation densities between the ligand (R = H) and the metal surface for the σ-donation, π_1_-backbonding, σ-backbonding, and π_2_-backbonding. Isovalue = 0.0005 e bohr^–3^, yellow signifies gain of electron density, while purple signifies loss. See SI, Section S3.3 for details on the level of theory.

Importantly, the linearity obtained between bond strength and σ_p_ values is not reflected within the tuning of the stretching frequency (Figures 4b and 2c, respectively). Hence, we conclude that the C–Au bond strength is not related to this vibration, and other factors must be responsible for the shift.

We therefore reconsidered the computational vibrational analysis. The stretching frequency is proportional to the square root of the force constant over the reduced mass. If the C–Au vibration is coupled to other vibrations, especially when related to the R-group, these factors are expected to change. Hence, we considered the vibration holistically and analyzed all these factors, which are summarized in Figure 5a.

**Figure 5:**
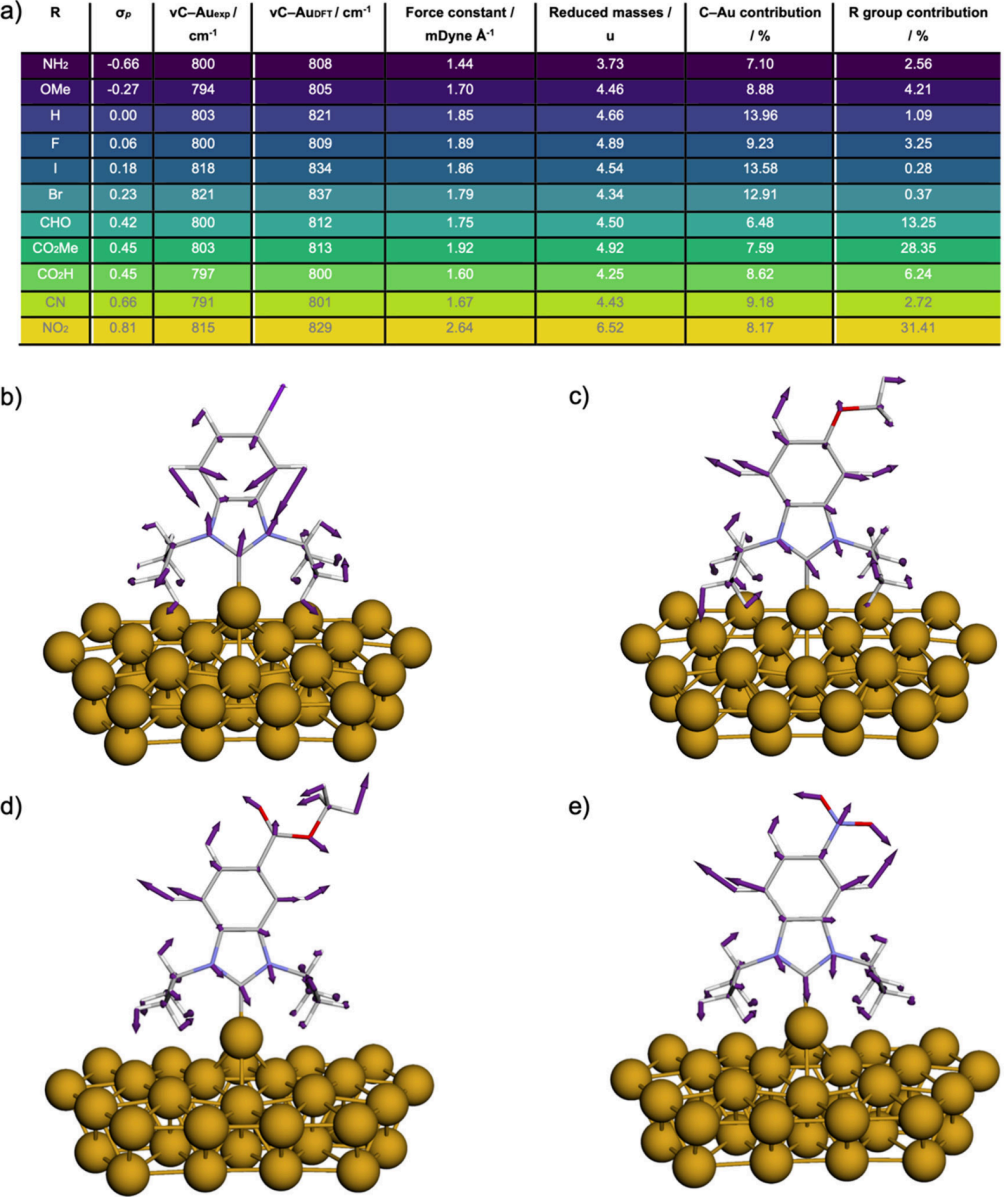
**a)** Table comparing the experimental and DFT-computed C–Au stretching frequency and the force constant, reduced masses, C–Au percentage contribution, and R-group percentage contribution for the different derivatives. **b)**, **c)**, **d)**, **e)** The C–Au vibration for different R-groups (R = I, OMe, CO_2_Me, NO_2_) clearly displaying an increased contribution from the R-group. The arrows represent normalized atomic displacement vectors for each atom.

Clearly, the R-group contribution changes drastically between the derivatives. The bromide and iodide (Figure 5b) derivatives demonstrate almost negligible contributions from these atoms, while the NO_2_-scissoring accounts for almost a third of the vibrational motion (Figure 5e), with other R-groups participating somewhere in-between these derivatives (Figure 5c and d). As a result, the force constant, reduced masses, and more importantly the C–Au contribution change drastically, therefore explaining why there is little correlation between the stretching frequency herein studied and the ligand binding strength to the surface.

NHC-SAMs are often utilized because of their high Au–C bond strengths.^1^ Previous reports have attributed this high bond strength to a combination of strong σ-donating capabilities of carbene ligands and π-backbonding. We observed a trend in the SERS spectra of the C–Au stretching frequency (*ca*. 800 cm^–1^) of 5-functionalized 1,3-diisopropyl benzimidazolium monolayers on gold based on their substituents and explored the origin of this shift in this study. This factor was studied computationally to determine whether it could be utilized as an experimental value to be correlated to the C–Au bond strength, which has been computed using EDA-NOCV. However, due to variable vibrational coupling between said stretching frequency and those correlated to the R-group, the reduced masses and force constants are altered, therefore making it inaccurate to use this stretching frequency as a measure of the C–Au bond strength: as such, other experimental parameters should be investigated. Nevertheless, we show that altering the 5-substituent is an efficient method of improving the C–Au bond strength, highlighting that the design of highly stable NHC-SAMs should consider the properties of the specific ligand, while maintaining focus toward the binding mode,^19^ wingtip modification *via N*,*N*′-alkylation,^19^ orientation of the ligand with respect to the surface,^19,22,23,37^ molecular ordering,^14^ and the type of carbene ligand.^38,39^

## Supplementary Material



## Data Availability

Computational inputs, xyz and output files, and custom data processing codes are available online at DOI: 10.5281/zenodo.20933742.

## Notes

General methods, full experimental and computational details, characterization data for all novel compounds, full computed SERS spectra, additional Hammett plots and DFT coordinates (PDF)
