# Supplementary material for: Revisiting the Gold–Carbon Stretching Frequency: Spectroscopic and Computational Analysis of N‑Heterocyclic Carbene Monolayers on Nanostructured Gold Surfaces
Source: Nano Lett. 2026 Aug 3;26(31):10115–22. doi: 10.1021/acs.nanolett.6c01487 (PMC13475047; doi:10.1021/acs.nanolett.6c01487)
Supplement: Supplementary file 1 [file nl6c01487_si_001.pdf]

SUPPORTING INFORMATION

Revisiting the Gold-Carbon Stretching Frequency: Spectroscopic and Computational Analysis of *N*–Heterocyclic Carbene Monolayers on Nanostructured Gold Surfaces

Matteo Albino,[1,2] Jonathan. P. Wojciechowski,[2]* and Molly. M. Stevens.[1,2]*

[1] Department of Materials, Department of Bioengineering, and Institute of Biomedical Engineering, Imperial College London, London, SW7 2AZ, United Kingdom
[2] Department of Physiology, Anatomy and Genetics, Department of Engineering Science, Kavli Institute for Nanoscience Discovery, University of Oxford, Sherrington Rd, Oxford, OX1 3QU United Kingdom

Number of Pages: 57; Number of Figures: 69; Number of Tables: 4.

Table of Contents

S1 General Methods 2
S2 Synthetic Methodologies 4
S3 Computational Details 5
S3.1 Generation of Computed SERS spectra 5
S3.2 Frontier Molecular Orbital Analysis of Ligands 6
S3.3 Energy Decomposition Analysis Natural Orbitals for Chemical Valence Analysis 6
S4 Correlations Between σ_p and Characteristic NHC–SAMs vibrations 7
S5 Computed SERS Spectra 14
S6 Proton Coupled ^{13}C Spectra 24
S7 Frontier Molecular Orbitals Analysis 27
S8 EDA-NOCV Analysis 39
S9 NMR Spectra 42
S10 DFT Coordinates 45
S9.1 DFT Coordinates for the Computed SERS Spectra and NOCV 45
S9.1 DFT Coordinates for Free Ligands Utilised for FMOs analysis 51
S11 References 56

S1 General Methods

Solvents and Reagents: All reagents and solvents were purchased from chemical suppliers including Fisher Scientific, Fluorochem, Merck, Sigma-Aldrich and TCI, and were used without any additional purification. 5 Å molecular sieves were purchased from Thermo Fisher Scientific and Fisher Scientific.

Nuclear Magnetic Resonance (NMR): NMR spectra were recorded on a 500 MHz spectrometer equipped with an Oxford Instruments Company magnet. Chemical shifts in ppm were calibrated from proton chemical shifts of the solvents. ^{1}H spectra were recorded at 500 MHz, and ^{13}C spectra recorded at 126 MHz. Coupling constants are reported in Hz. All spectra were processed using MestReNova. All spectra were recorded at room temperature (298 K).

Mass Spectrometry: Electrospray ionisation (ESI) was performed on a Thermo Exactive High-Resolution Orbitrap FTMS (University of Oxford). Flow injection analysis was performed on an ACQUITY I-Class PLUS UPLC System (Waters, Milford, MA, USA) coupled to an ACQUITY RDa mass spectrometer (Waters, Milford, MA, USA) equipped with an ESI probe, in positive ion mode (University of Oxford). Mass to charge ratios (m/z) are reported in Daltons. Where complex isotope patterns were observed, the most abundant ion is reported. High resolution mass spectra (HRMS) are reported with less than 5 ppm error.

Infrared Spectroscopy: Solid state or neat spectra were recorded on a Bruker Alpha II spectrometer. Absorption maxima (ν_{max}) are reported in wavenumbers (cm^{-1}) to the nearest whole number. Background was measured with 4 scans and a resolution of 2 cm^{-1}. 64 scans with a resolution of 0.5 cm^{-1} were used for each measurement between 400 and 4000 cm^{-1}.

Surface Enhanced Raman Spectroscopy (SERS)–substrates: All SERS-substrates were purchased from Silmeco ApS.

SERS-measurements: All spectra were acquired using a custom Raman microspectroscopy platform designed for high-throughput analysis known as 'B-Raman'.[1,2] Continuous-wave excitation at 785 nm was supplied by a single-mode diode laser (BRM-785-0.55-100-0.22-SMA, B&W Tek) and attenuated to 5 mW at the sample plane, as verified with a commercial power meter (PM121D, Thorlabs). The excitation line was spectrally cleaned with a 3 nm full width at half maximum clean-up filter (785 nm MaxLine®, LL01-785-

12.5, Semrock). Back-scattered photons were collected in an epi-illumination (backscattering) geometry through a 10×, 0.25 NA objective (N PLAN 10×/0.25, Leica). The excitation light was rejected by an ultrasteep long-pass dichroic (RazorEdge® 785 nm, LP02-785RU-25, Semrock) followed by a 785 nm notch filter (33 nm FWHM, NF785-33, Thorlabs). The spectral data was captured with a commercial high-performance Raman spectrometer (EAGLE Raman-S - Ibsen), which features a deeply cooled Andor's iVac 316 camera. The raw spectra were acquired over the spectral wavenumber range of 142.0 to 3684.8 cm^{-1}. For each condition, unless stated in the figure caption, 5 measurements on 3 separate SERS-substrates were recorded with 0.1 s integration time and 3 accumulations.

Data preprocessing: Raw measurements were pre-processed to account for spectral artefacts, such as cosmic spikes, baseline variations and experimental noise. The preprocessing involved:

(i) spectral cropping to the range 400–1800 cm^{-1} or 400–3000 cm^{-1};

(ii) cosmic spikes removal using the modified z-score algorithm presented by Whittaker *et al.*[3]

(iii) denoising using a second-order Whittaker smoother with $\lambda=1$,[4]

(iv) baseline correction using a second-order Improved Asymmetrically Reweighted Penalized Least Squares (IARPLS) algorithm with a smoothness factor $\lambda = 10$.[5] All preprocessing steps were implemented in Python using a combination of custom in-house scripts and open-source packages, including RamanSPy[6] and pybaselines[7]

Once the data was processed, the 15 measurements were averaged and plotted using custom in-house python code. The standard error of the mean was calculated and added to the plot as the ± range of the mean.

Plasma Cleaning: All SERS-substrates and spinners were cleaned using oxygen plasma before functionalisation using a Leybold CTR 100N electronic Diener machine. The chamber was first pumped down to 0.2 mbar and then backfilled with medical grade 95% O_2, 5% CO_2 gas mixture up to 0.3 mbar. The plasma was then activated at 400 watts for 30 seconds. Before inserting the spinners and the chips, the plasma cleaner chamber was cleaned for 5 minutes using the same procedure.

General procedure for the deposition of mesylates salts: We utilised a previously published procedure reported by our group.[8] The corresponding benzimidazolium and tetraethylammonium

bicarbonate (TEAB) (5 eq. for all benzimidazoliums, except for R = NO_2 where 2 eq. of TEAB were used) were added to a 28 mL glass vial. The solids were then suspended in THF to yield a 1 mM solution of benzimidazolium. 5 Å molecular sieves were activated by heating to 200 ºC under vacuum for 20 minutes, cooled to room temperature, added, followed by the cleaned SERS–substrates immobilised into the spinners, and the vial sealed. The mixture was stirred at 120 rpm at 55 ºC for 2 hours. The substrates were then rinsed with excess water (3 times) and methanol (3 times), and dried under a stream of dinitrogen. NOTE: the sieves are cream-colored in appearance, if over-heated they turn blue and the functionalisation fails. The amino and carboxylic acid monolayers have been synthesised using post-deposition modification of monolayers by the respective reduction of the nitro and hydrolysis of the carboxymethyl ester moieties.[8–10]

S2 Synthetic Methodologies

All 1,3-diisopropyl benzimidazolium mesylates precursors were synthesised as previously reported.[8] All derivatives have been fully characterised previously,[8] except for the iodide derivative for which characterisation data is reported below. **SAFETY NOTE:** significant pressure build up was observed during the synthesis of all derivatives, regardless of base, solvent and alkylating agent. We believe the production of CO_2 and propene from the competing elimination reaction could be responsible for this observation. Nevertheless, under reflux conditions we saw a significant decrease in the reaction rate. We therefore encourage caution and the use of blast shields for reactions under pressure before repeating the procedure reported.

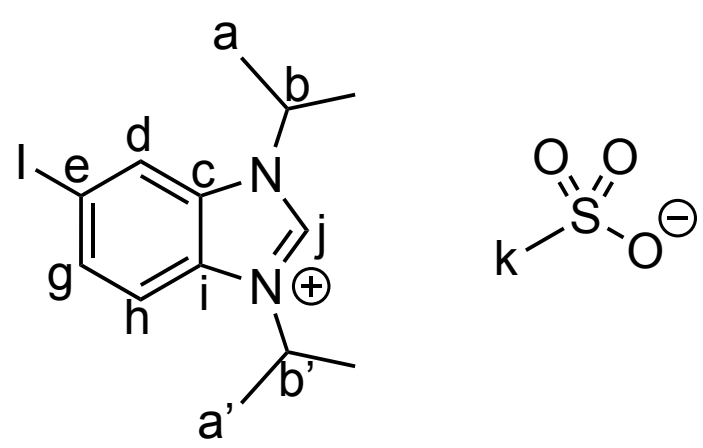

5-iodo-1,3-diisopropyl-1*H*-benzo[*d*]imidazol-3-ium methanesulfonate: 783 mg (56%), obtained from 800 mg (3.28 mmol) of starting benzimidazole as a colourless powder.

^{1}H NMR (500 MHz, Chloroform-*d*) δ 10.65 (s, 1H, H_j), 8.10 (d, J = 1.7 Hz, 1H, H_d), 7.89 (dd, J = 8.8, 1.5 Hz, 1H, H_g), 7.54 (d, J = 8.7 Hz, 2H, H_h), 5.08 (apparent dh, J = 27.3, 6.8 Hz, 2H, H_b and $H_{b'}$), 2.79 (s, 3H, H_k), 1.80 (apparent dd, J = 6.8, 3.5 Hz, 12H, H_a and $H_{a'}$).

^{13}C NMR (126 MHz, Chloroform-*d*) δ 141.9 (C_j), 135.8 (C_g), 132.4 (C_i), 130.6 (C_c), 122.9 (C_d), 115.5 (C_h), 90.6 (C_e), 53.0 (C_b or $C_{b'}$), 52.9 0 (C_b or $C_{b'}$), 39.7 (C_k), 22.0 (C_a or $C_{a'}$), 21.9 (C_a or $C_{a'}$).

FTIR (ATR) / cm^{-1}: 3469, 3418, 3115, 3046, 2981, 2933, 1641, 1550, 1468, 1454, 1430, 1378, 1322, 1283, 1248, 1215, 1175, 1144, 1113, 1037, 980, 894, 815, 765, 639, 564, 547, 503, 426.
HRMS (ESI+) m/z: Calculated mass for $C_{13}H_{18}IN_2^+$: 329.0509; measured: 329.0493.

S3 Computational Details

S3.1 Generation of Computed SERS spectra

Computed SERS spectra were generated using an adapted literature procedure.[11]
All calculations were carried out using Gaussian 16.[12] The initial input geometry was obtained from previously published models of NHCs on Au(111) surfaces.[11] The surfaces were modelled using a 34 atom cluster composed of two layers and an adatom. All 33 atoms in the layers were frozen during the optimisation, allowing only the ligand and adatom to relax. The surface was aligned to the xy plane, and the ligand positioned vertically on the surface as this conformation has been reported to contribute the most to the experimental SERS spectra (also giving origin to vibrations of interest at 800, 1300 and 1400 cm^{-1}).[11] Next, the geometry optimisation and frequency calculations were carried out using the BP86,[13,14] and the def2-SVP for light atoms (H to Br) and def2-TZVP basis sets with the associated def2 effective core potential for Au and I, with D3BJ empirical dispersion factor.[12,15–17] All structures were checked for lack of imaginary frequency in the .log file. The zz components of the polarizability derivatives with respect to each vibrational normal mode were then extracted using a python code and squared to give the raw SERS intensities. The computed SERS spectra were then generated by summing Lorentzian functions centred at the calculated vibrational frequencies, weighted by the corresponding Raman intensities.

A separate python code was then utilised to calculate for the atomic contributions for the desired vibration. For every atom i, the Cartesian displacement components $(d_{x,i}, d_{y,i}, d_{z,i})$ associated with the selected vibrational mode were extracted from the .log files. The magnitude of the displacement for each atom was calculated as the squared norm of the displacement vector:

$$D_i = d_{x,i}^2 + d_{y,i}^2 + d_{z,i}^2$$

to account for the mass-weighted nature of vibrational normal modes, each displacement magnitude was multiplied by the atomic mass $m_i$to obtain a mass-weighted contribution,

$$C_i = m_i D_i = m_i(d_{x,i}^2 + d_{y,i}^2 + d_{z,i}^2)$$

the relative contribution of each atom to the vibrational mode was then obtained by normalizing the individual contributions with respect to the total contribution of all atoms,

$$P_i = \frac{C_i}{\Sigma_j C_j} \times 100$$

the resulting percentages P_i quantify the relative of each atom in the selected normal mode.
Force constants and reduced masses were simply extracted from the .log file. Vibrations were visualised using py3Dmol.

S3.2 Frontier Molecular Orbital Analysis of Ligands

All calculations were carried out using Gaussian 16,[12] with PBE0 hybrid functional,[18] def2-TZVP basis set,[15,16] and D3BJ empirical dispersion factor.[17] All structures were checked for lack of imaginary frequency. The orbitals were visualised using Avogadro.[19]

S3.3 Energy Decomposition Analysis Natural Orbitals for Chemical Valence Analysis

All calculations were carried out using Orca version 6.1.1.[20,21] The geometries obtained in section S3.1 were extracted and the surface reduced to a 20 Au atom cluster for computational efficiency, composed of 2 layers and an adatom.
Single-point energies and energy decomposition analyses (EDA–NOCV) were computed using the hybrid functional PBE0,[18] with D3BJ empirical dispersion factor.[17] Scalar relativistic effects were included via the ZORA Hamiltonian,[20] with the ZORA-def2-TZVP basis set for light atoms (H to Br) and the SARC-ZORA-TZVP basis set, together with the corresponding SARC/J auxiliary basis for Au and I,[16,22] with frozen core. The resolution-of-the-identity (RI) approximation,[23] and the chain-of-spheres exchange (COSX) algorithm were employed for efficient evaluation of Coulomb and exchange integrals.[24,25] EDA–NOCV was carried out by partitioning the system into two fragments (ligand as one and surface and adatom as the second) at the same level of theory, allowing decomposition of the interaction energy into electrostatic, Pauli repulsion, and orbital contributions.

S4 Correlations Between σ_p and Characteristic NHC–SAMs vibrations

Other vibrations have been shown to contain some contributions from the C–Au.[11] These are shown below.

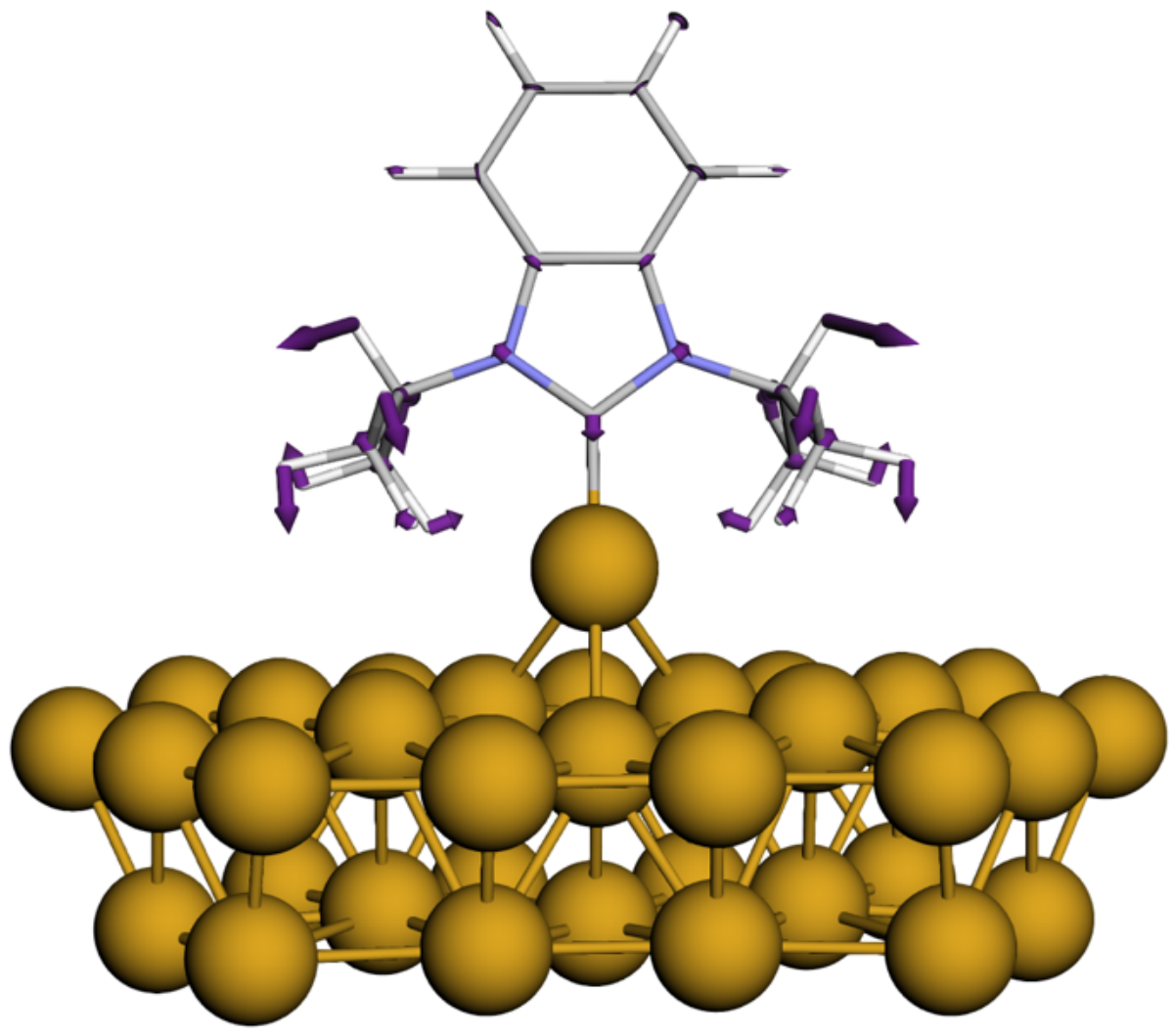

Figure S1 Computed ν_1 for R = H, at 1286 cm^{-1} (experimental value is 1295 cm^{-1}).

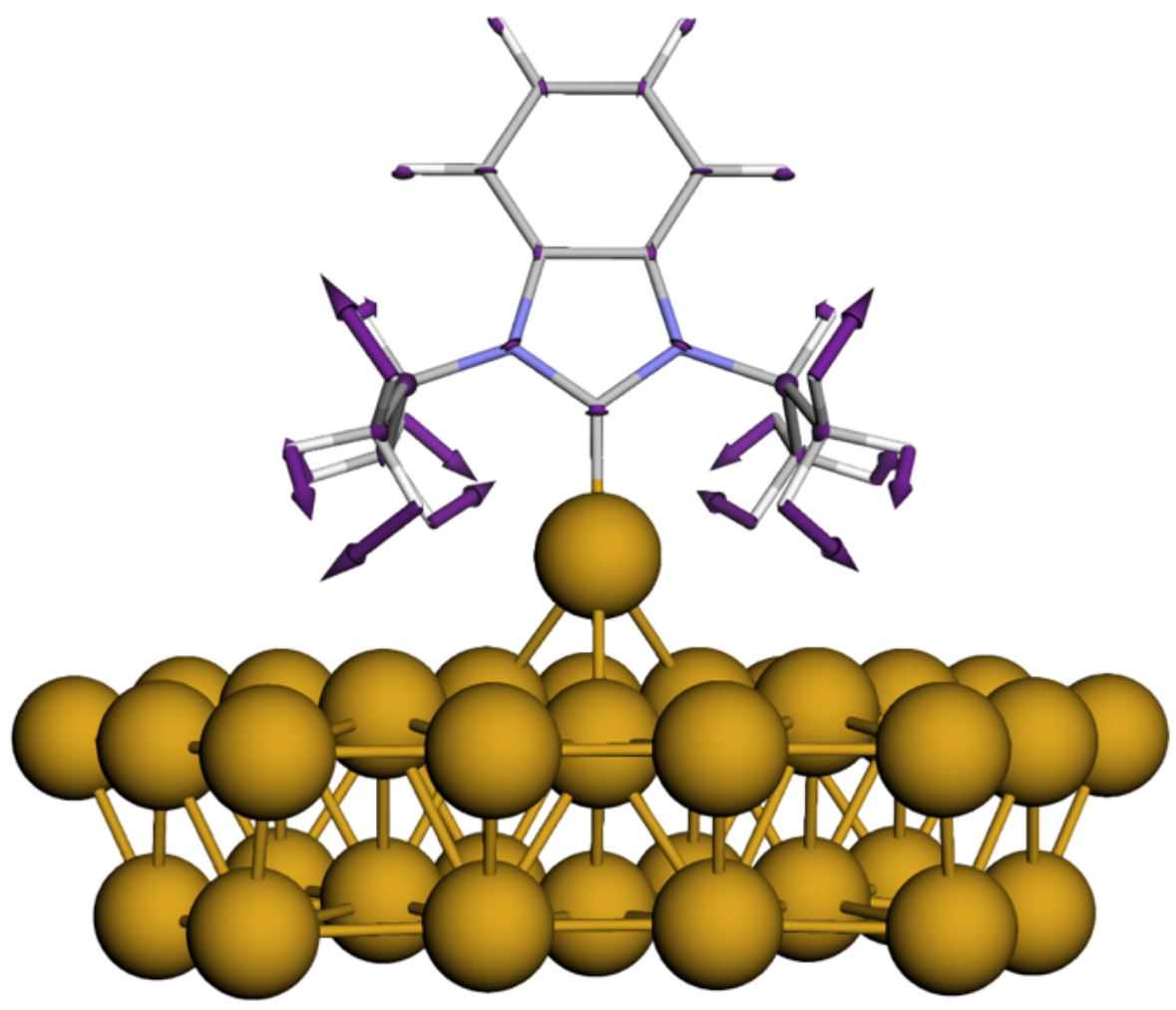

Figure S2 Computed ν_2 for R = H, at 1401 cm^{-1} (experimental value is 1404 cm^{-1}).

Table S1 σ_p versus other characteristic vibrations for NHC–SAMs.

R	σ_p	σ_m	σ_{p-}	σ_{p+}	ν_{C-Au} / cm^{-1}	ν_1 / cm^{-1}	ν_2 / cm^{-1}
NH_2	-0.66	-0.16	-0.15	-1.30	800	1308	1411
OMe	-0.27	0.12	-0.26	-0.78	794	1295	1404
H	0.00	0.00	0.00	0.00	803	1295	1404
F	0.06	0.34	-0.03	-0.07	800	1298	1406
I	0.18	0.35	0.27	0.14	818	1293	1401
Br	0.23	0.39	0.25	0.15	821	1293	1401
CHO	0.42	0.35	1.03	0.73	800	1295	1404
CO_2Me	0.45	0.37	0.75	0.49	803	1295	1404
CO_2H	0.45	0.37	0.77	0.42	797	1295	1404
CN	0.66	0.56	1.00	0.66	791	1298	1404
NO_2	0.81	0.71	1.27	0.79	815	1295	1409

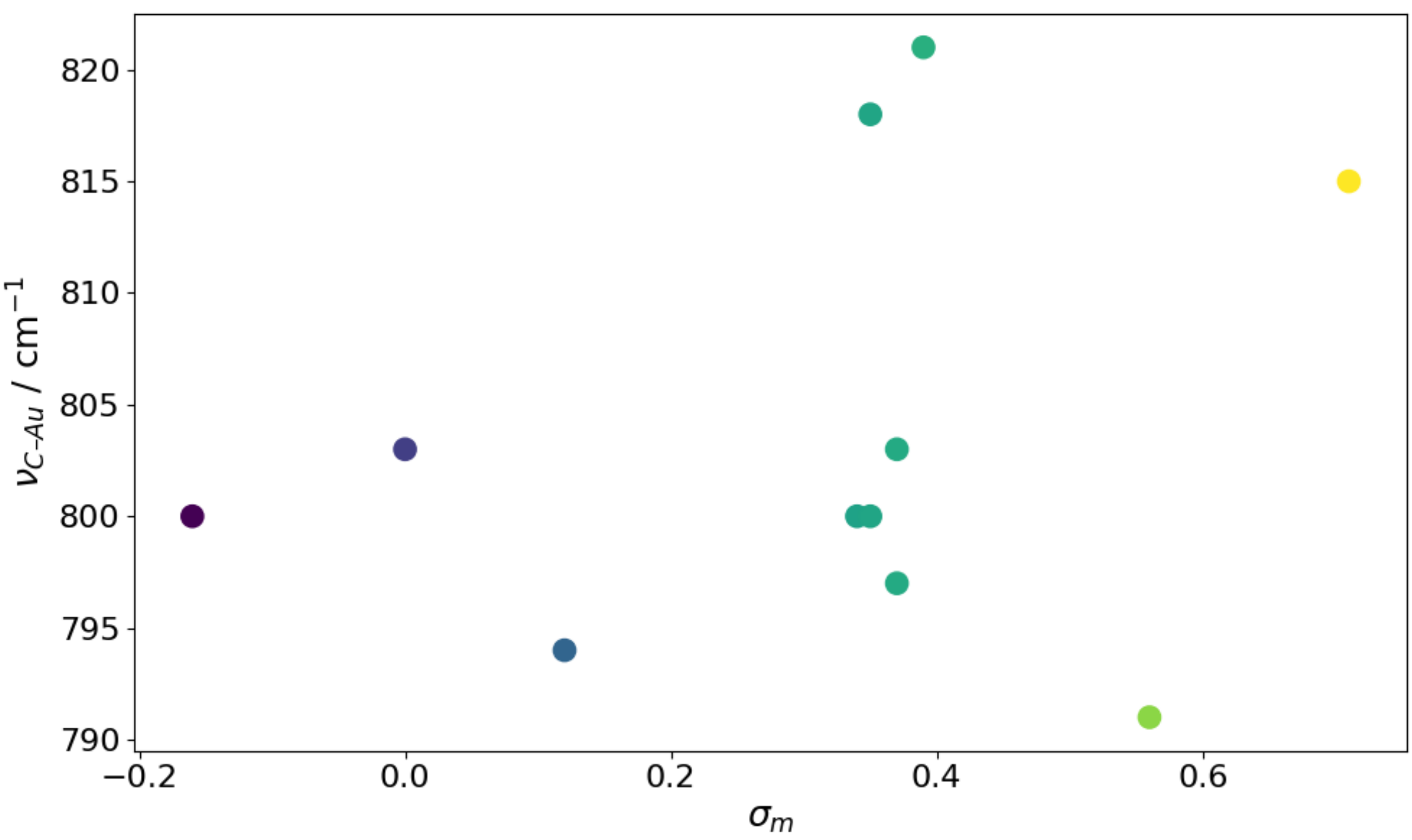

Figure S3 σ_m versus ν_{C-Au}.

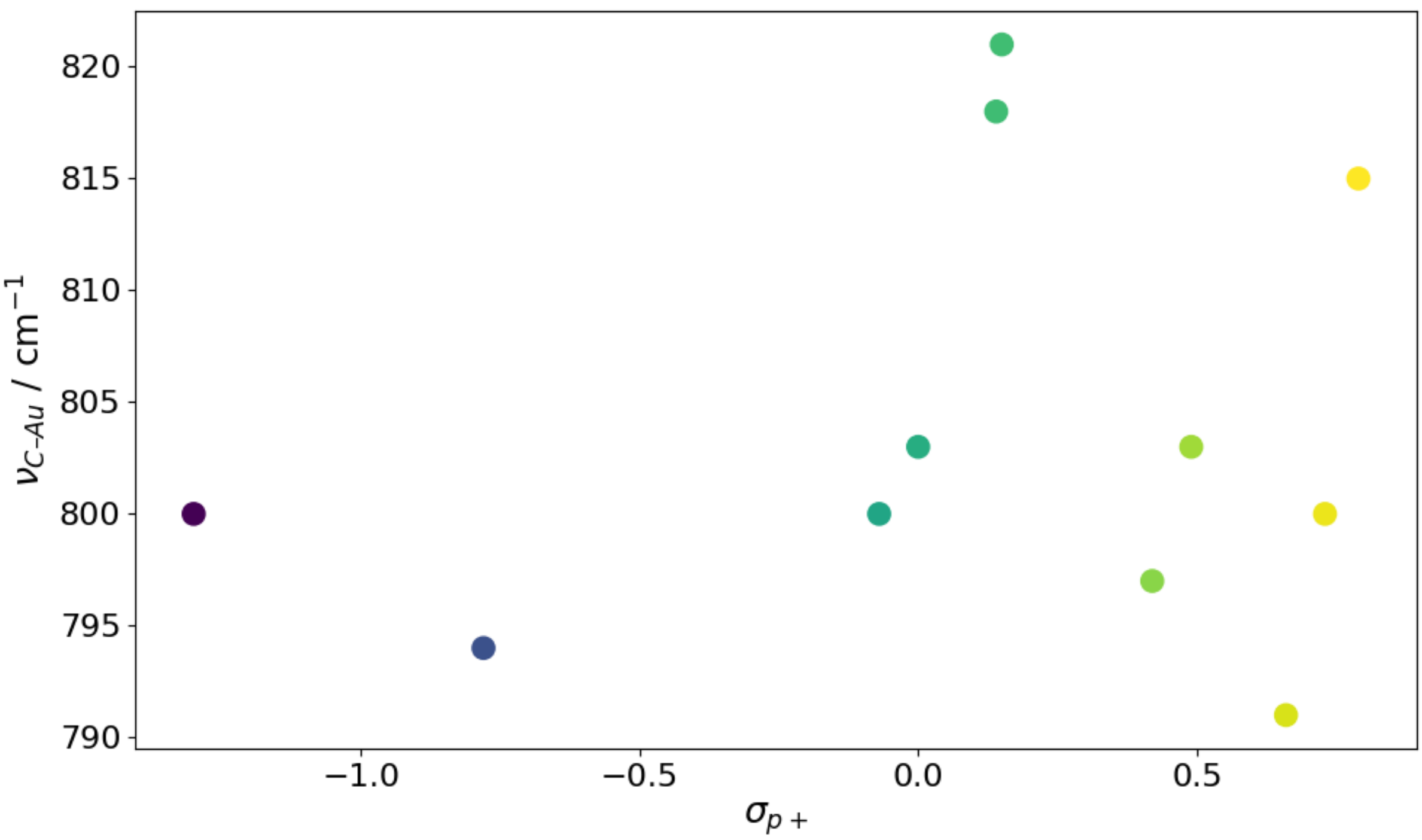

Figure S4 σ_{p+} versus $\nu_{C\text{–}Au}$.

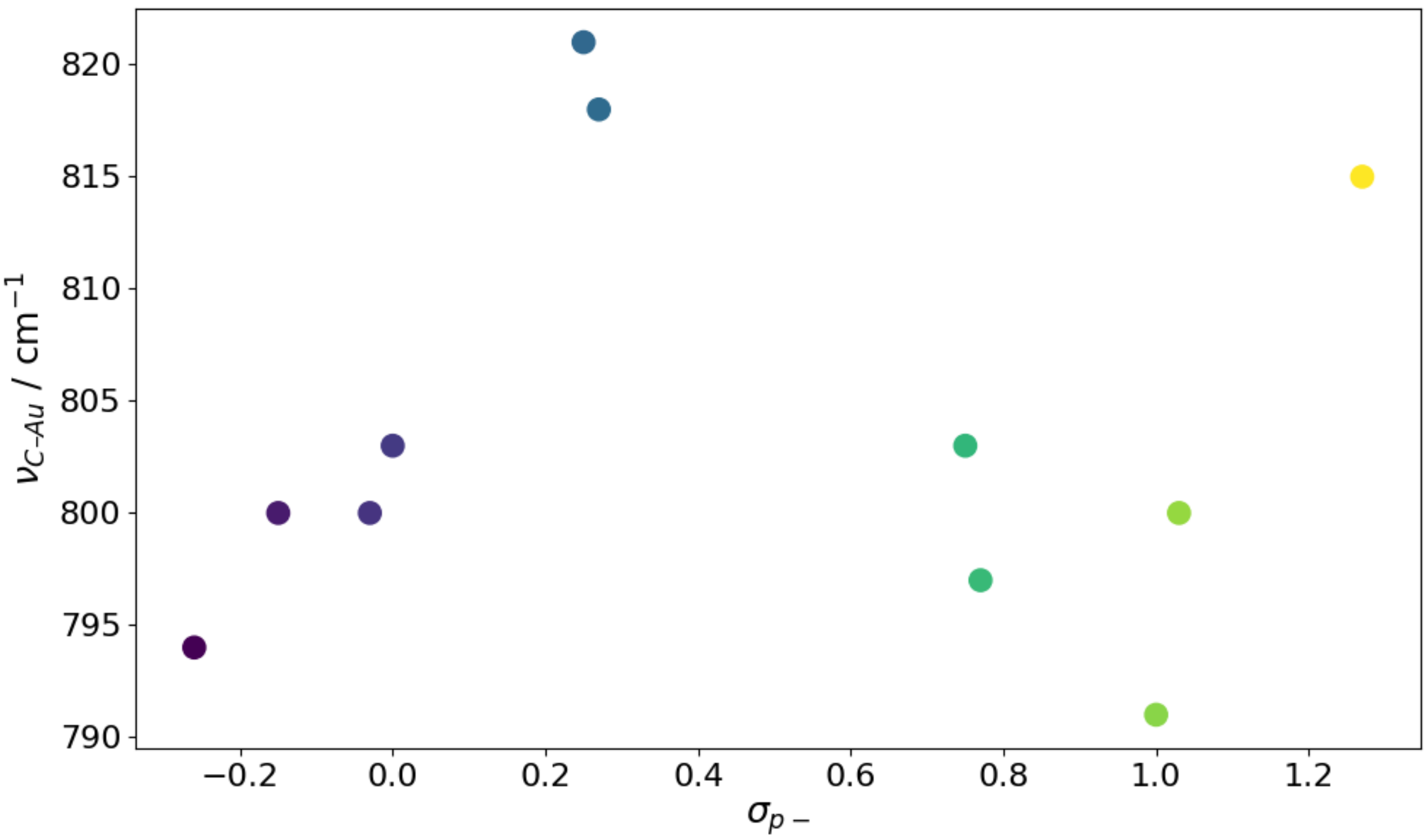

Figure S5 σ_{p-} versus $\nu_{C\text{–}Au}$.

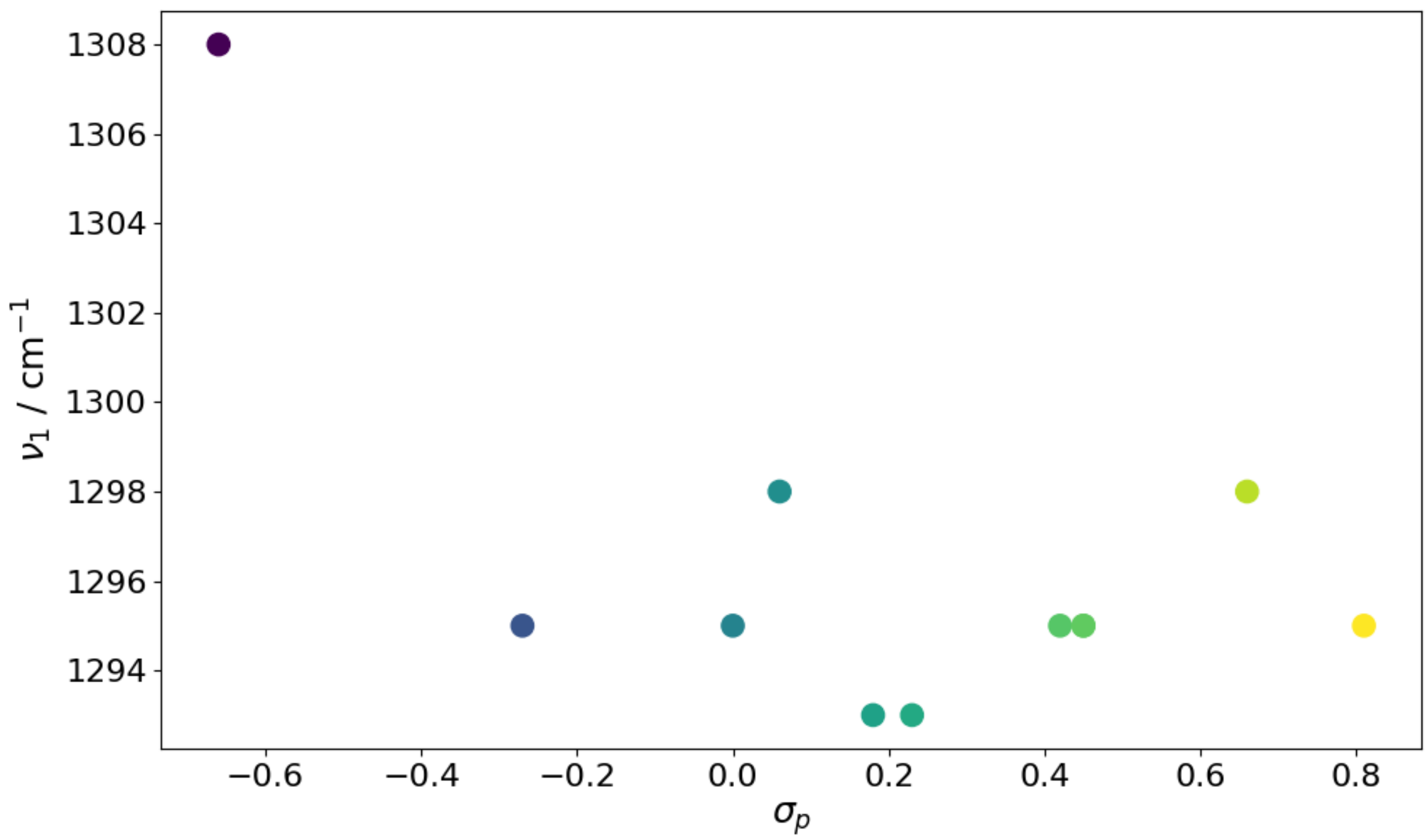

Figure S6 σ_p versus ν_1.

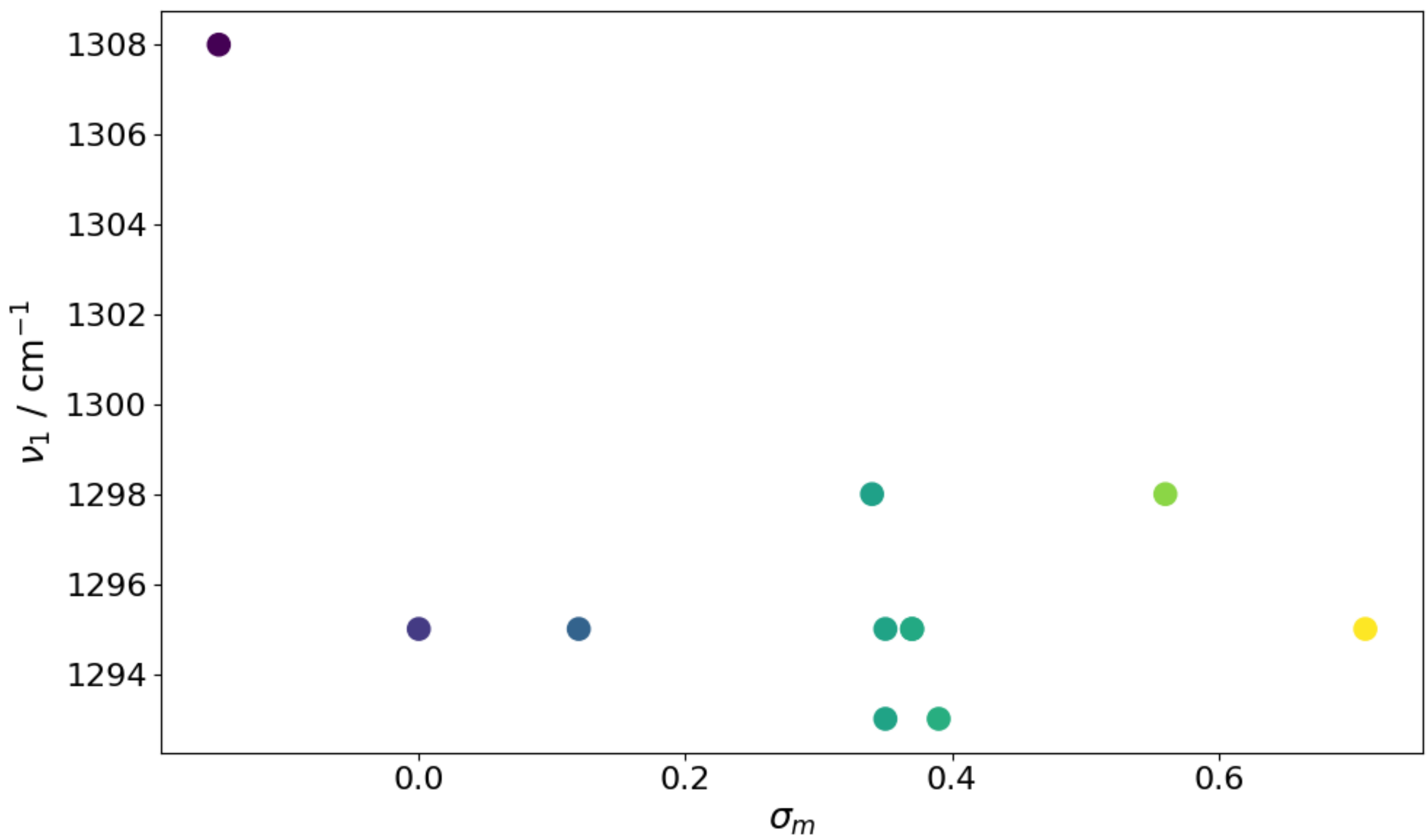

Figure S7 σ_m versus ν_1.

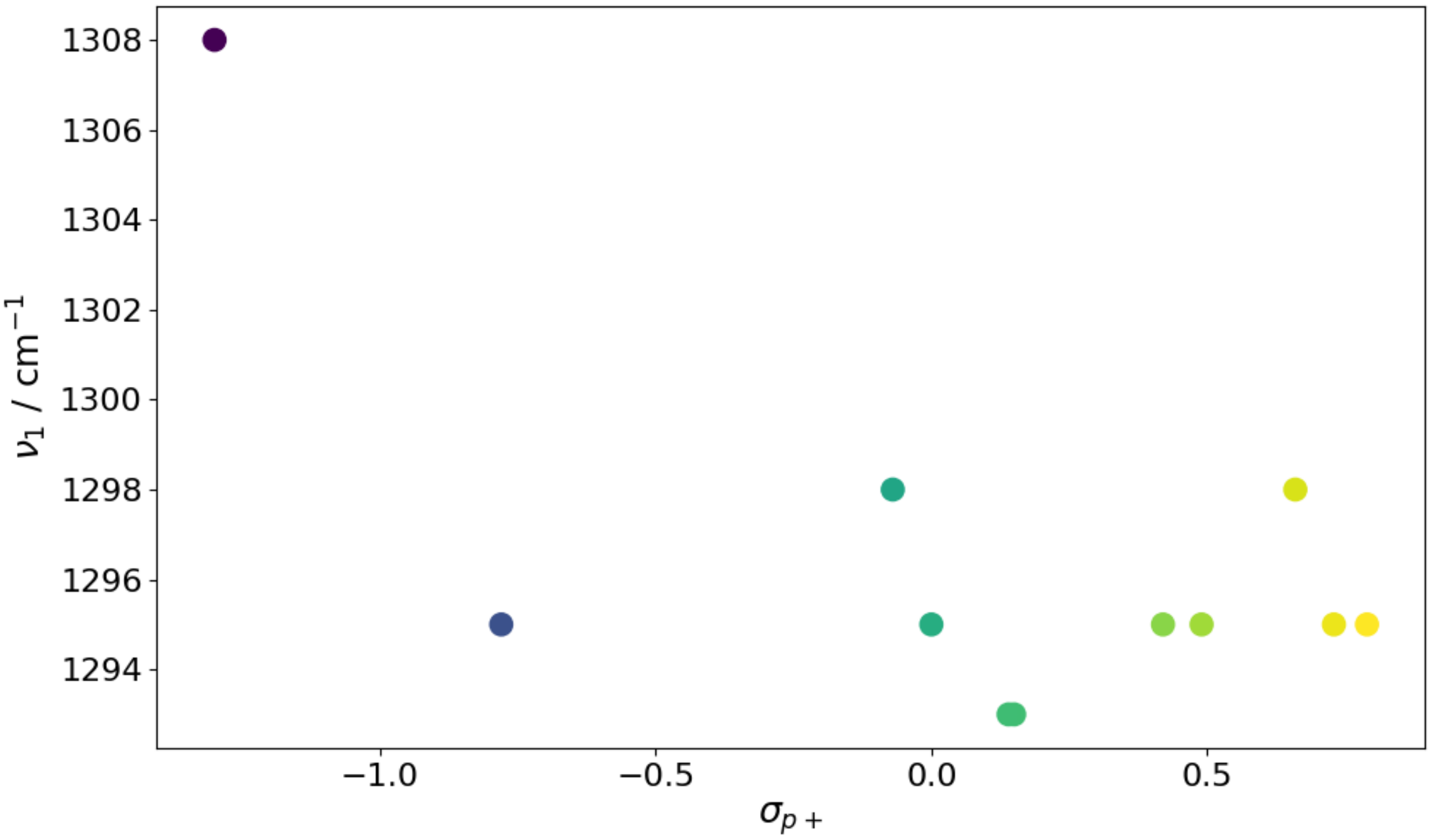

Figure S8 σ_{p+} versus ν_1.

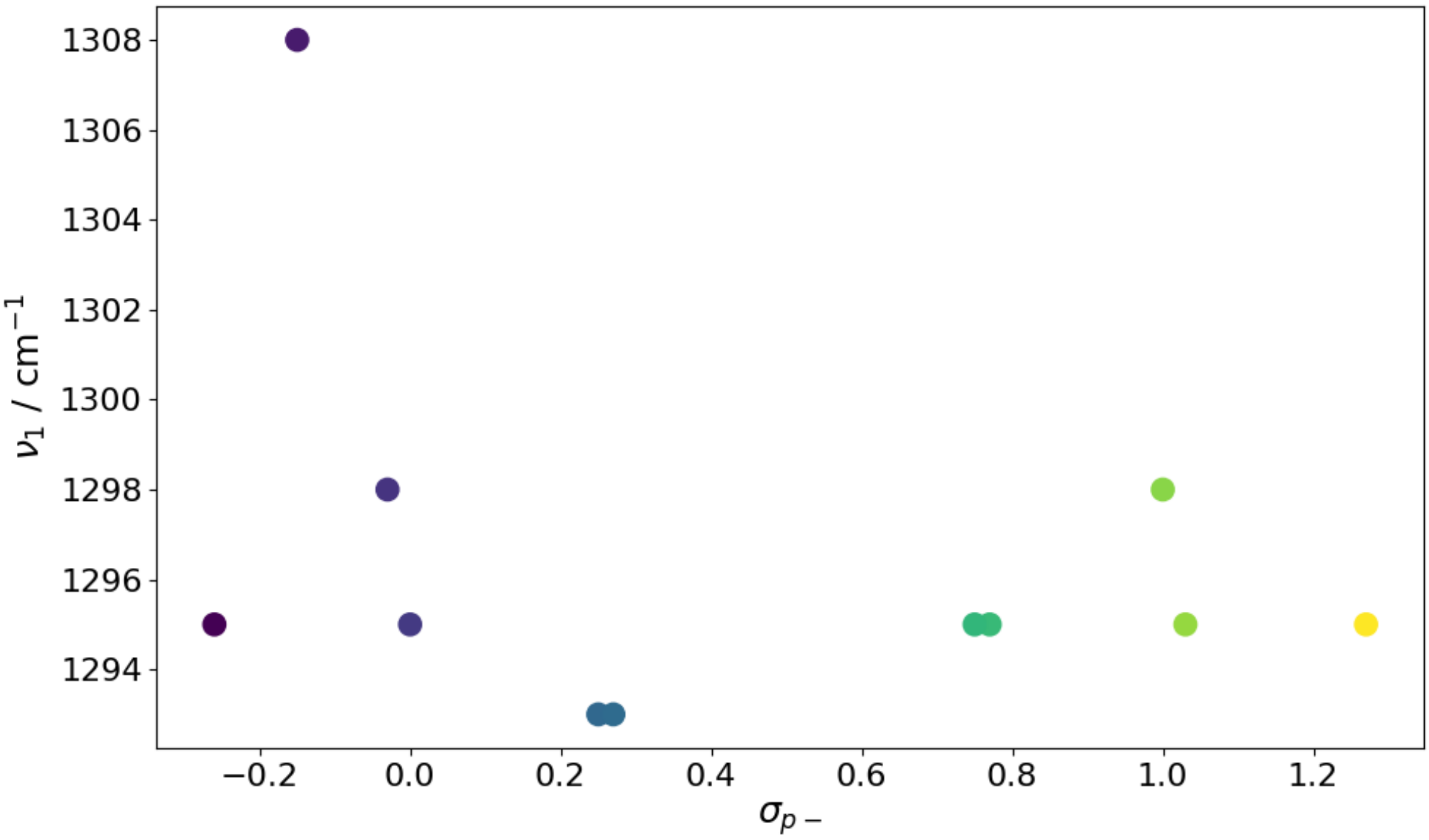

Figure S9 σ_{p-} versus ν_1.

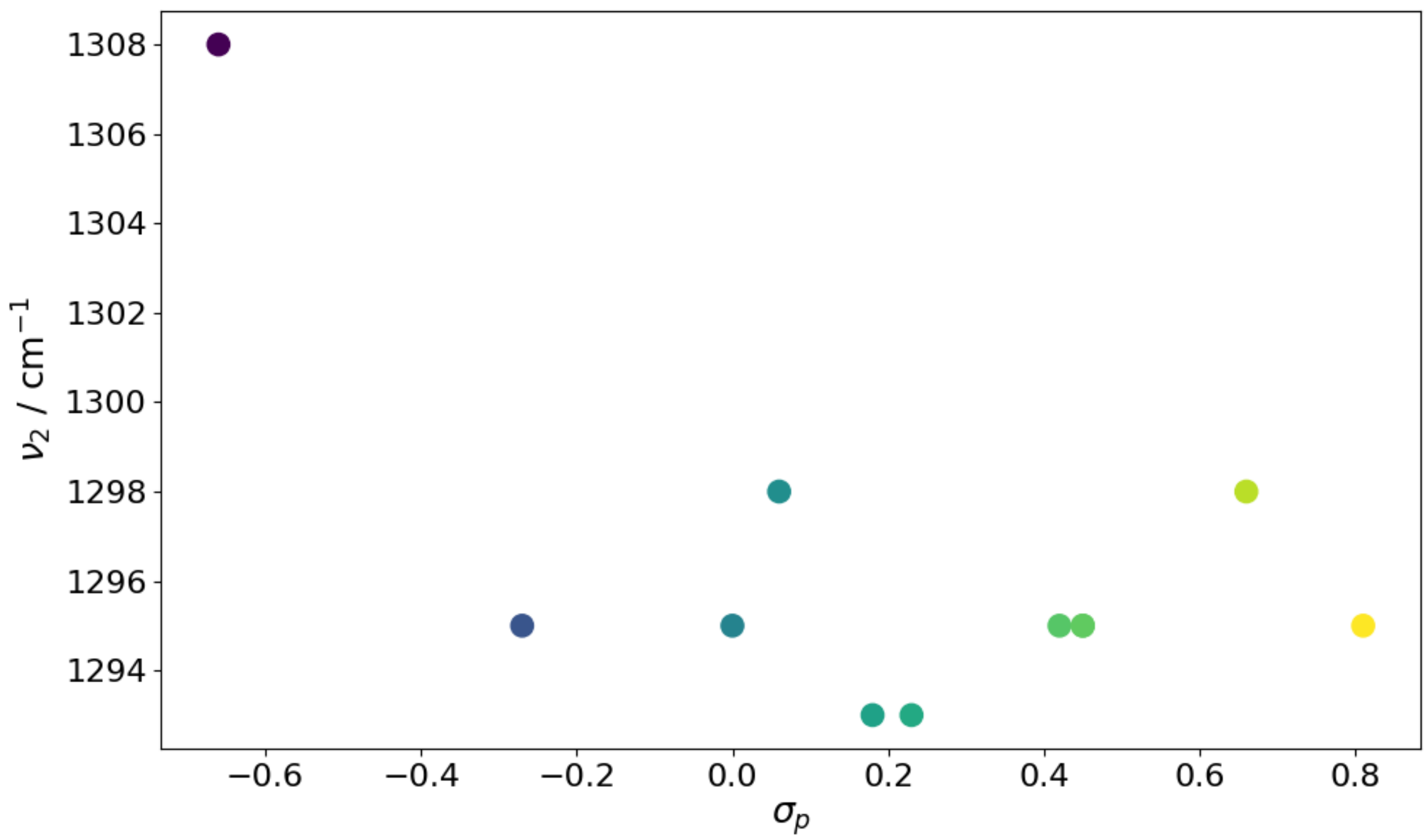

Figure S10 σ_p versus ν_2.

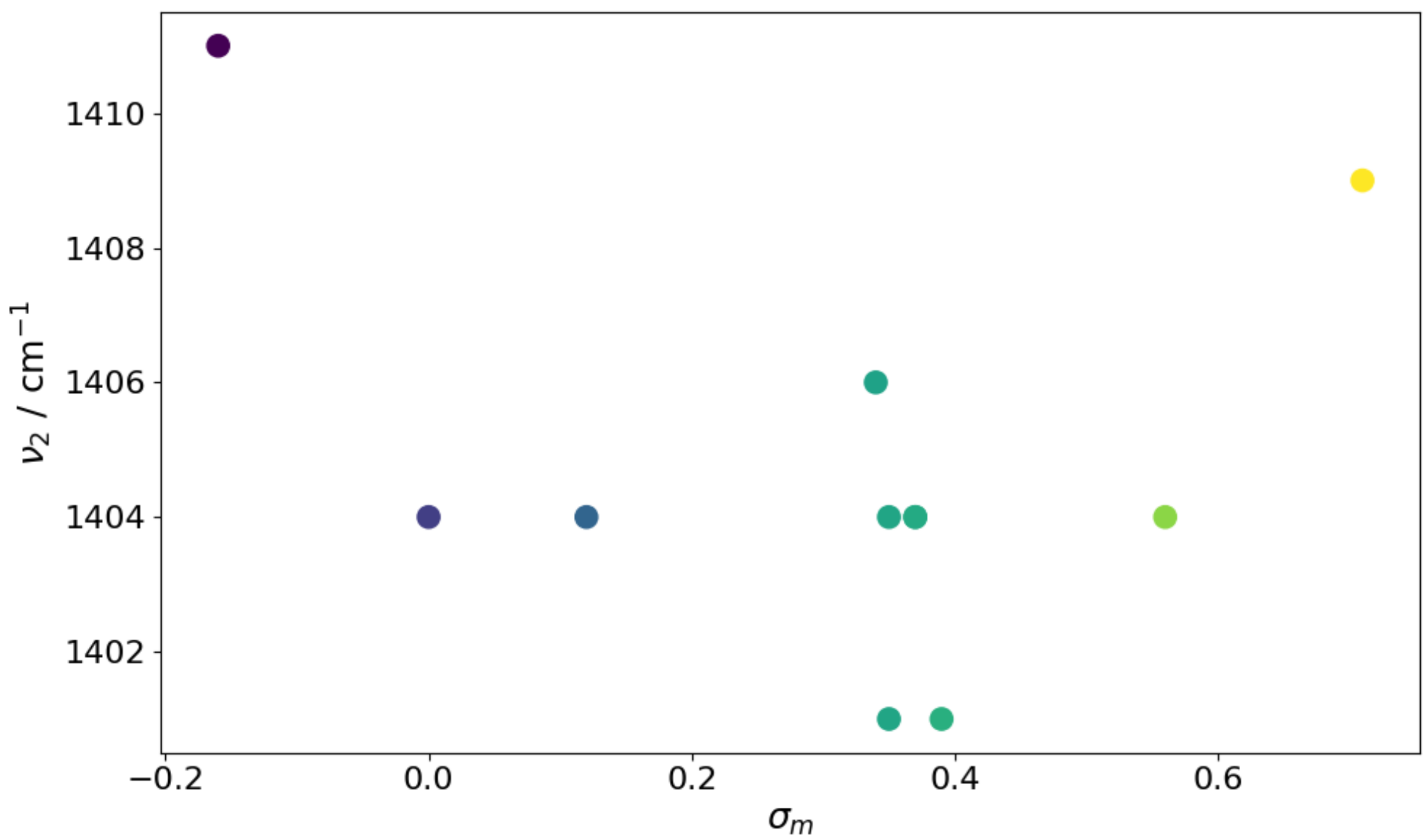

Figure S11 σ_m versus ν_2.

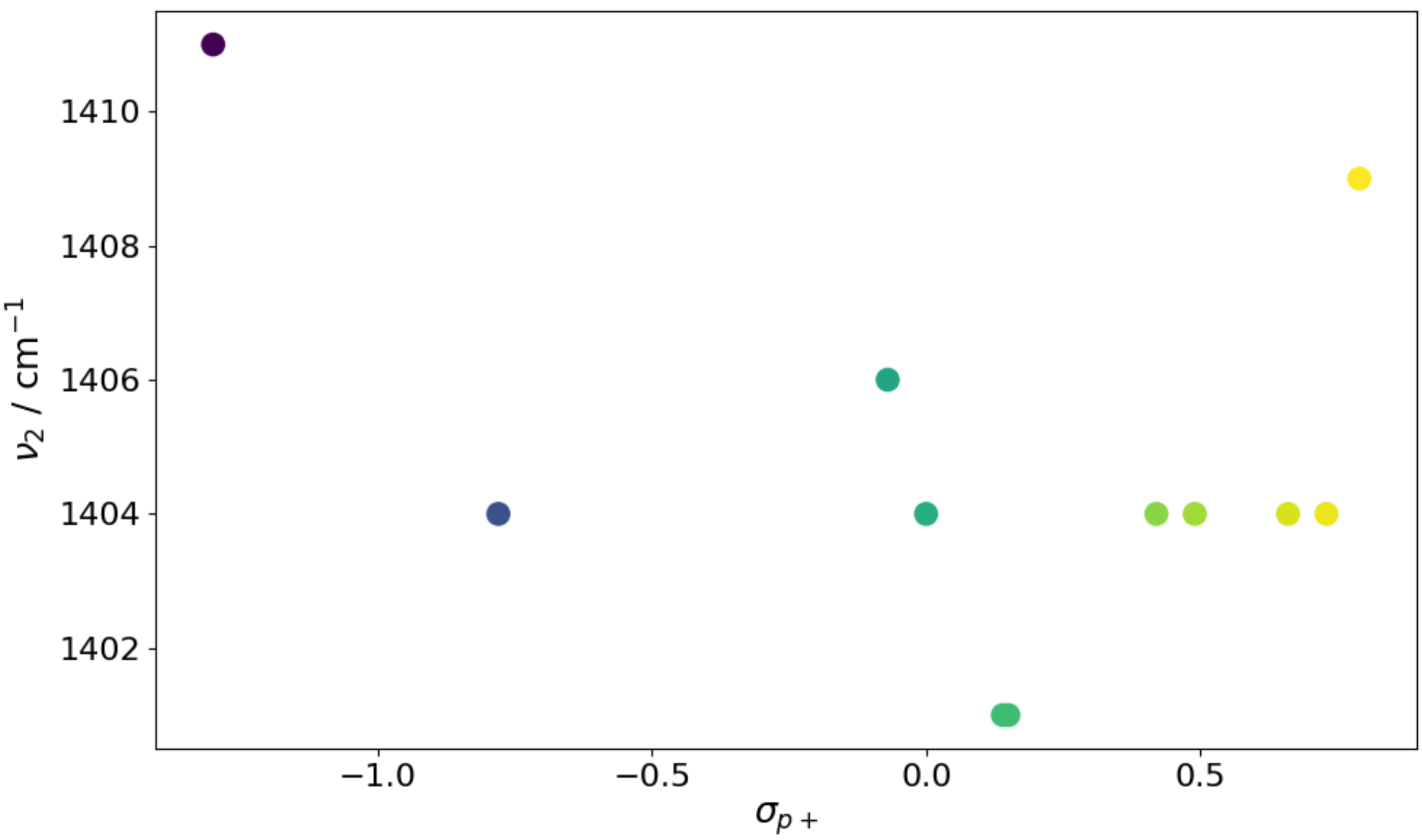

Figure S12 σ_{p+} versus ν_2.

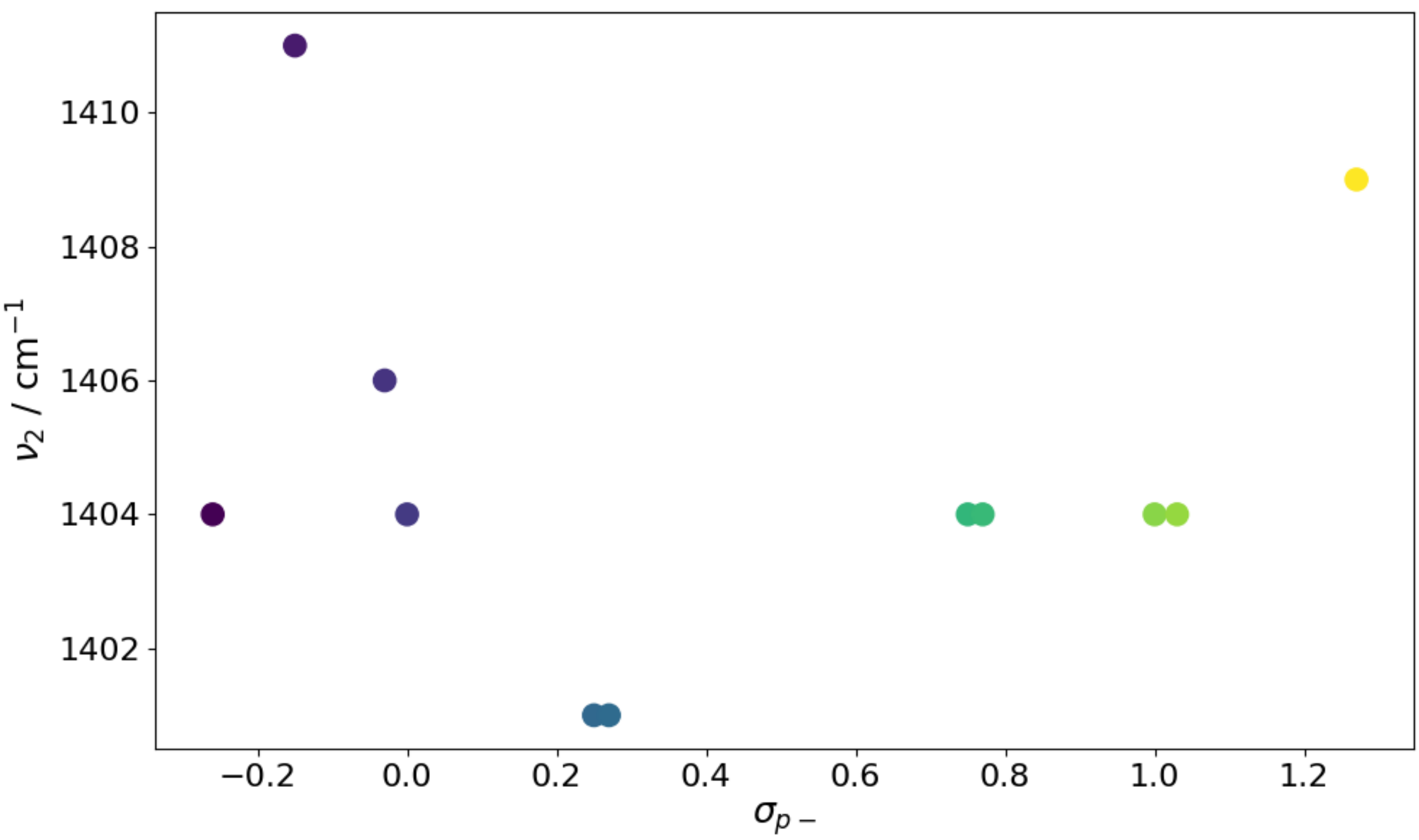

Figure S13 σ_{p-} versus ν_2.

S5 Computed SERS Spectra

Note: because of the Lorentzian broadening and the peak detection function in Python the C–Au peak in the computed SERS spectra might be slightly shifted compared to the value reported in the .log file.

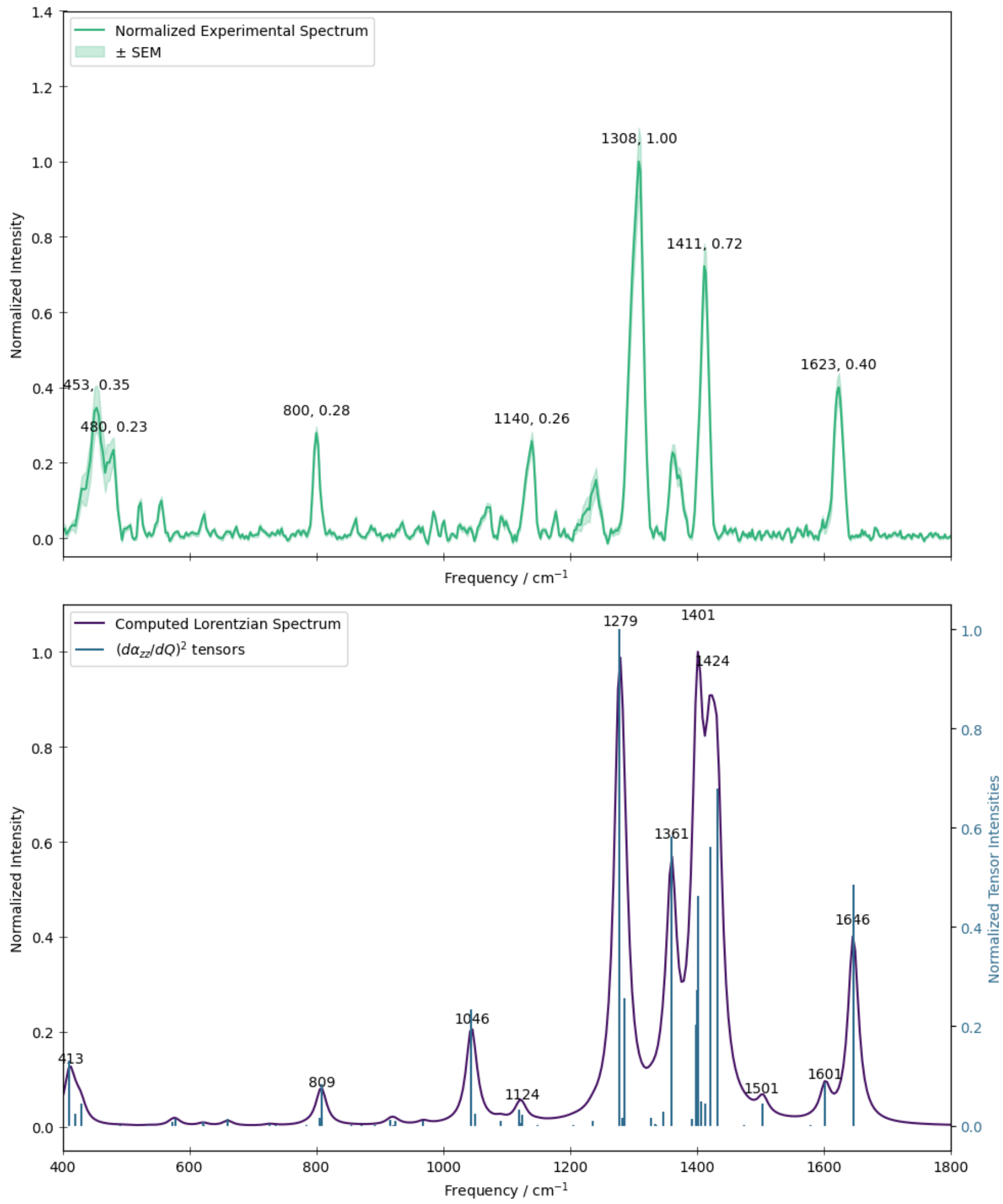

Figure S14 Experimental (top) versus predicted (bottom) SERS spectra for R = NH_2.

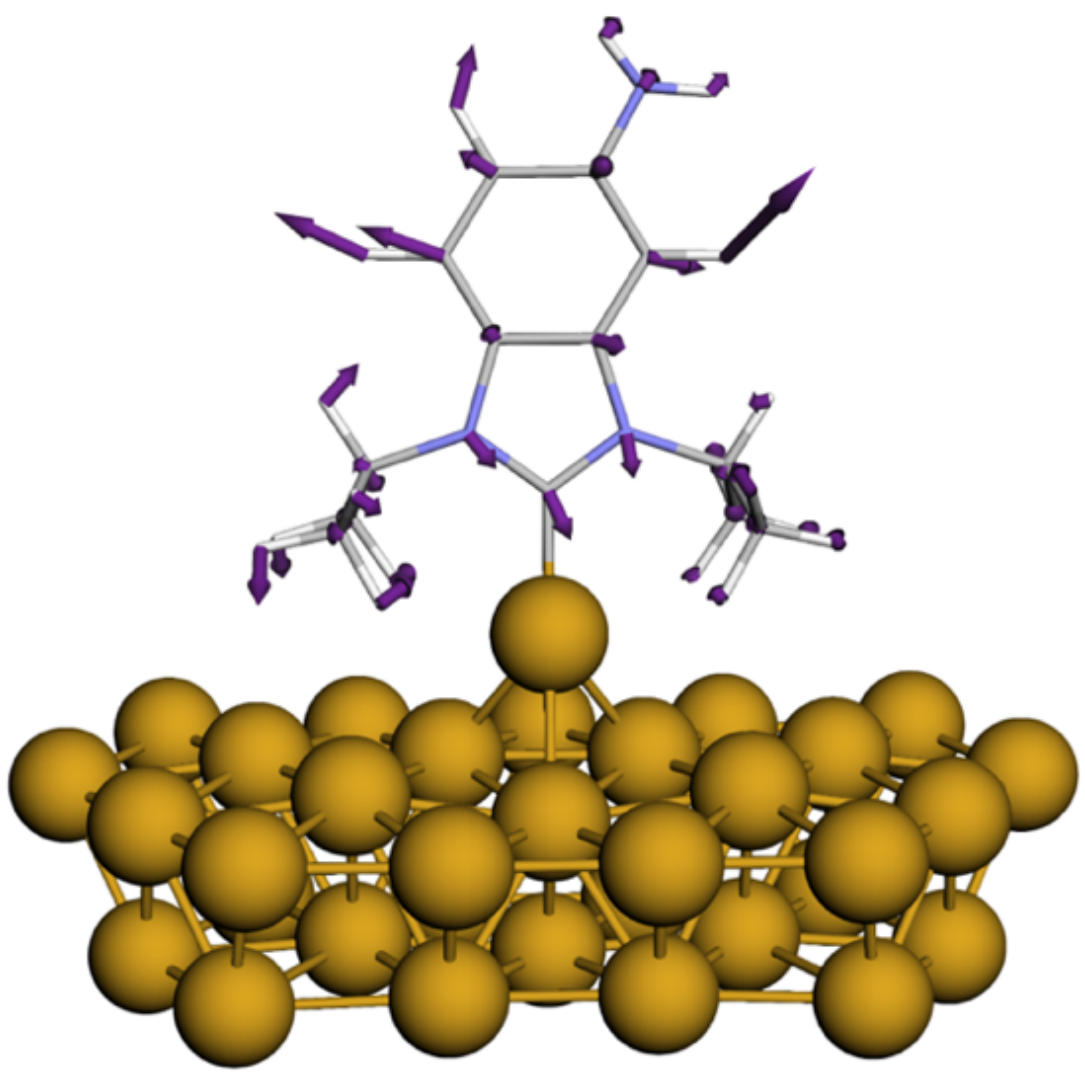

Figure S15 Vibration at 808 cm^{-1} for R = NH_2.

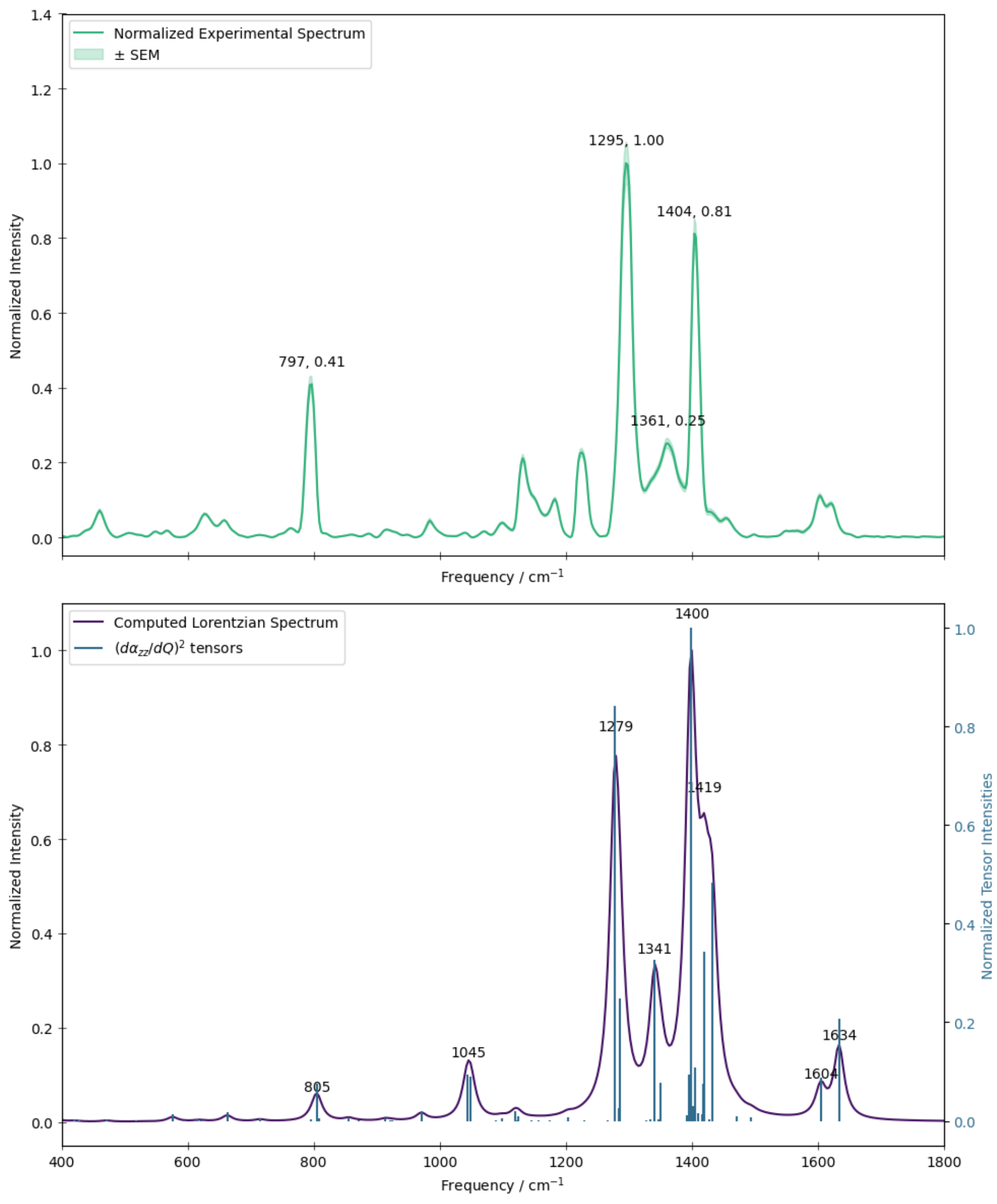

Figure S16 Experimental (top) versus predicted (bottom) SERS spectra for R = OMe.

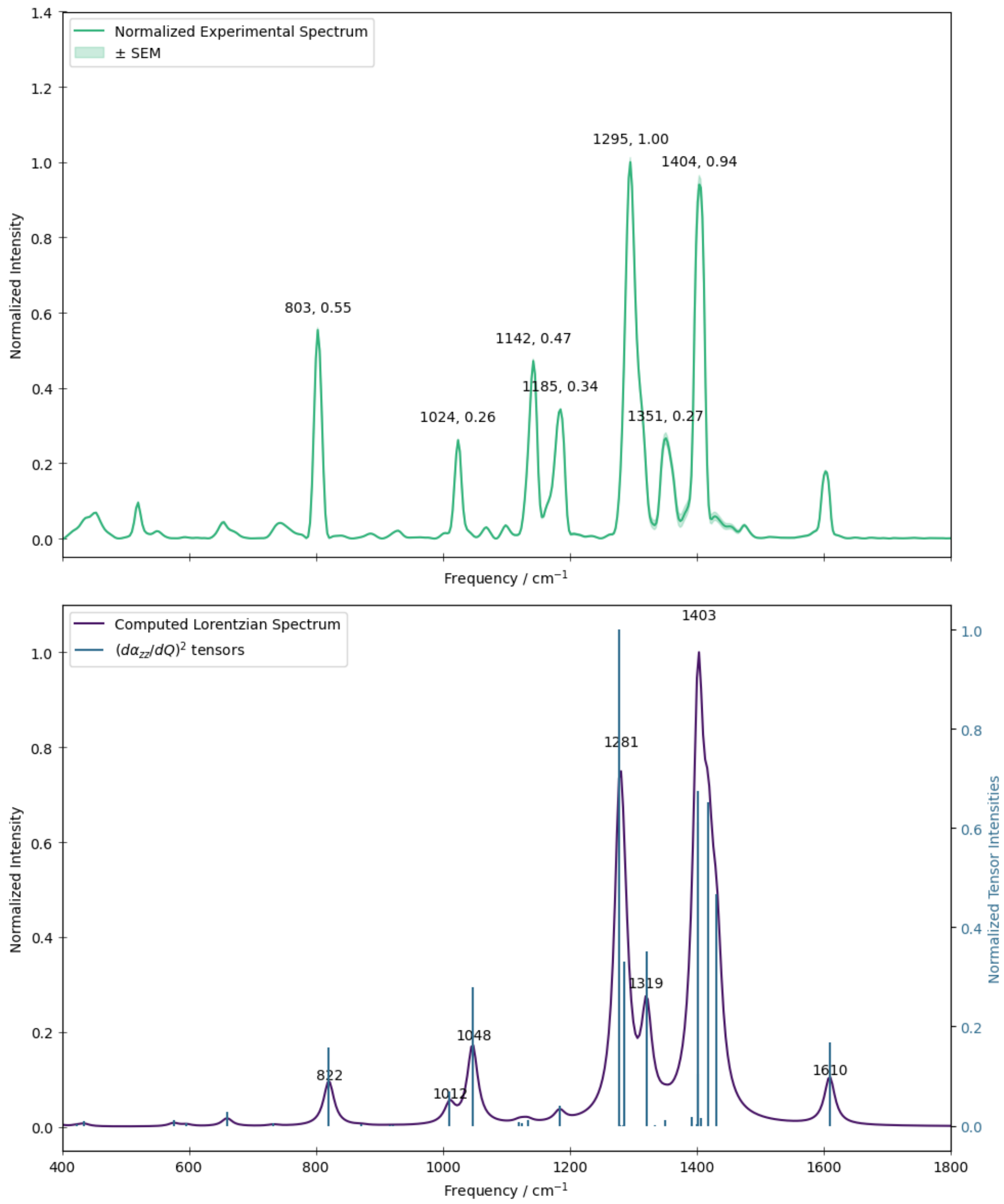

Figure S17 Experimental (top) versus predicted (bottom) SERS spectra for R = H.

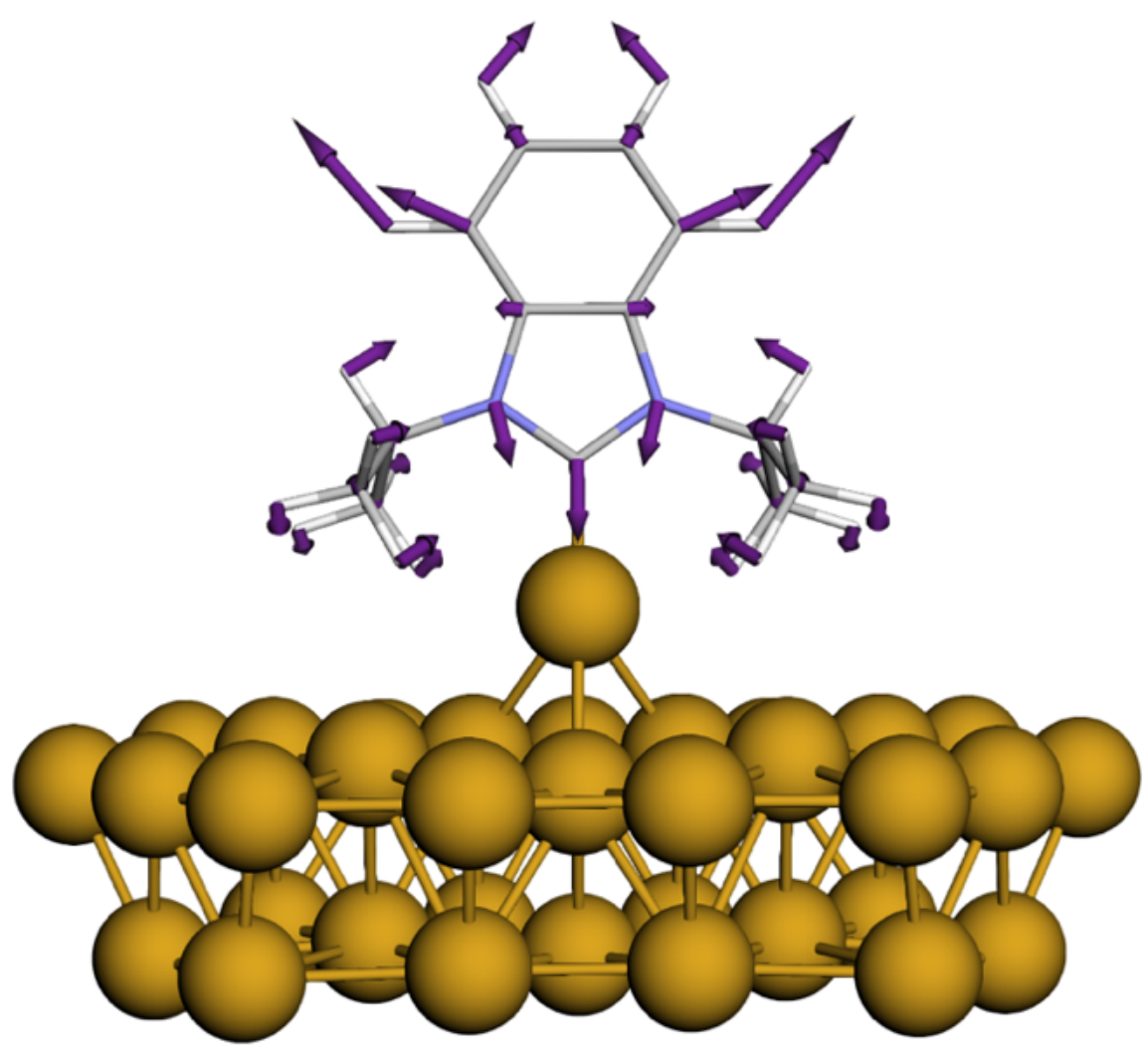

Figure S18 Vibration at 821 cm^{-1} for R = H.

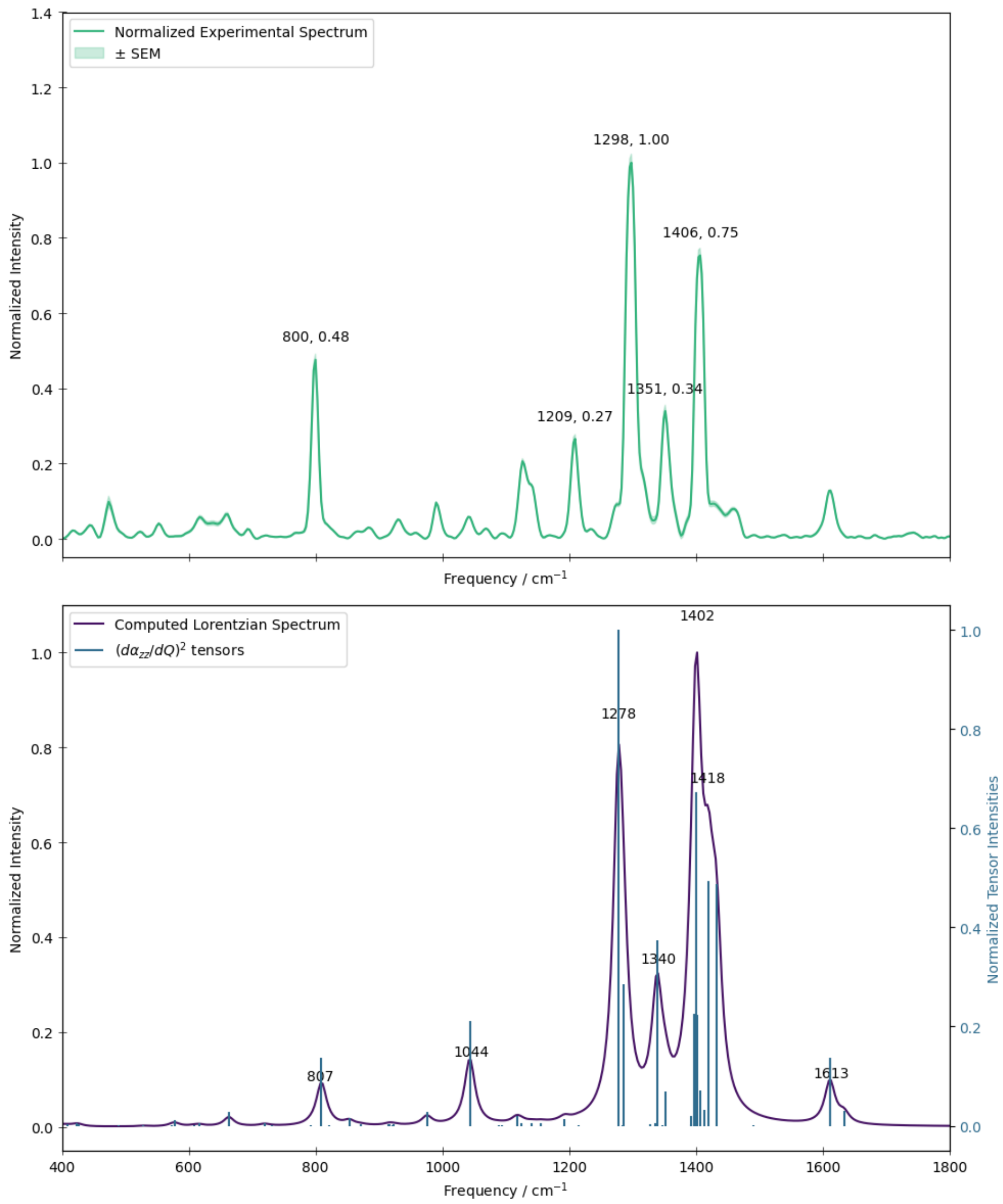

Figure S19 Experimental (top) versus predicted (bottom) SERS spectra for R = H.

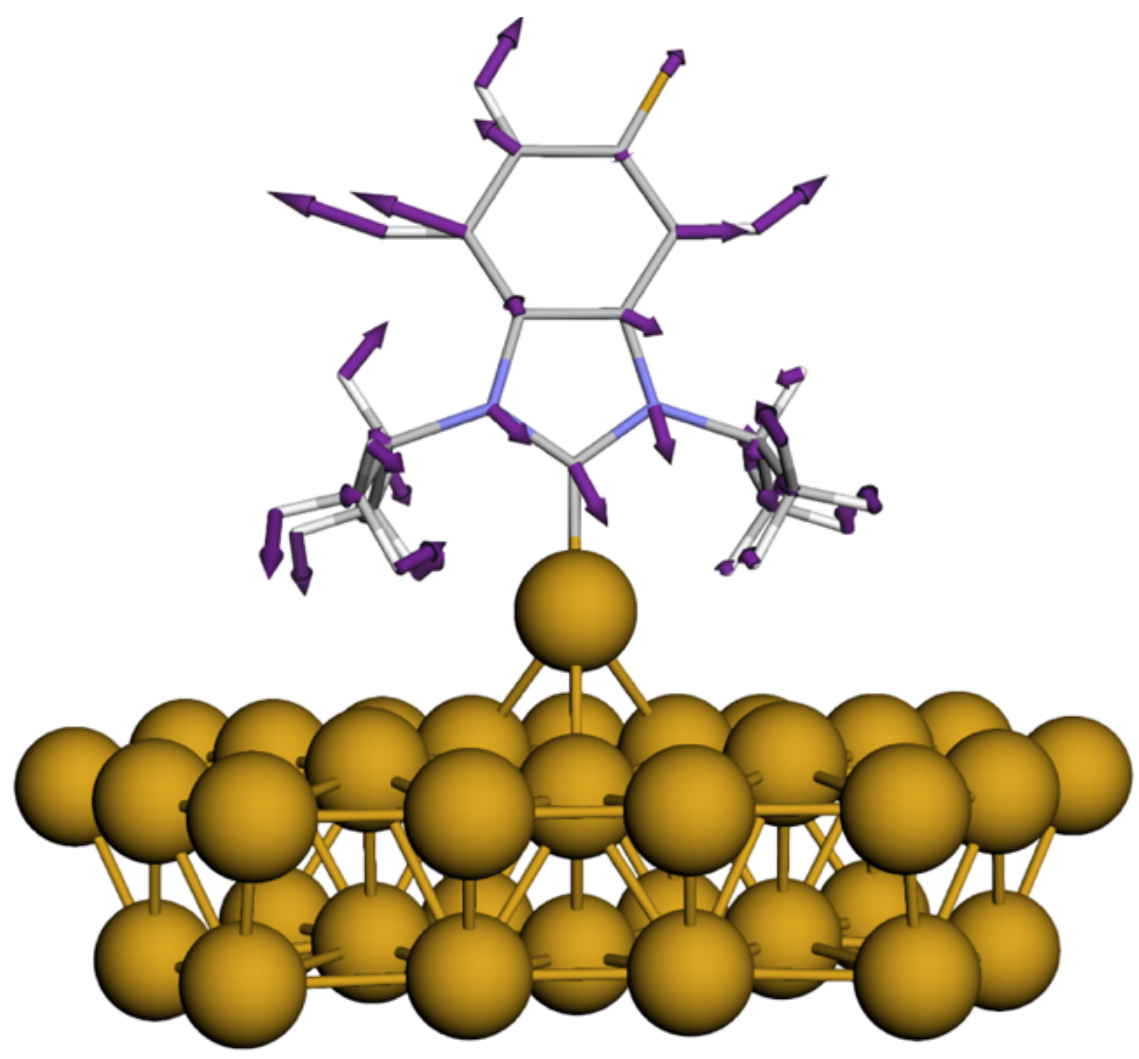

Figure S20 Vibration at 809 cm^{-1} for R = F.

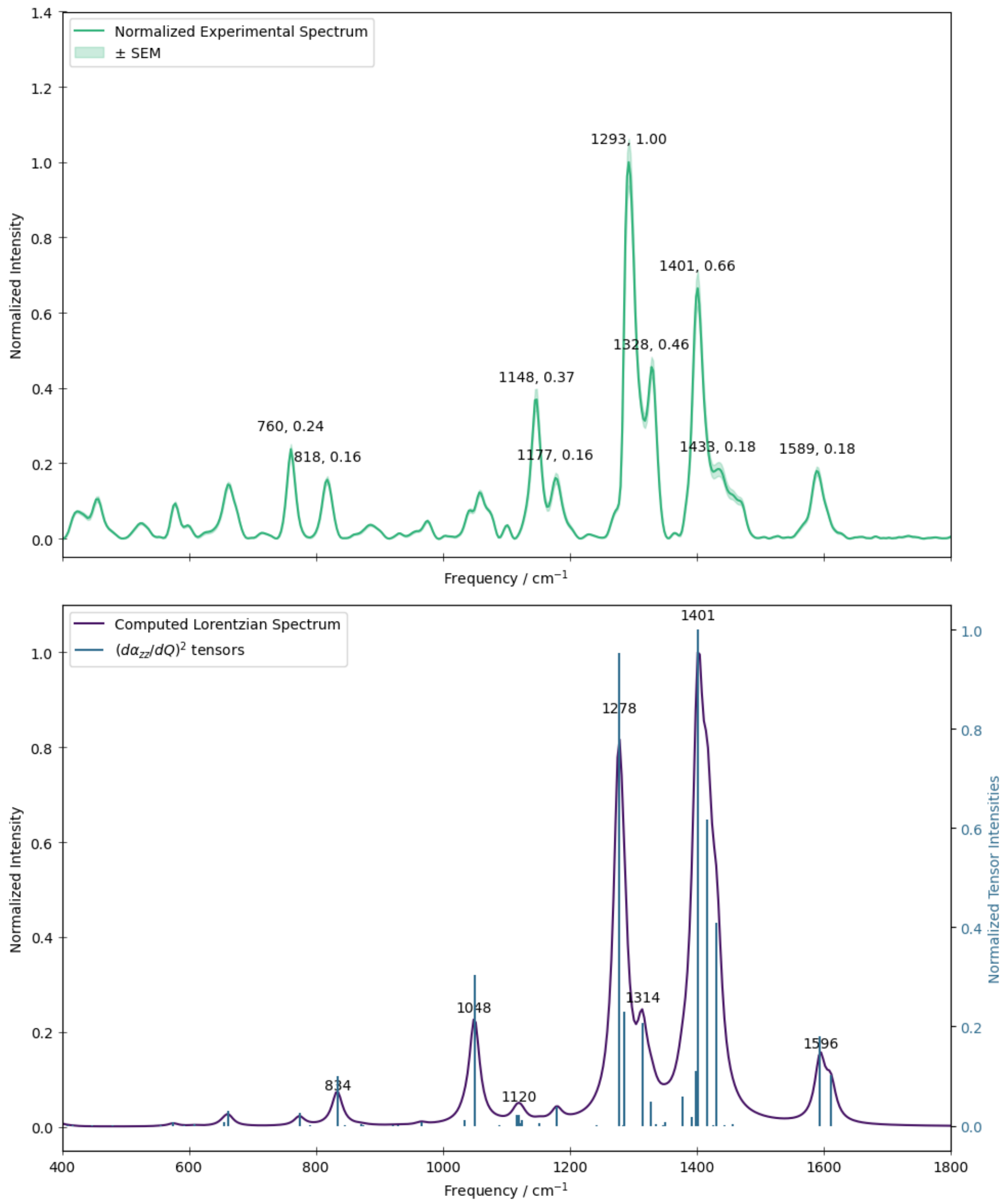

Figure S21 Experimental (top) versus predicted (bottom) SERS spectra for R = I.

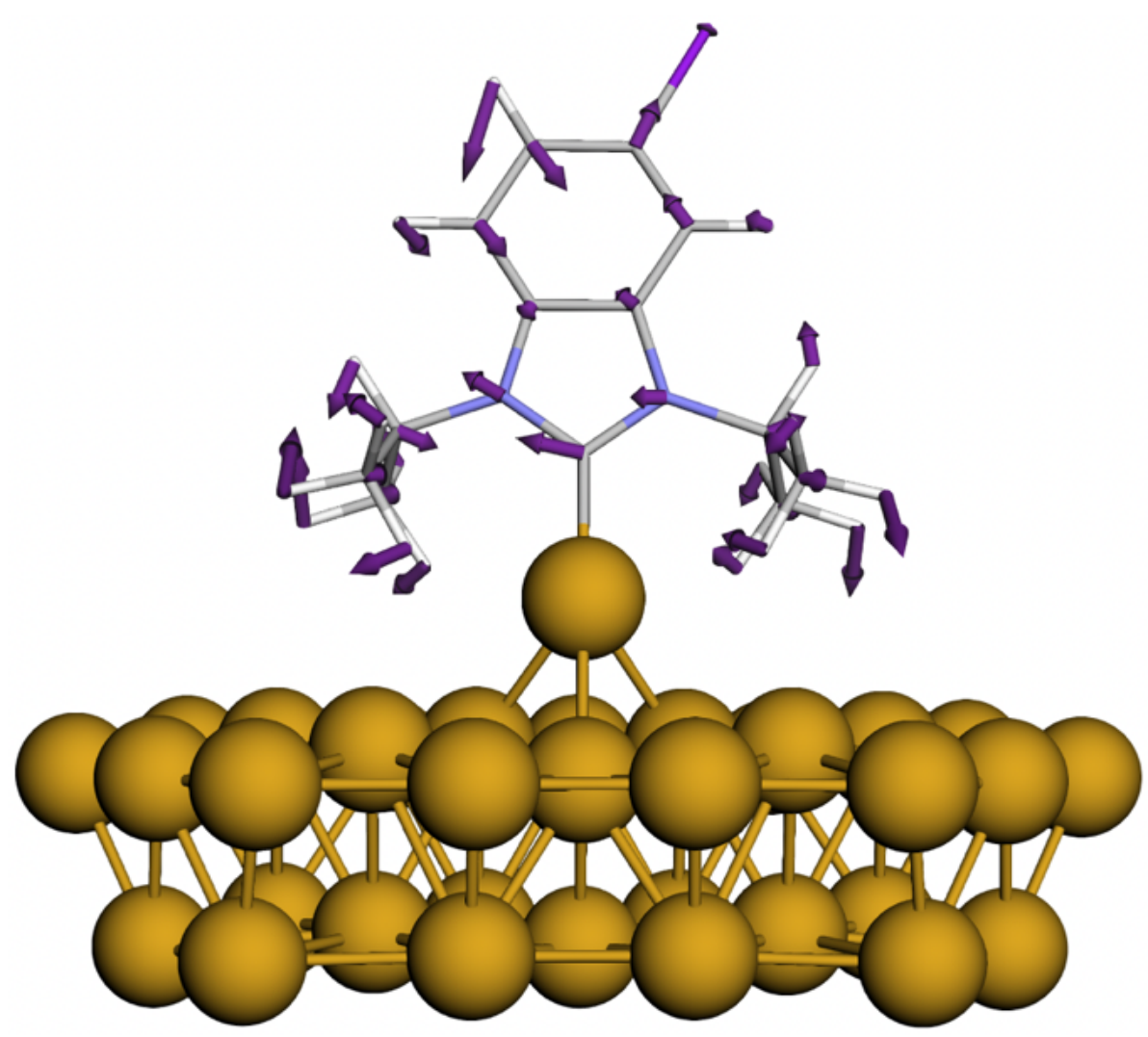

Figure S22 Vibration at 794 cm^{-1} for R = I.

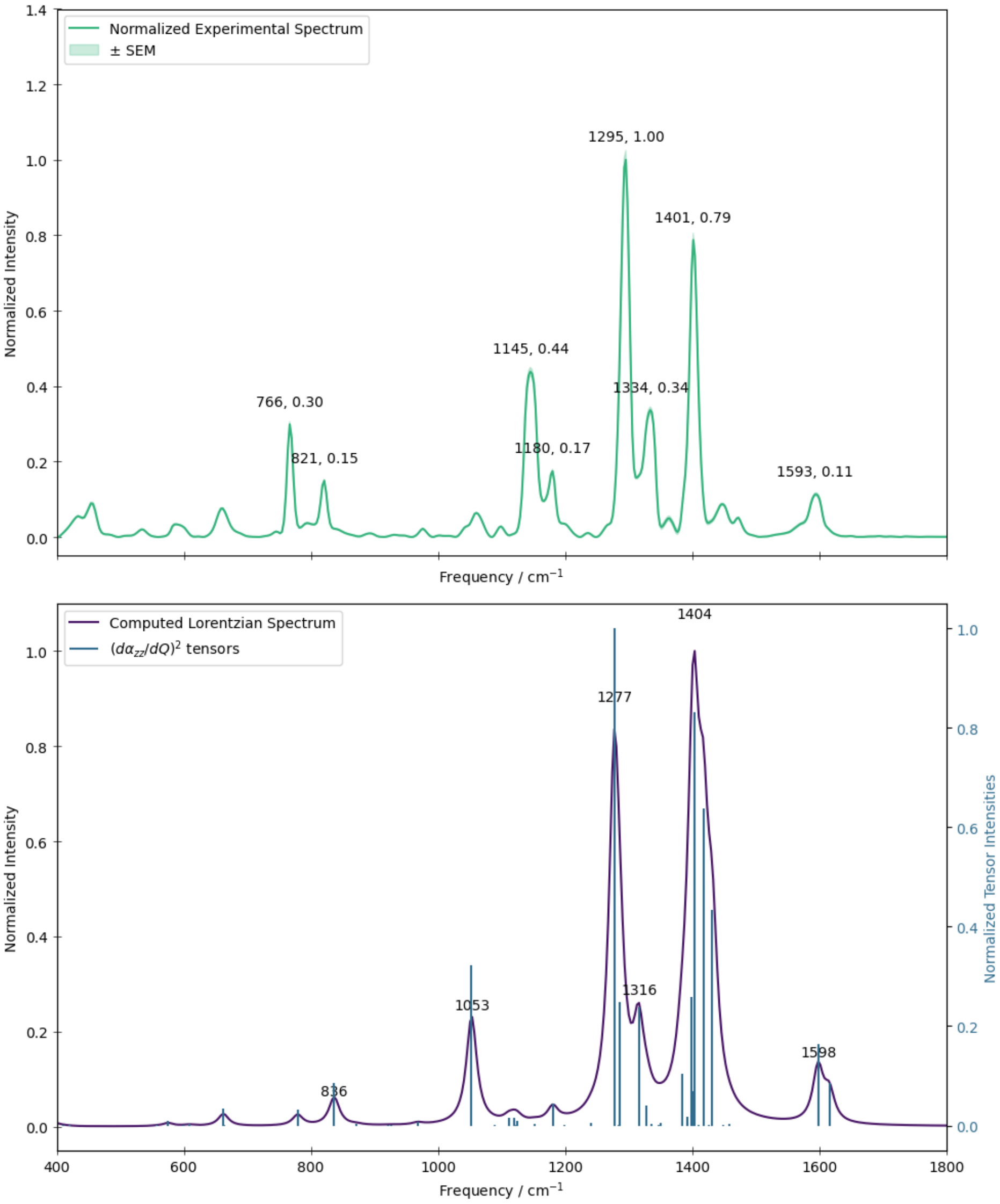

Figure S23 Experimental (top) versus predicted (bottom) SERS spectra for R = Br.

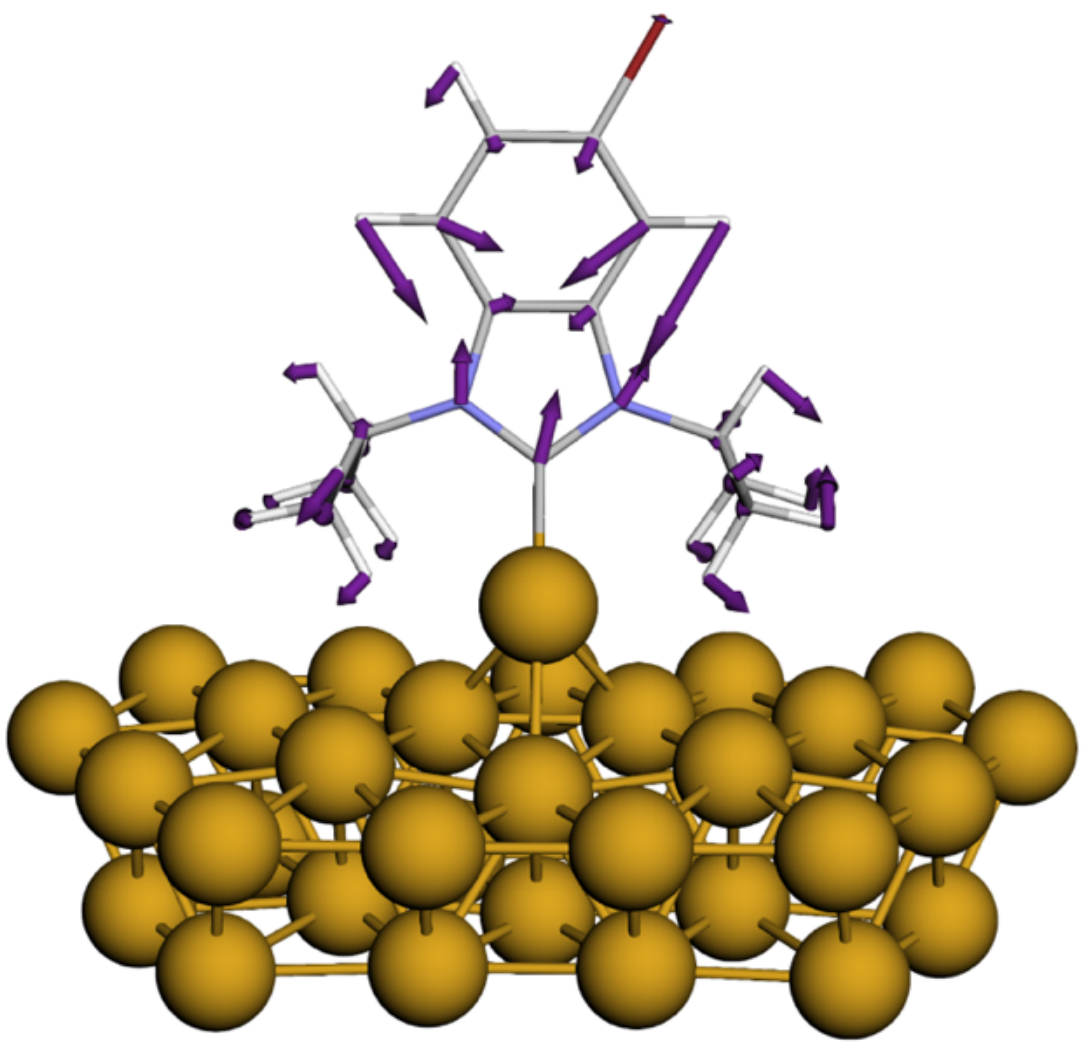

Figure S24 Vibration at 837 cm^{-1} for R = Br.

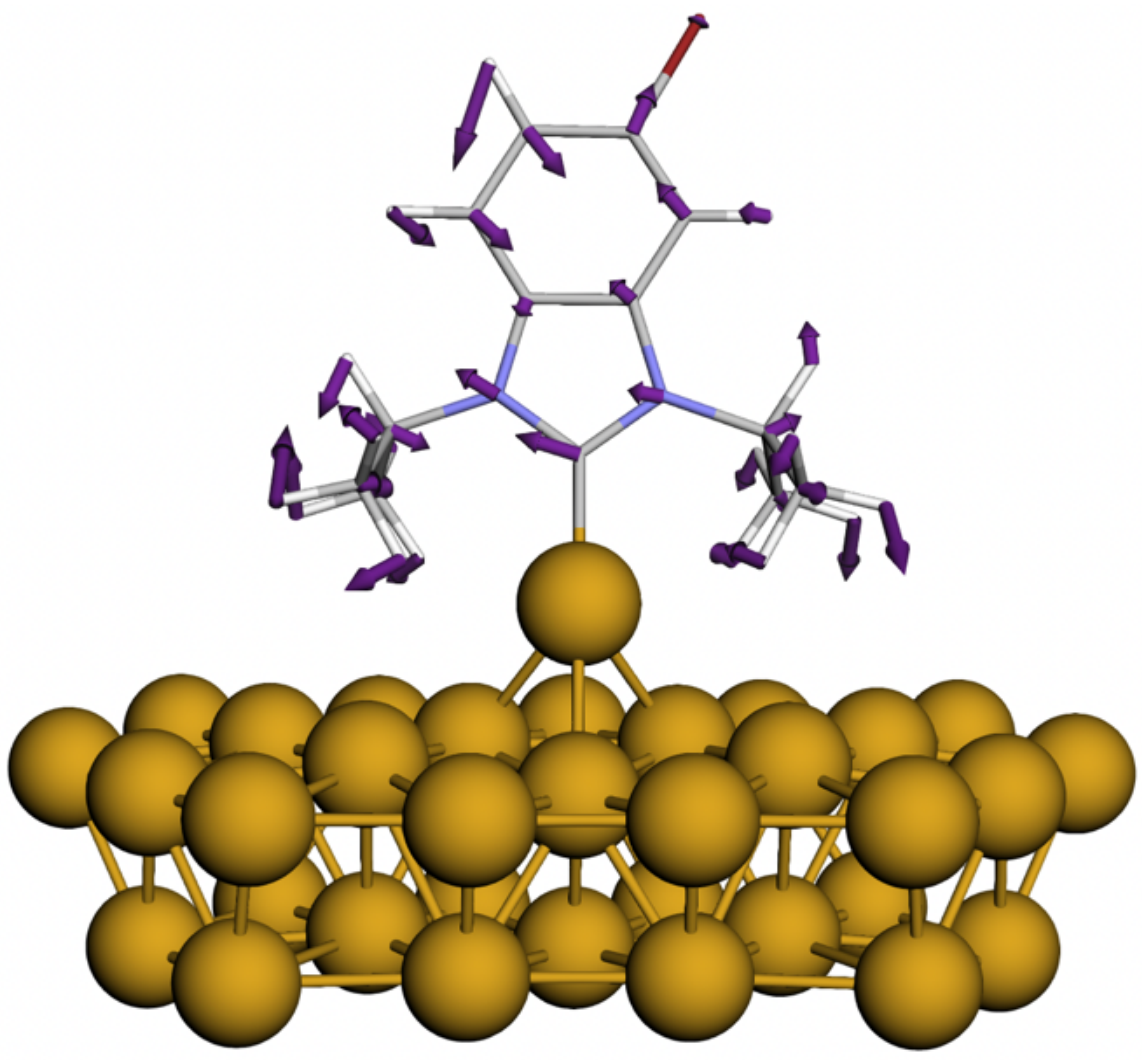

Figure S25 Vibration at 779 cm^{-1} for R = Br.

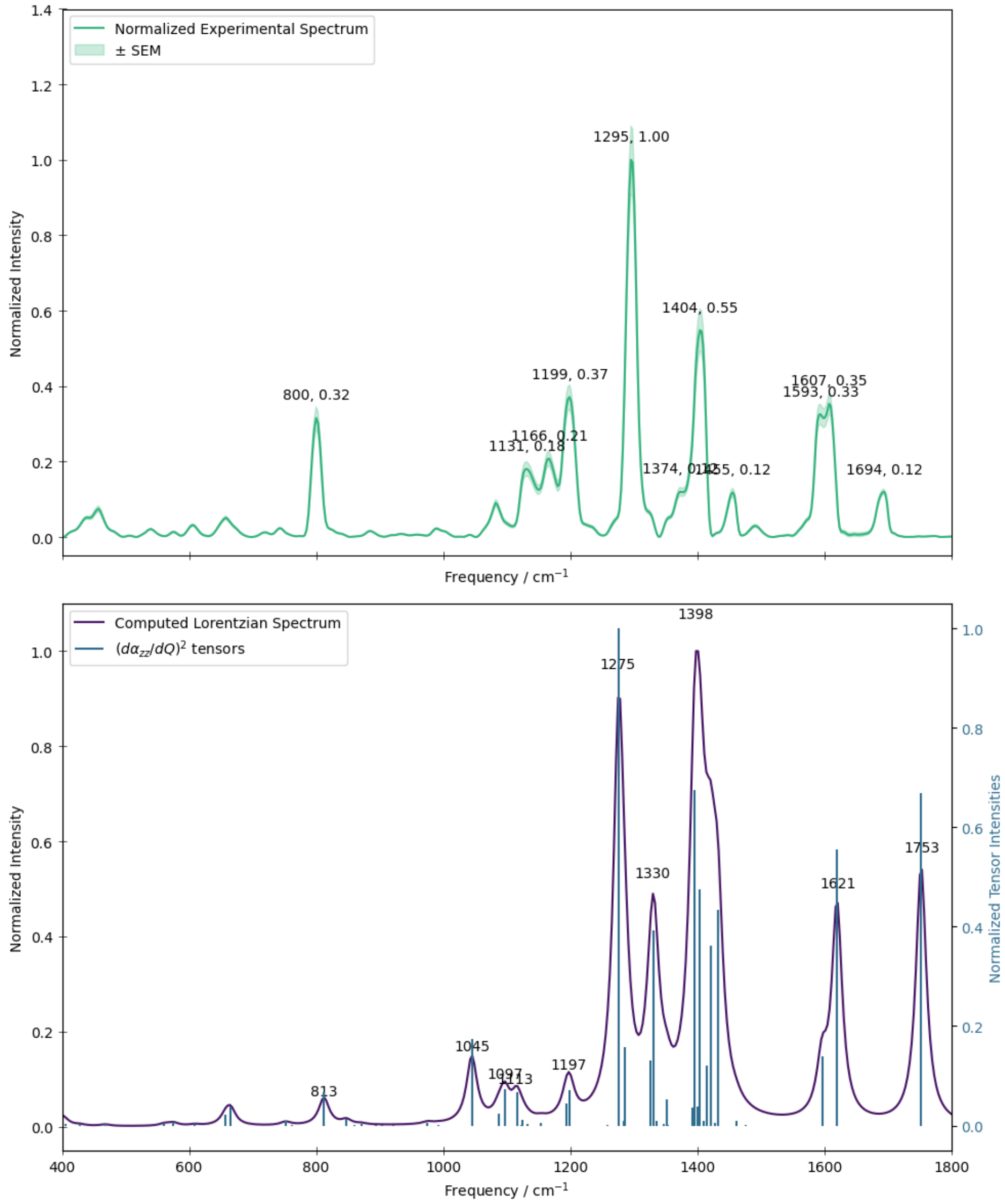

Figure S26 Experimental (top) versus predicted (bottom) SERS spectra for R = CHO.

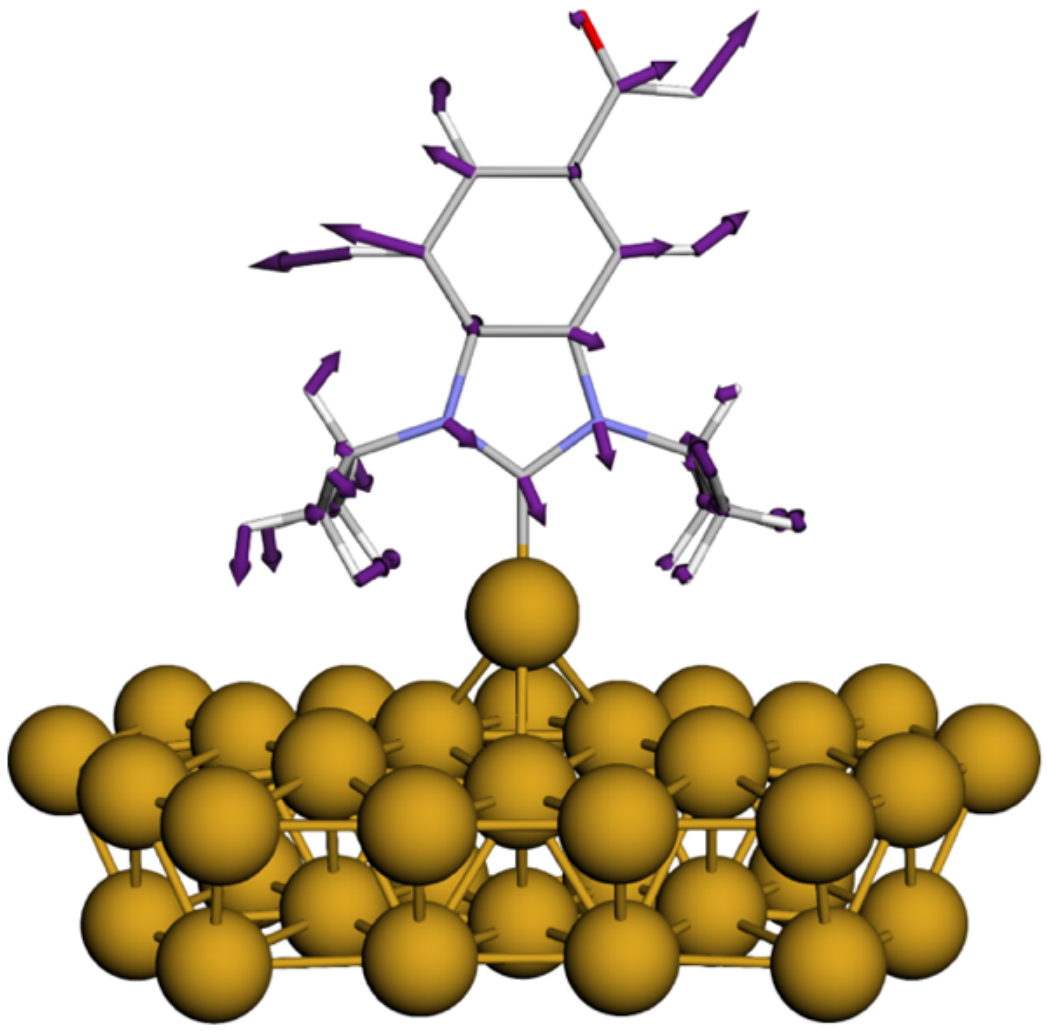

Figure S27 Vibration at 812 cm^{-1} for R = CHO.

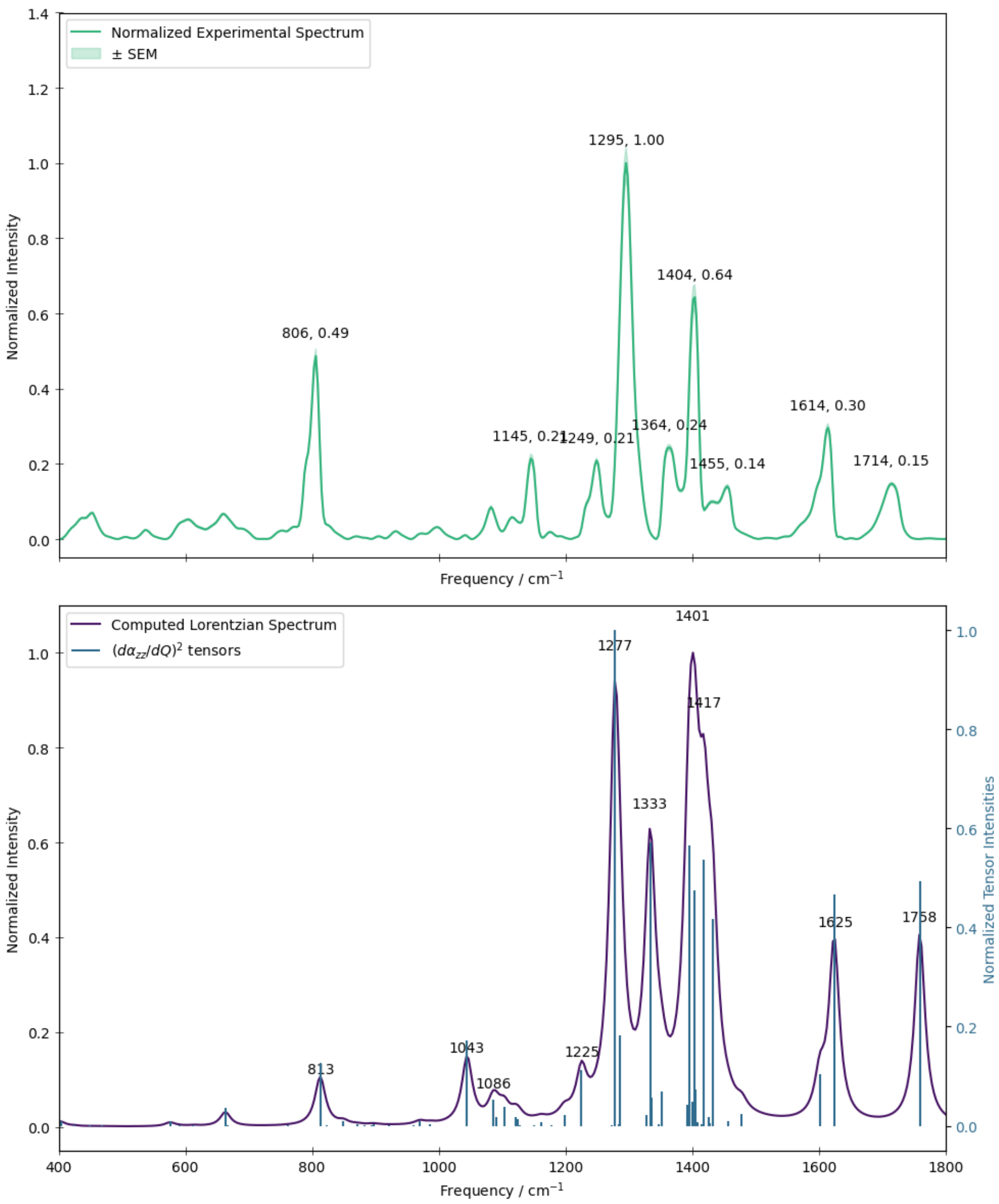

Figure S28 Experimental (top) versus predicted (bottom) SERS spectra for R = CO_2Me.

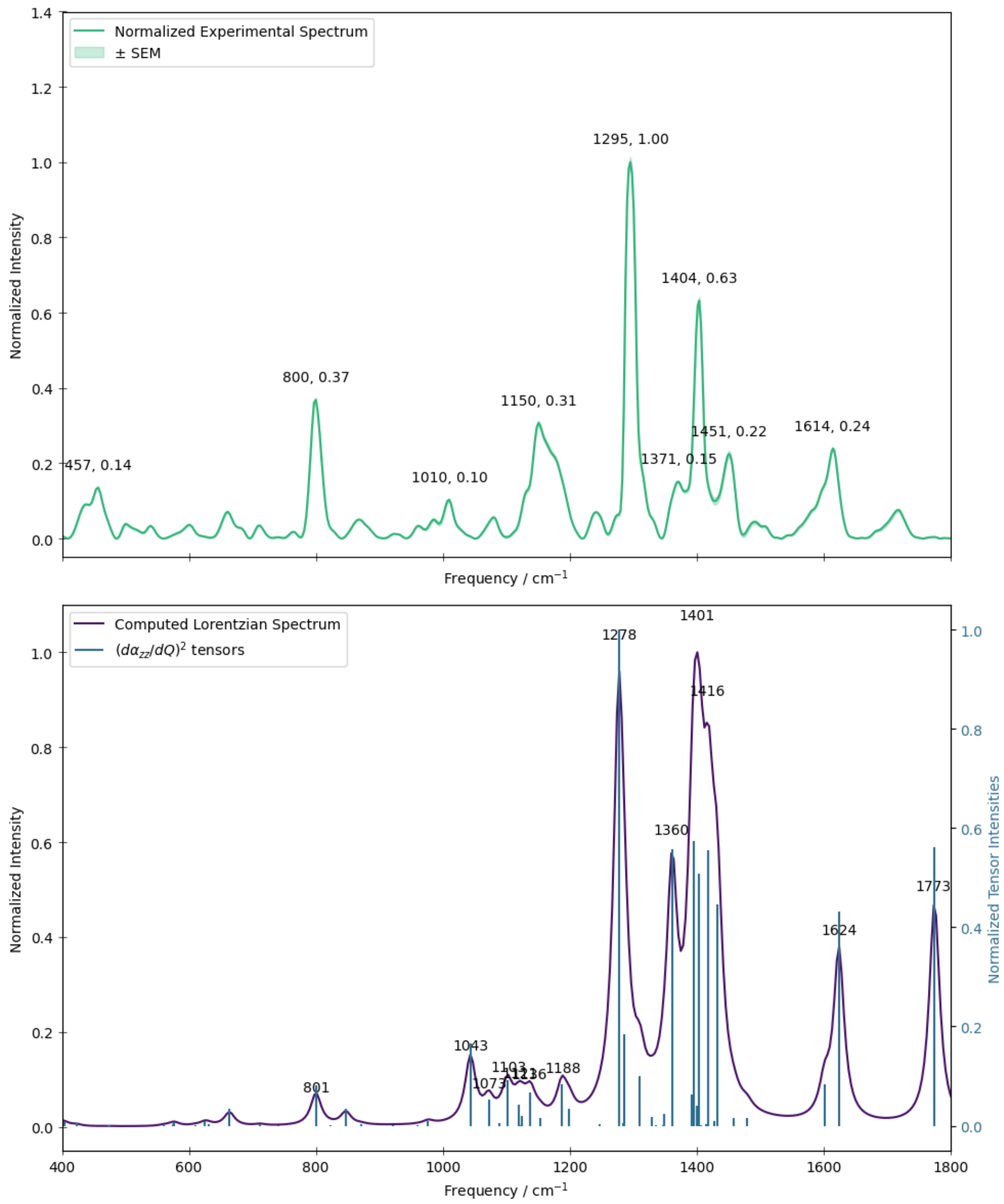

Figure S29 Experimental (top) versus predicted (bottom) SERS spectra for R = CO_2H.

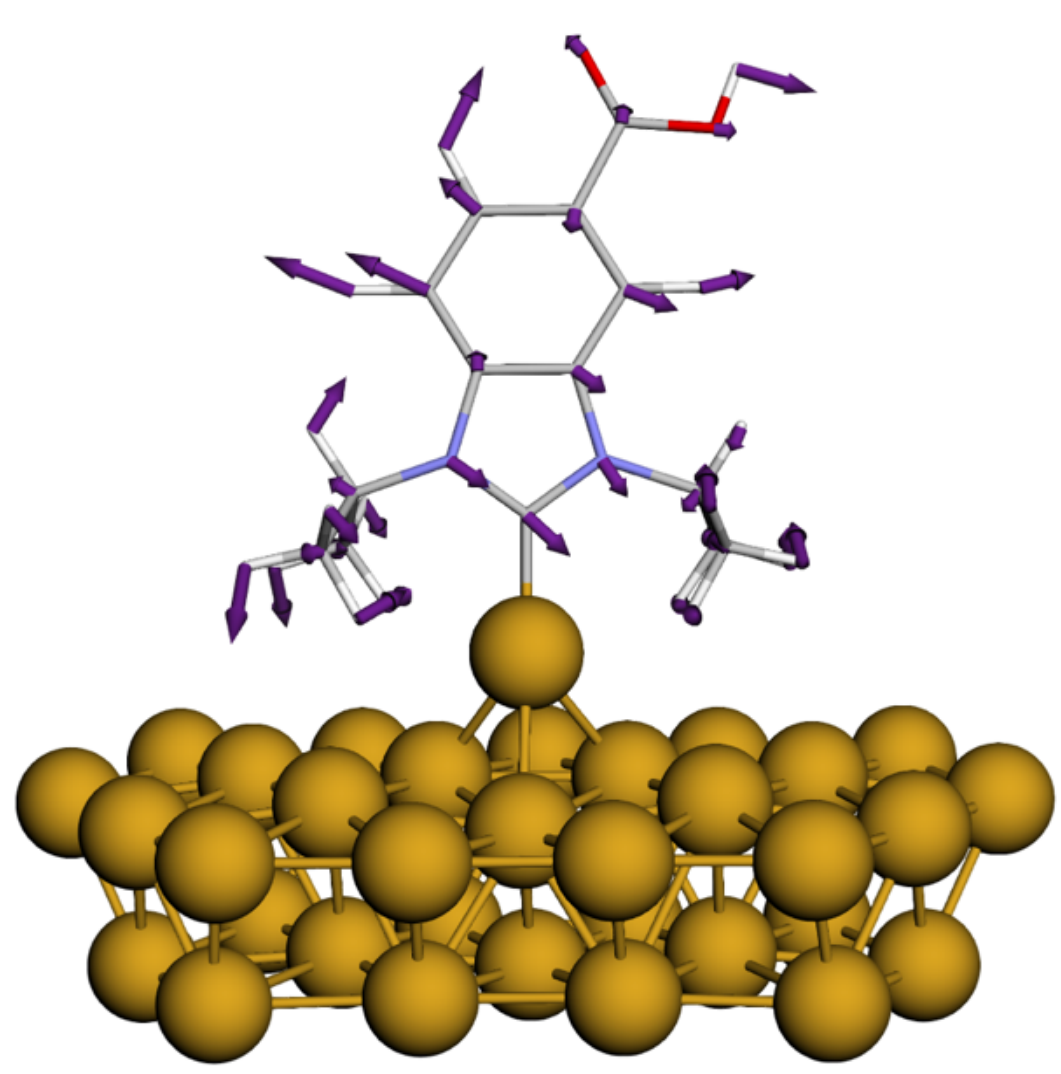

Figure S30 Vibration at 800 cm^{-1} for R = CO_2H.

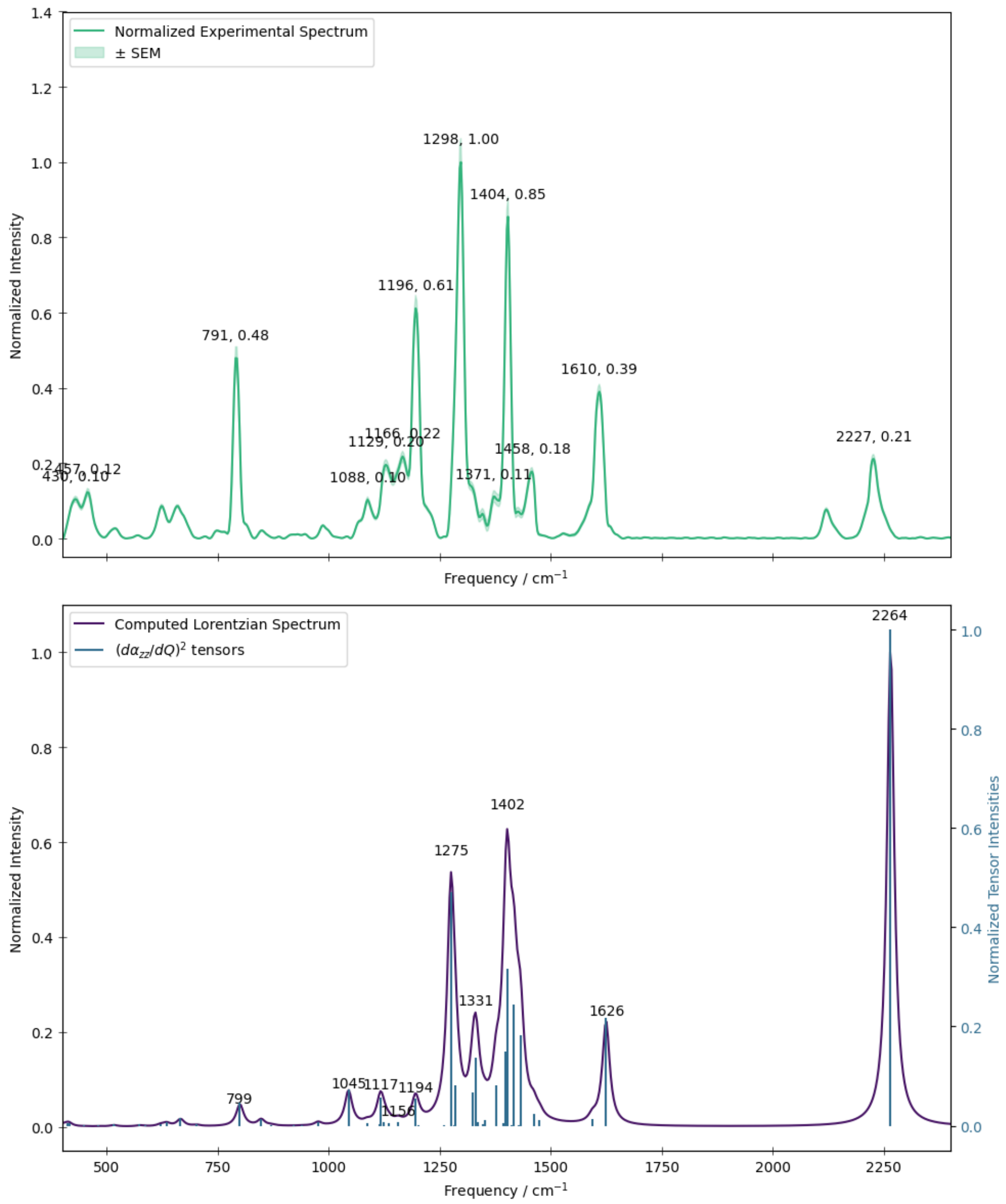

Figure S31 Experimental (top) versus predicted (bottom) SERS spectra for R = CN.

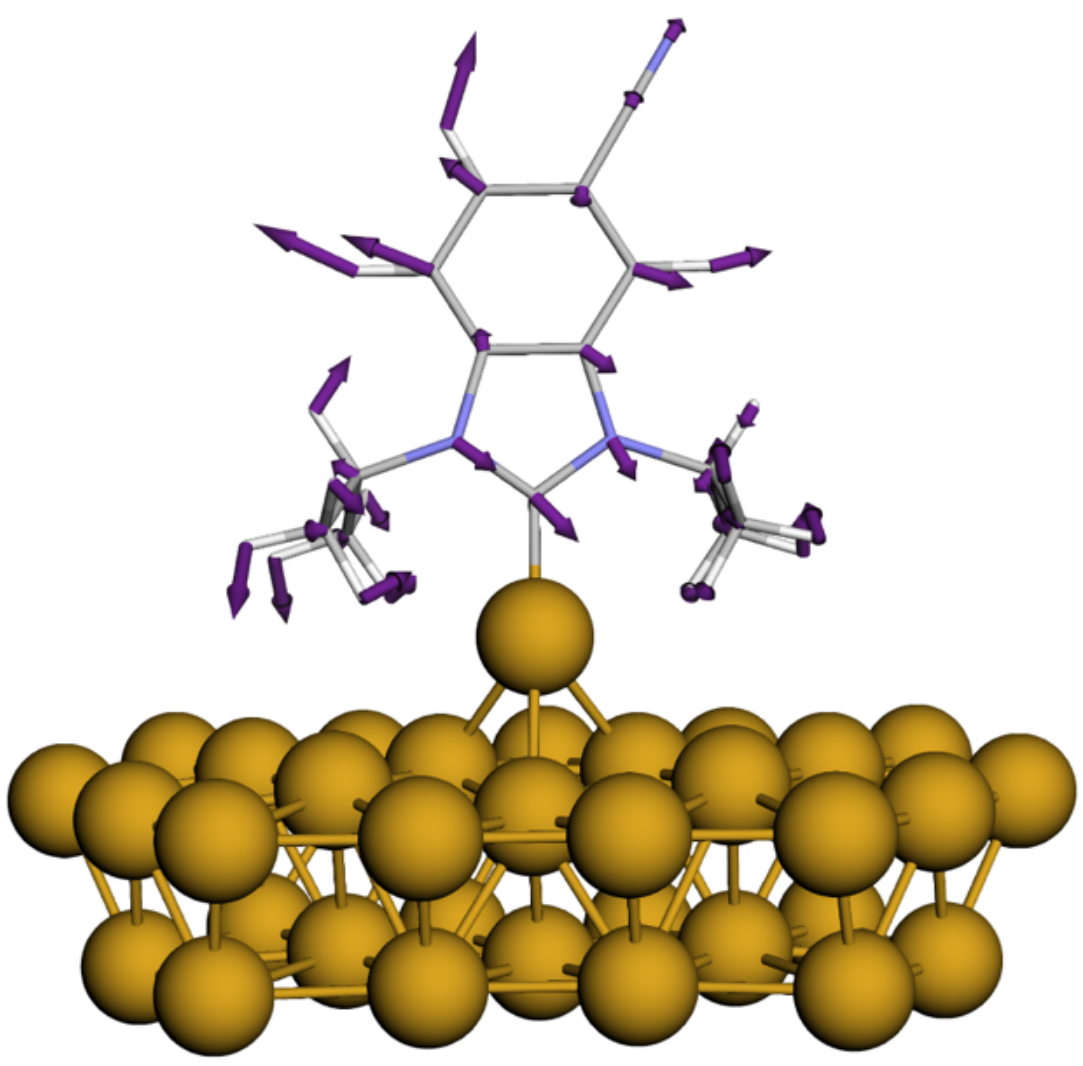

Figure S32 Vibration at 801 cm^{-1} for R = CN.

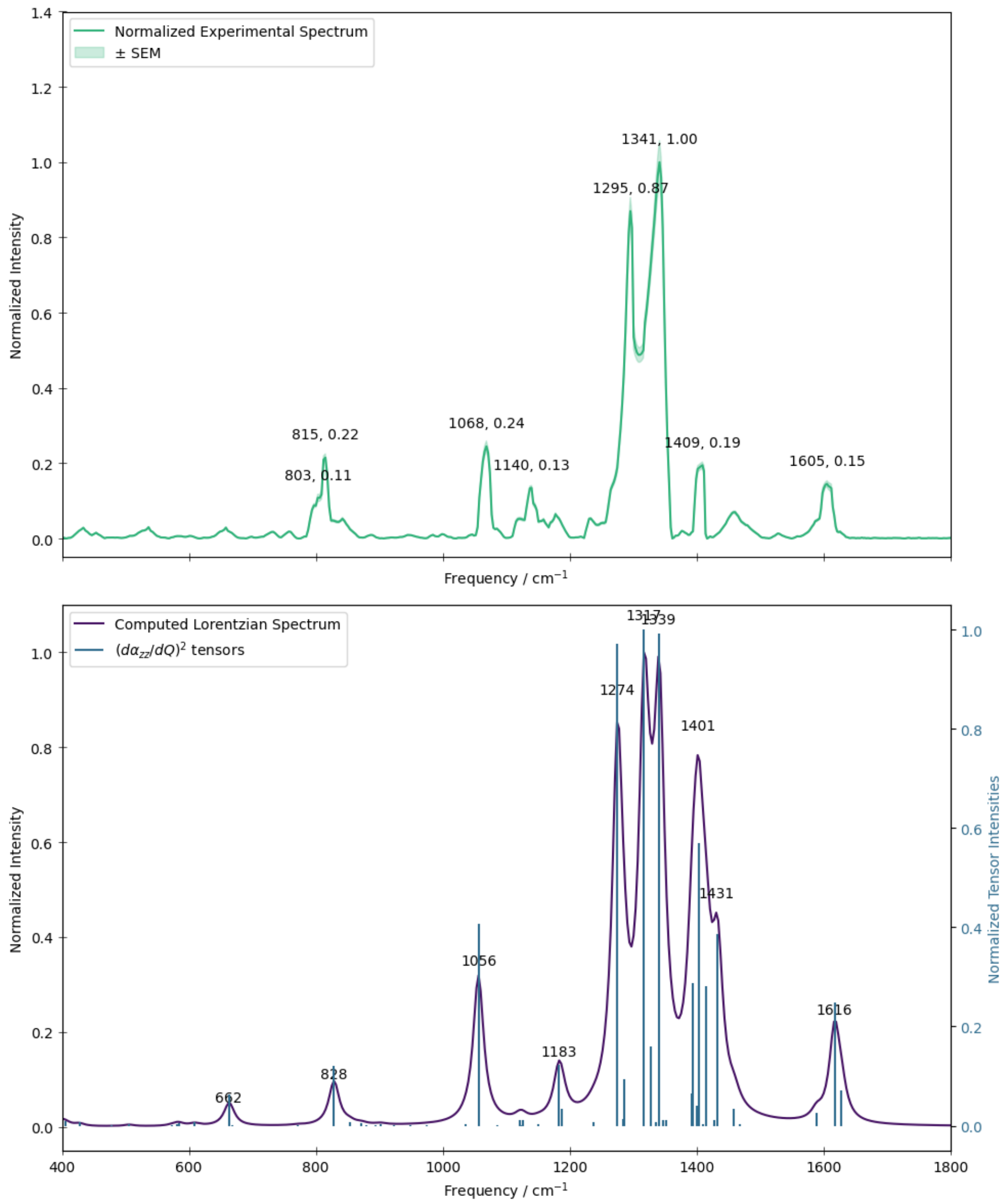

Figure S33 Experimental (top) versus predicted (bottom) SERS spectra for R = NO_2.

S6 Proton Coupled ^{13}C Spectra

All proton coupled ^{13}C spectra were recorded in DMSO-d^6 as solvents have been shown to influence the $^1J_{C\text{–}H}$ value.[26] The desired carbon environment is a diagnostic doublet of triplets. Additionally, the signal was assigned using the respective HSQC spectra (data not shown). The full stack is shown below.

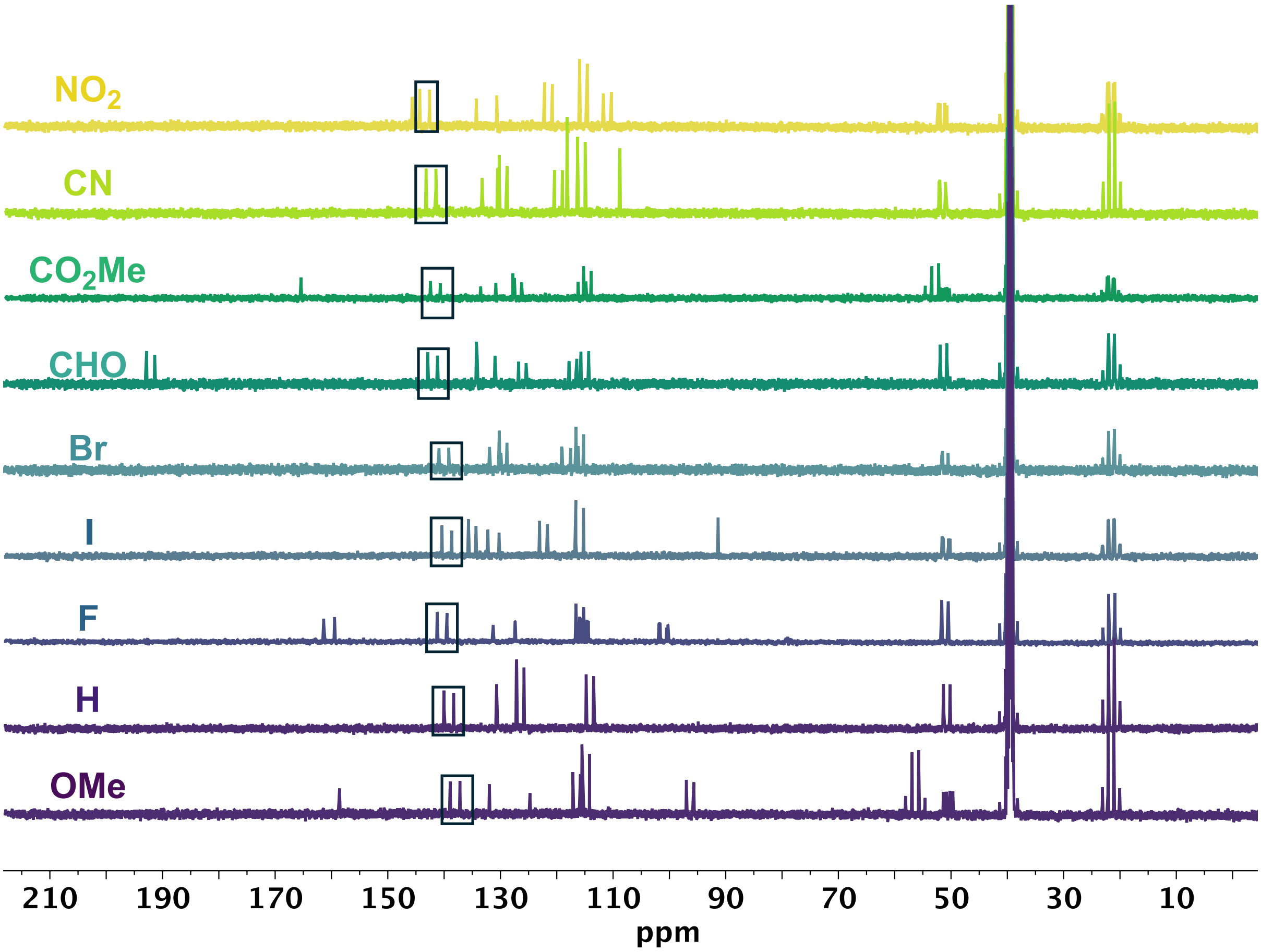

Figure S34 Full ^1H-coupled ^{13}C NMR with the relevant signals used to extract $^1J_{C\text{–}H}$ value between C-2 atom and the acidic proton labelled using a box.

Table S2 $^1J_{C\text{–}H}$ used to estimate the σ-donating properties of different carbenes compared to various σ values.

R	σ_p	σ_m	σ_{p-}	σ_{p+}	$^1J_{C\text{–}H}$ / Hz
OMe	-0.27	0.12	-0.26	-0.78	217.7
H	0.00	0.00	0.00	0.00	217.9
F	0.06	0.34	-0.03	-0.07	218.4
I	0.18	0.35	0.27	0.14	218.2
Br	0.23	0.39	0.25	0.15	218.7
CHO	0.42	0.35	1.03	0.73	218.8
CO_2Me	0.45	0.37	0.75	0.49	218.5
CN	0.66	0.56	1.00	0.66	219.6
NO_2	0.81	0.71	1.27	0.79	219.4

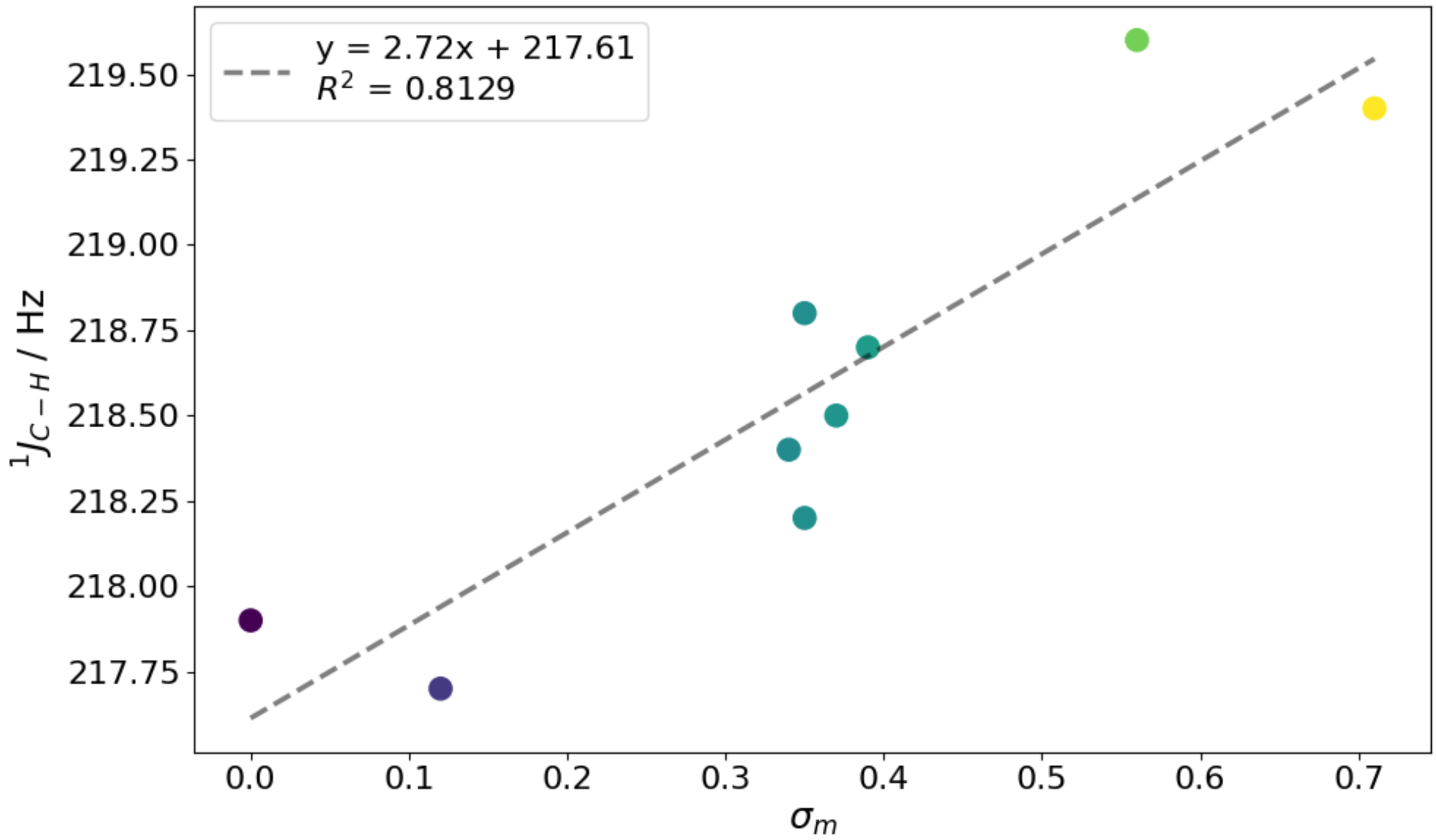

Figure S35 $^1J_{C\text{–}H}$ between C-2 atom and the acidic proton versus σ_m.

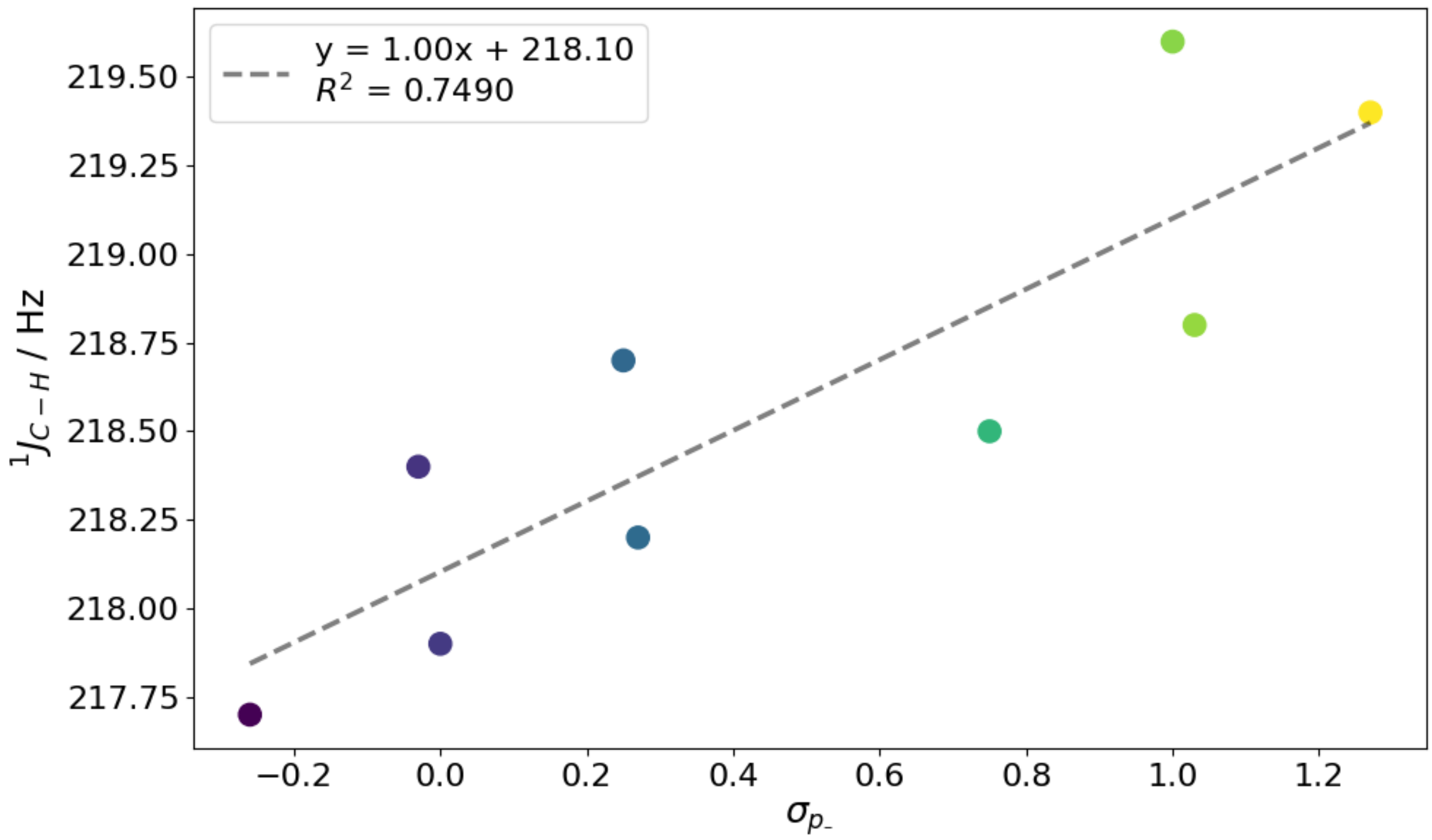

Figure S36 $^1J_{C\text{–}H}$ between C-2 atom and the acidic proton versus $\sigma_{p\text{–}}$.

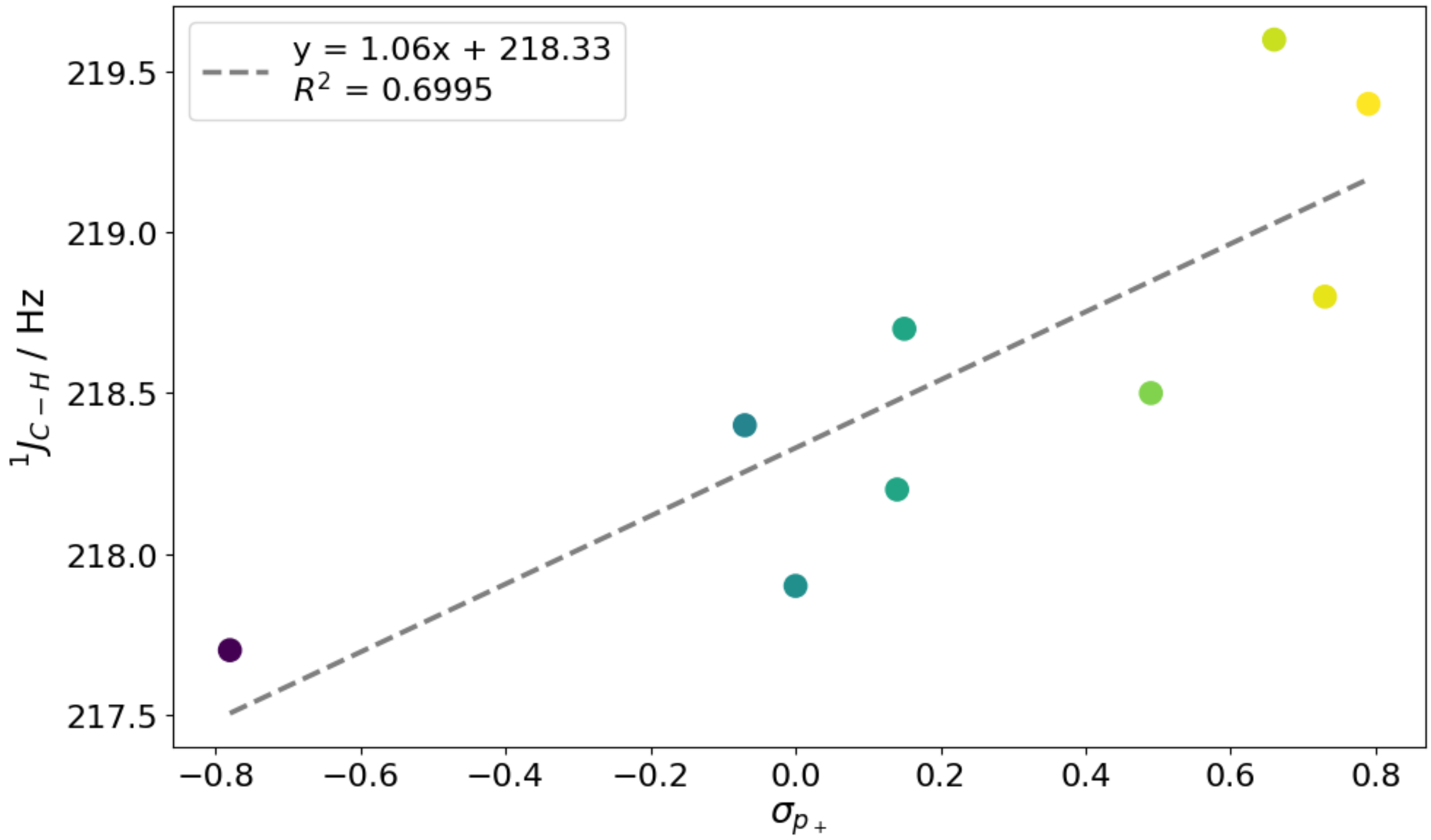

Figure S37 $^1J_{C\text{–}H}$ between C-2 atom and the acidic proton versus σ_{p+}.

S7 Frontier Molecular Orbitals Analysis

See section S3.2 for computational details. As mentioned in the paper, for all derivatives the HOMO was the carbene lone pair except for R = OMe and NH_2. The HOMO for these two derivatives is shown below.

Table S3 Orbital energies of different carbenes compared to various σ values.

R	σ_p	σ_m	σ_{p-}	σ_{p+}	Carbene Lone Pair / eV	LUMO / eV
NH_2	-0.66	-0.16	-0.15	-1.30	-5.96	-0.34
OMe	-0.27	0.12	-0.26	-0.78	-6.04	-0.47
H	0.00	0.00	0.00	0.00	-6.14	-0.51
F	0.06	0.34	-0.03	-0.07	-6.25	-0.71
I	0.18	0.35	0.27	0.14	-6.32	-0.84
Br	0.23	0.39	0.25	0.15	-6.28	-0.82
CHO	0.42	0.35	1.03	0.73	-6.46	-1.64
CO_2Me	0.45	0.37	0.75	0.49	-6.32	-1.21
CO_2H	0.45	0.37	0.77	0.42	-6.37	-1.32
CN	0.66	0.56	1.00	0.66	-6.59	-1.41
NO_2	0.81	0.71	1.27	0.79	-6.62	-2.28

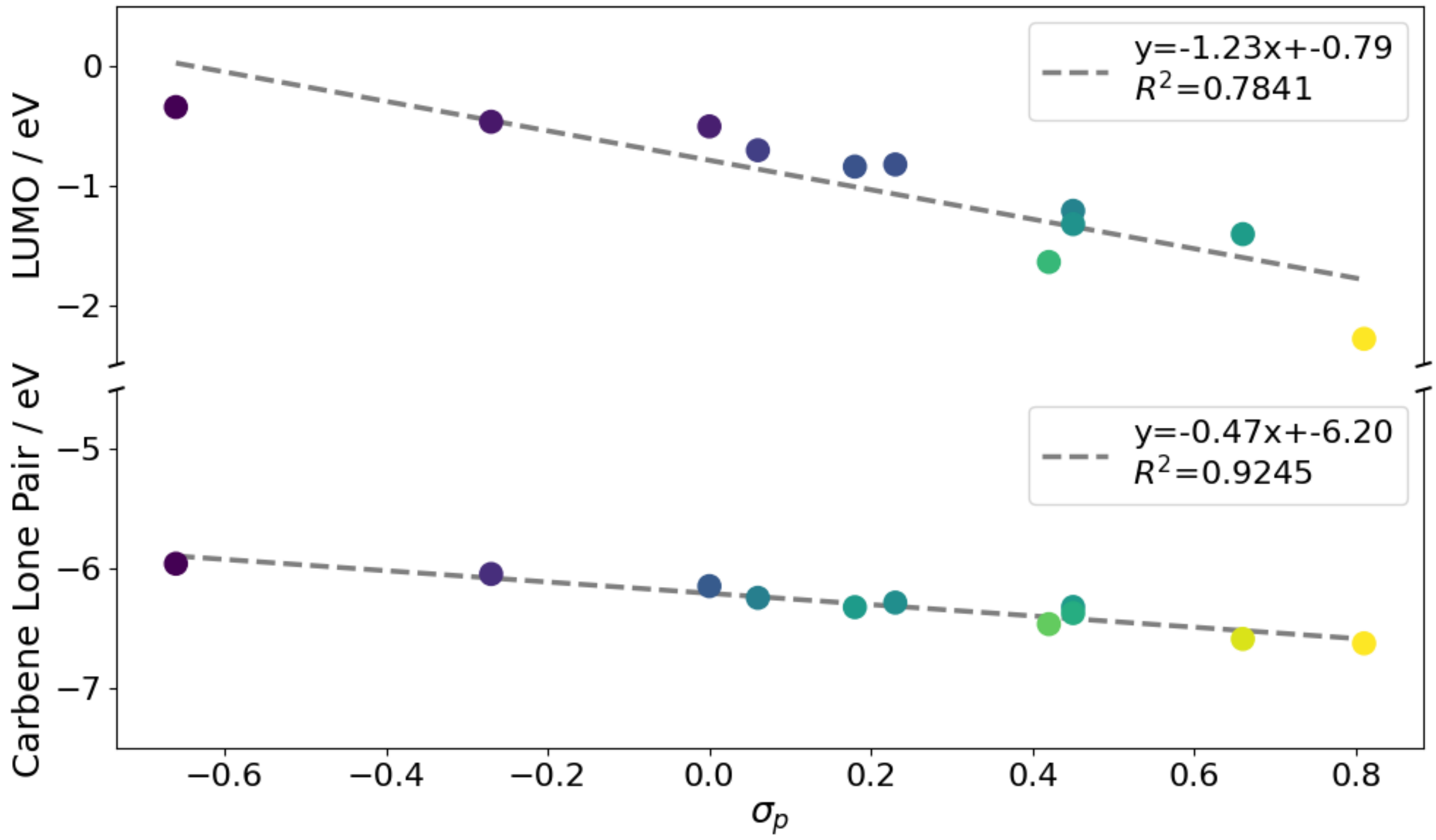

Figure S38 Carbene lone pair and LUMO energy values for the carbene ligands as a function of σ_p.

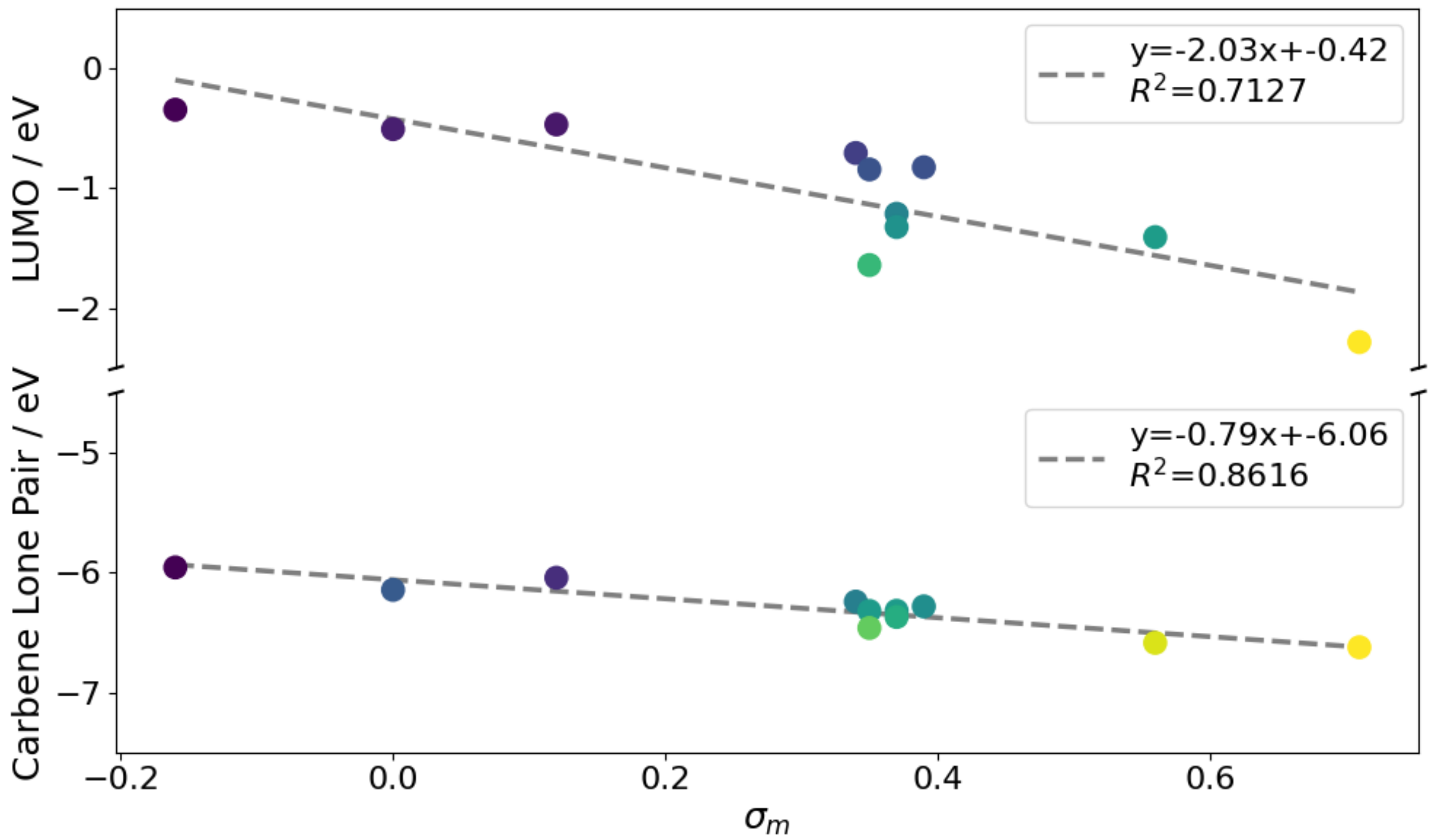

Figure S39 Carbene lone pair and LUMO energy values for the carbene ligands as a function of σ_m.

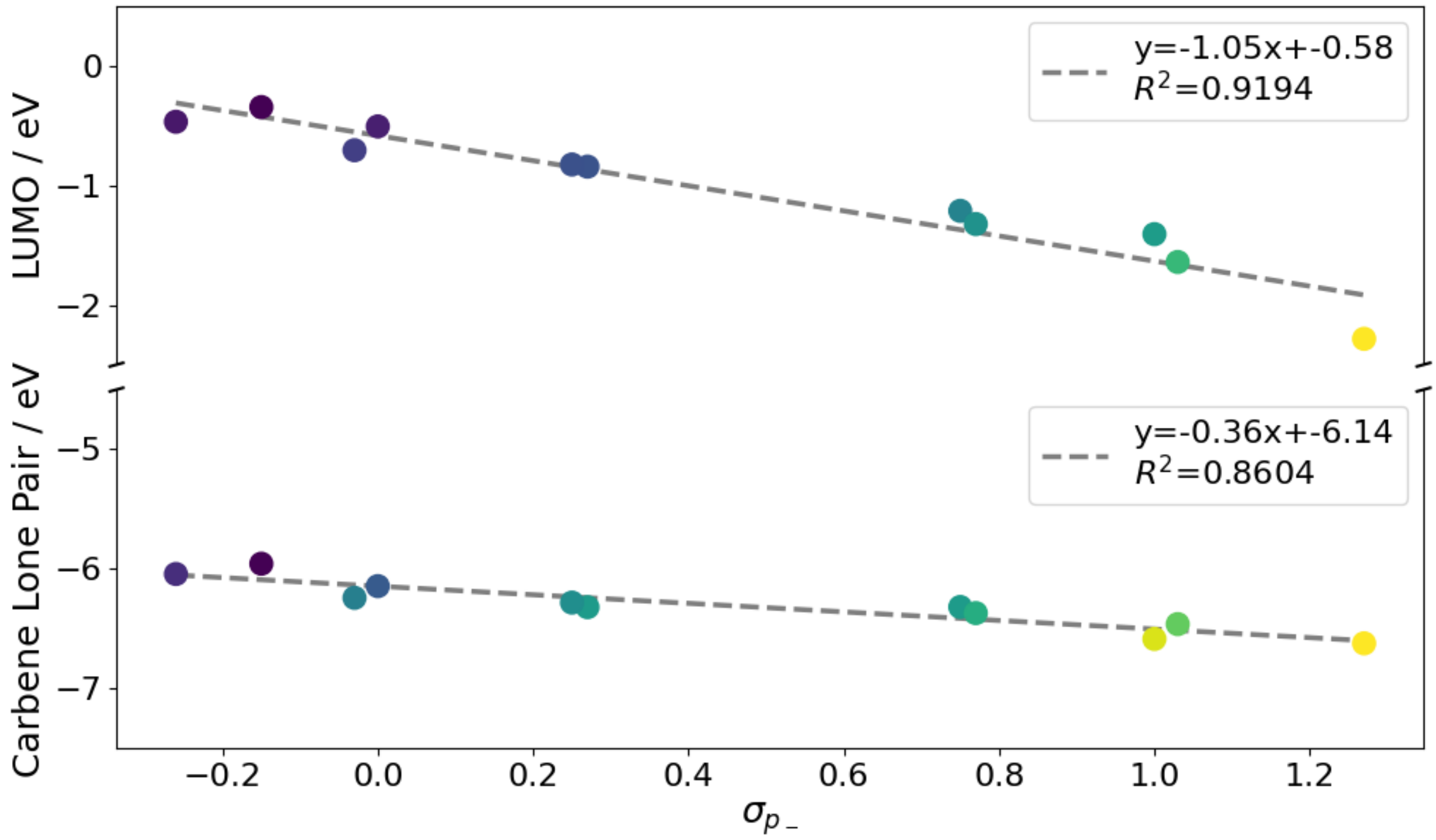

Figure S40 Carbene lone pair and LUMO energy values for the carbene ligands as a function of σ_{p-}.

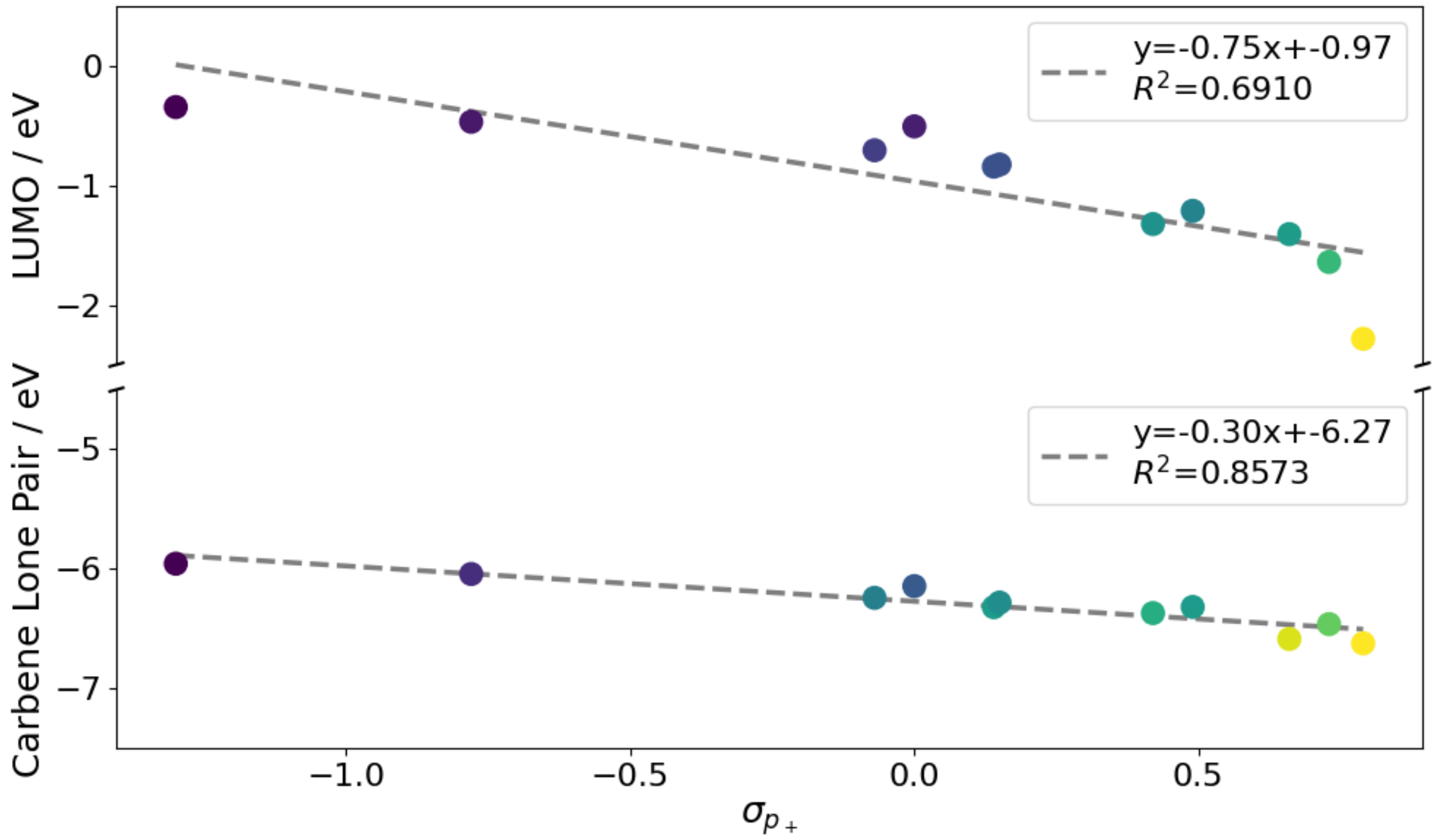

Figure S41 Carbene lone pair and LUMO energy values for the carbene ligands as a function of σ_{p+}.

Note: The HOMO, LUMO and carbene lone pair depictions for R = OMe, H and NO_2 are given in figure 3d-i of the manuscript.

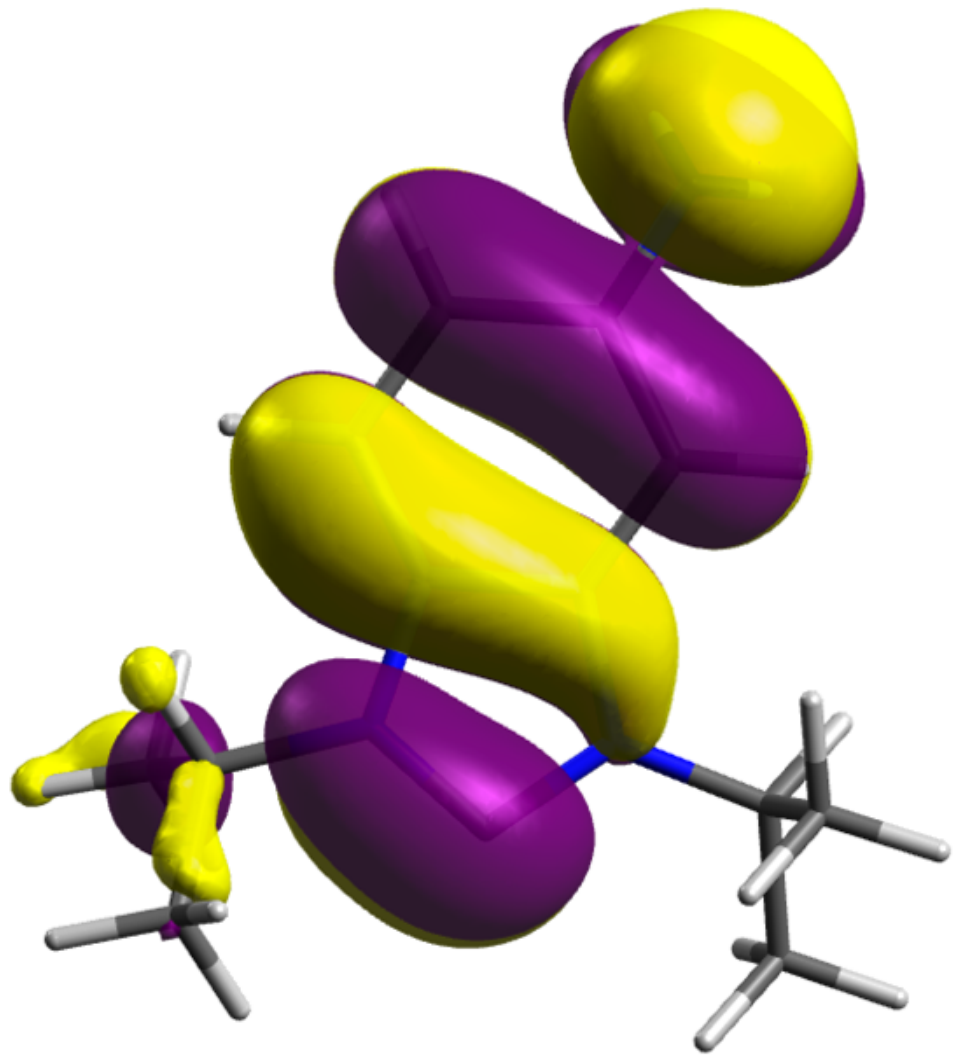

Figure S42 Depiction of the HOMO for R = NH_2; Hydrogen atoms are depicted in white, carbon in grey and nitrogen in blue; isovalue = 0.015 e $bohr^{-3}$. Energy = -5.62 eV.

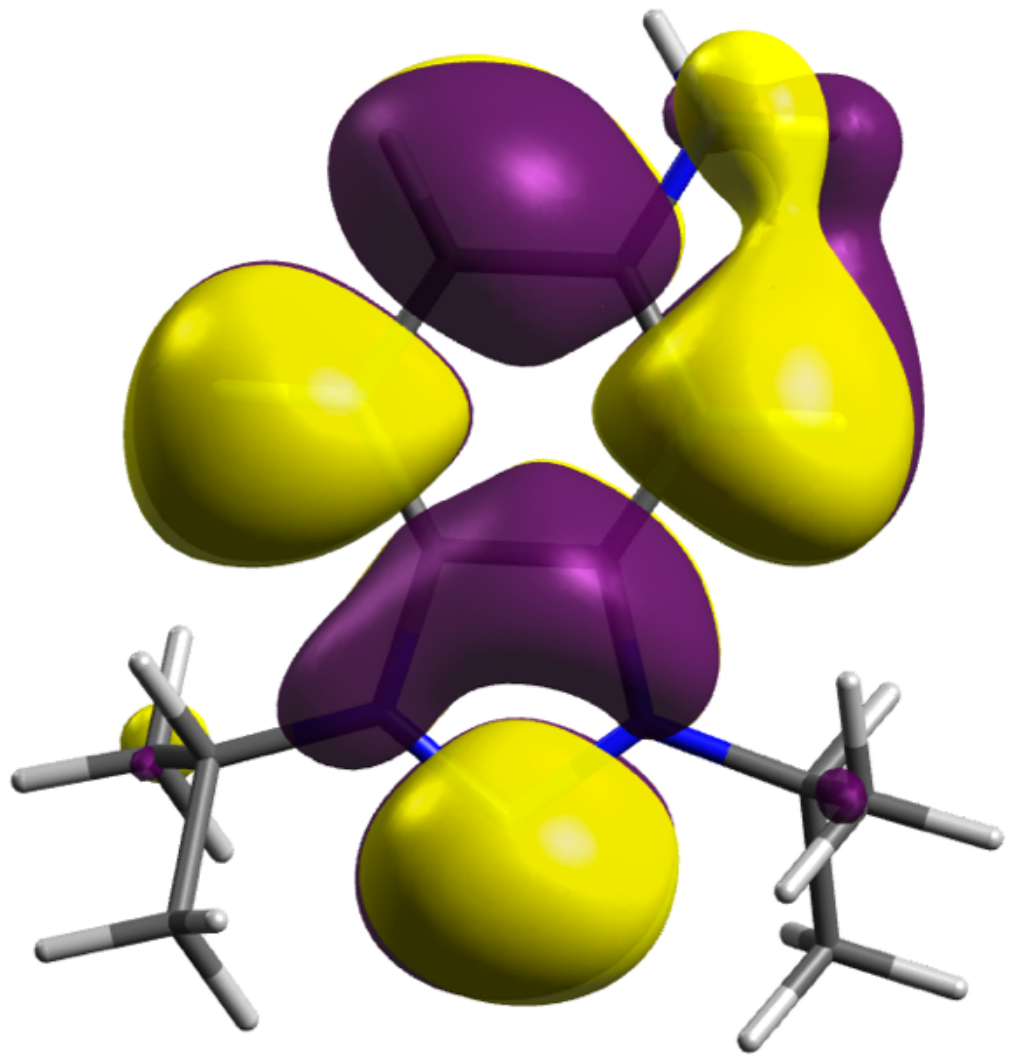

Figure S43 Depiction of the LUMO for R = NH_2; Hydrogen atoms are depicted in white, carbon in grey and nitrogen in blue; isovalue = 0.015 e $bohr^{-3}$.

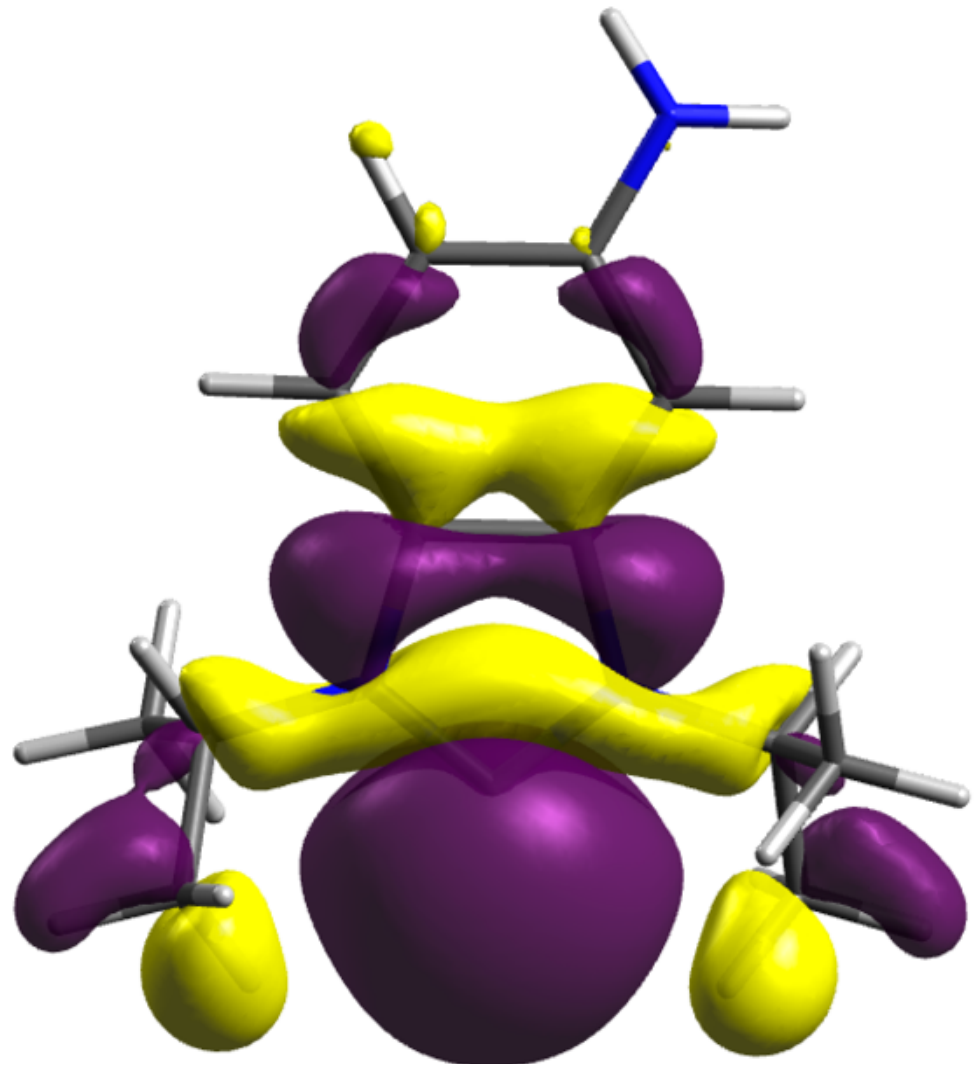

Figure S44 Depiction of the HOMO–1 (the carbene lone pair) for R = NH_2; Hydrogen atoms are depicted in white, carbon in grey and nitrogen in blue; isovalue = 0.015 e $bohr^{-3}$.

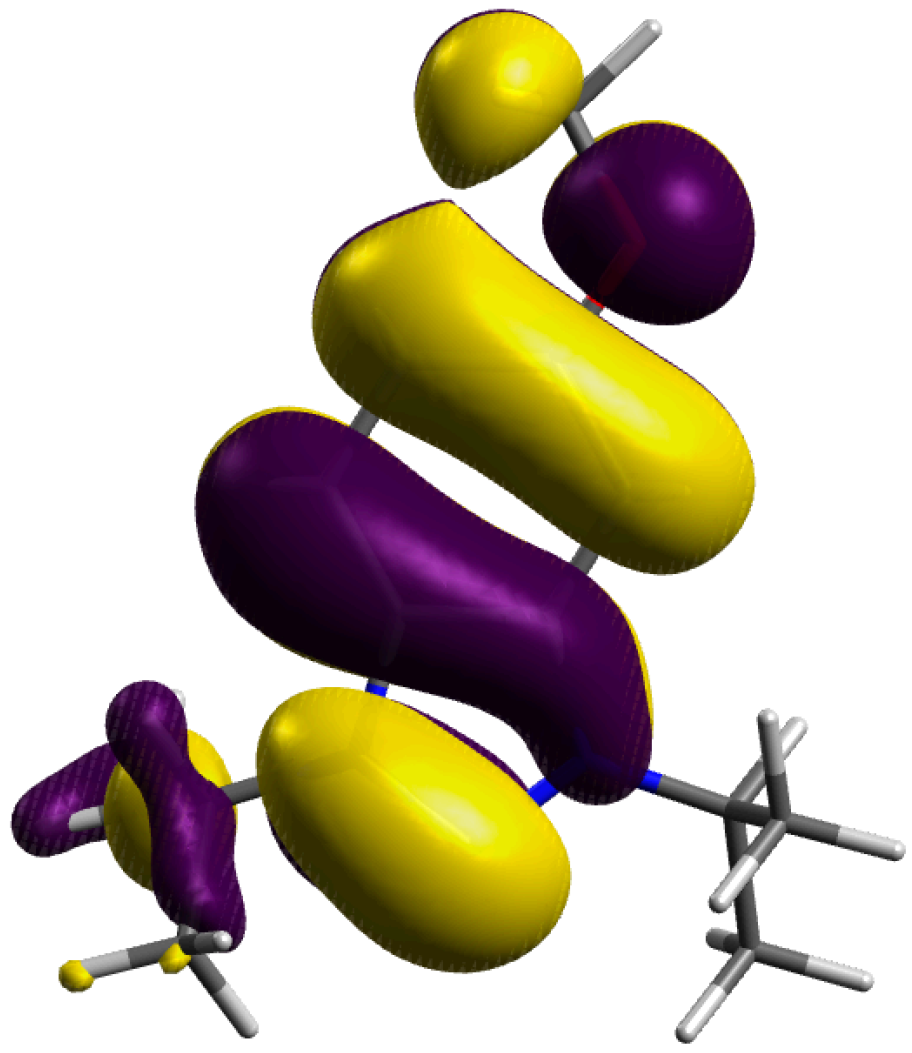

Figure S45 Depiction of the HOMO for R = OMe; Hydrogen atoms are depicted in white, carbon in grey, nitrogen in blue and oxygen in red; isovalue = 0.015 e $bohr^{-3}$.

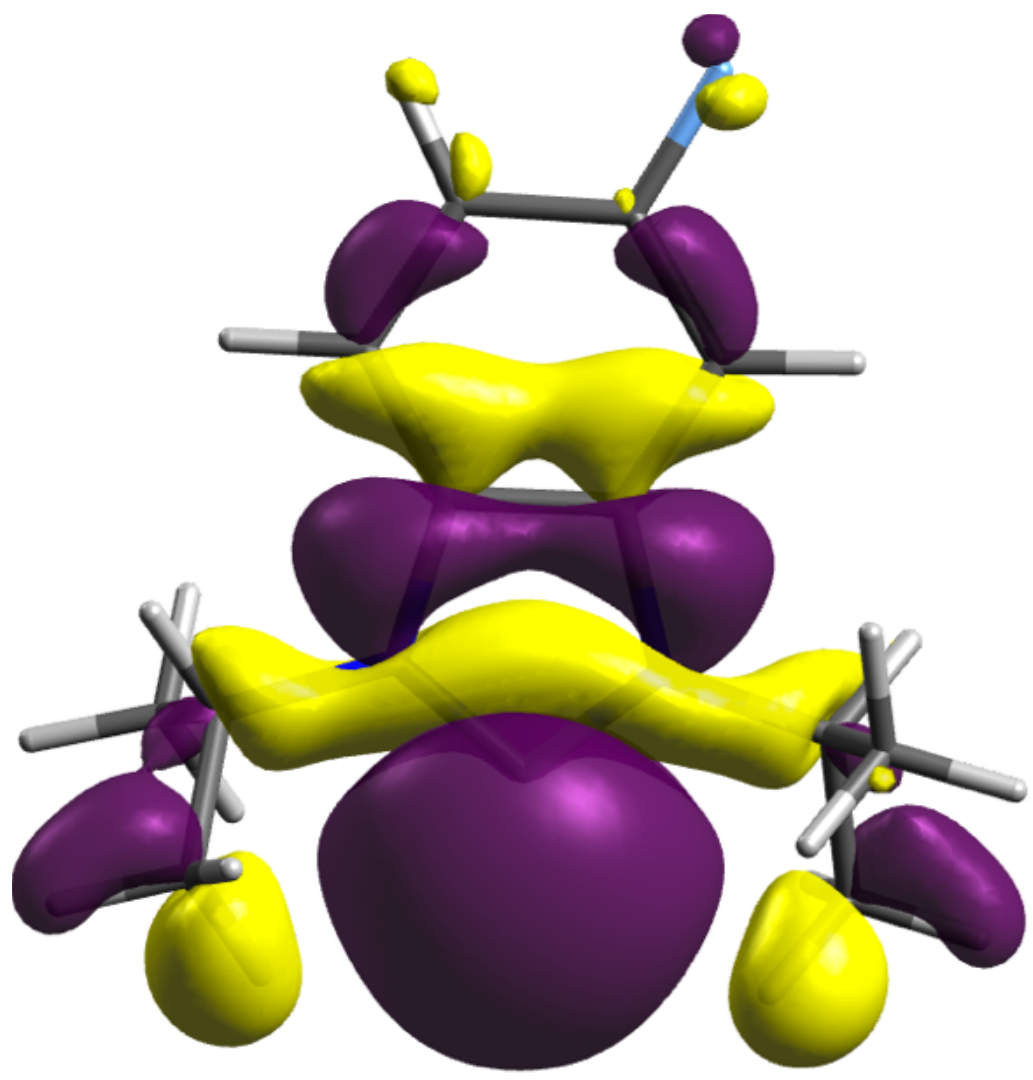

Figure S46 Depiction of the HOMO for R = F; Hydrogen atoms are depicted in white, carbon in grey, nitrogen in blue and fluorine in light blue; isovalue = 0.015 e $bohr^{-3}$.

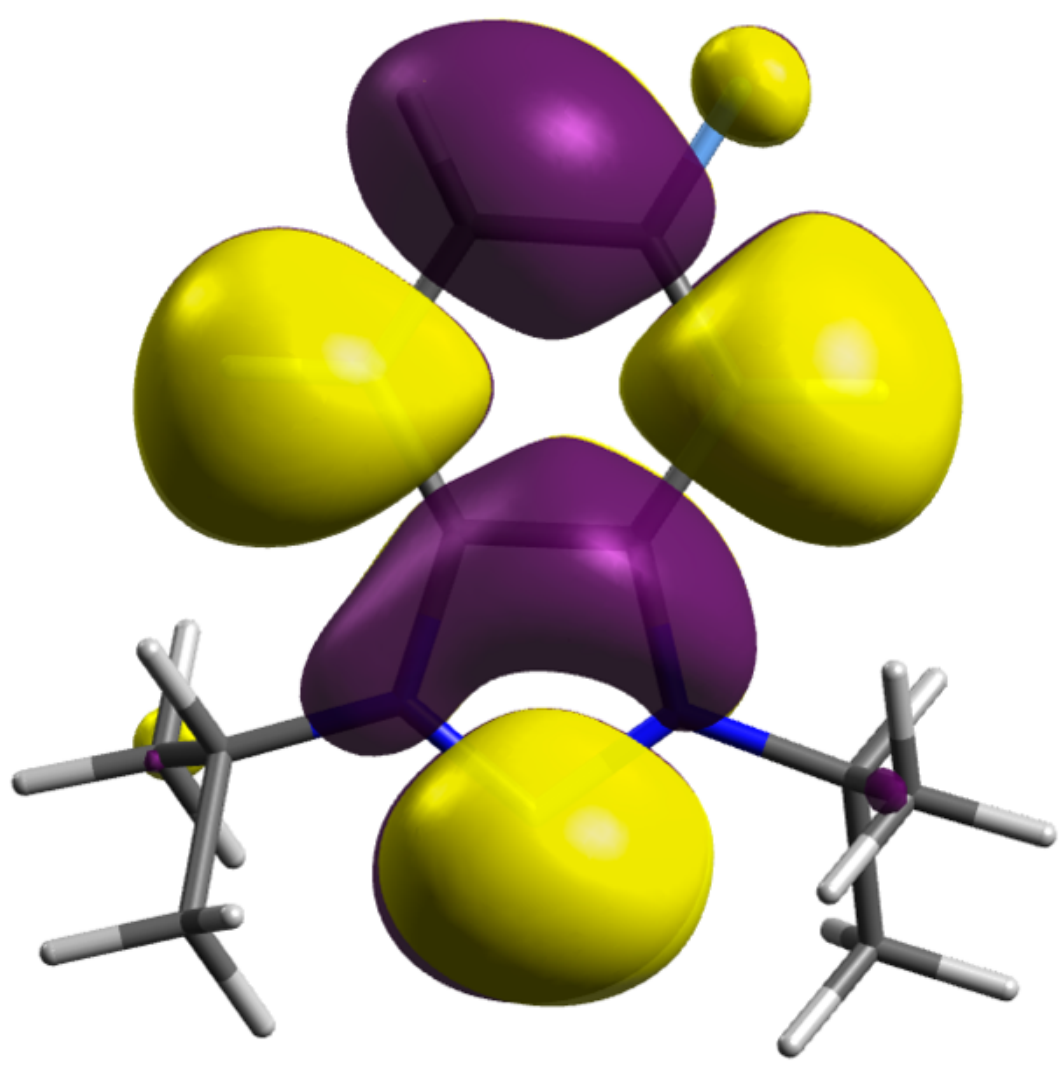

Figure S47 Depiction of the LUMO for R = F; Hydrogen atoms are depicted in white, carbon in grey, nitrogen in blue and fluorine in light blue; isovalue = 0.015 e $bohr^{-3}$.

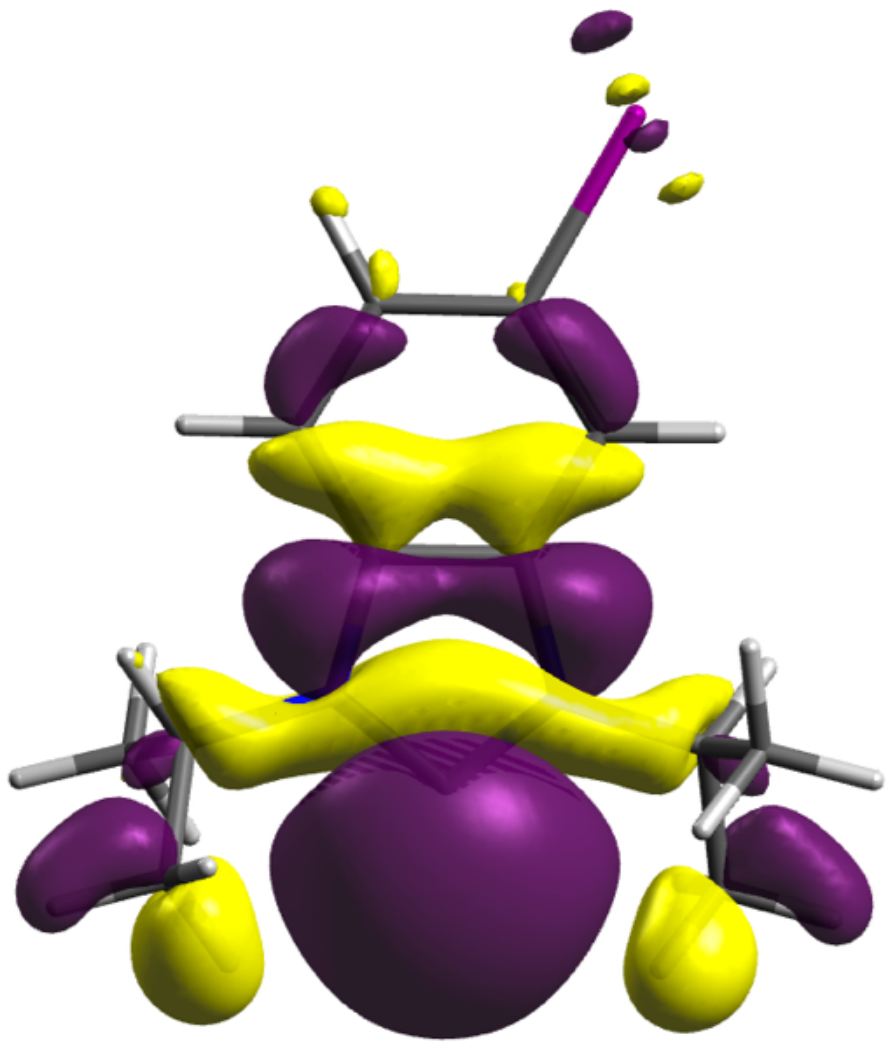

Figure S48 Depiction of the HOMO for R = I; Hydrogen atoms are depicted in white, carbon in grey, nitrogen in blue and iodine in magenta; isovalue = 0.015 e bohr^{-3}.

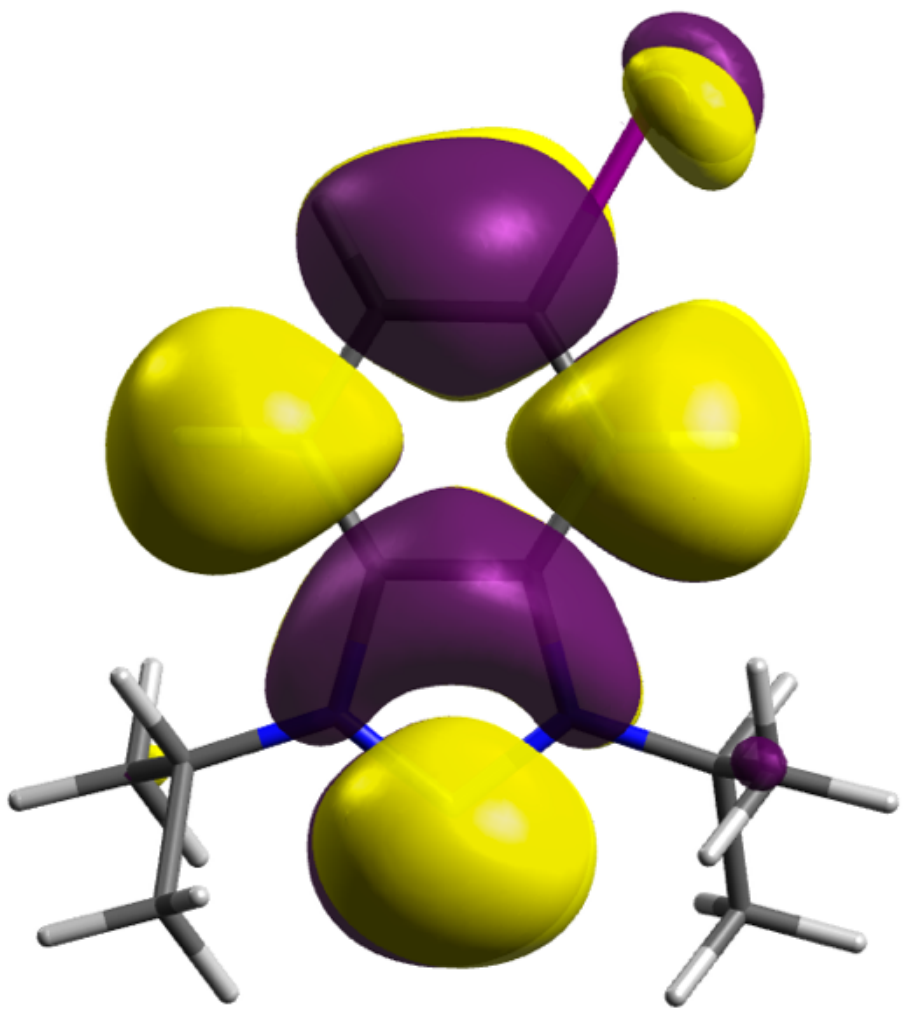

Figure S49 Depiction of the LUMO for R = I; Hydrogen atoms are depicted in white, carbon in grey, nitrogen in blue and iodine in magenta; isovalue = 0.015 e bohr^{-3}.

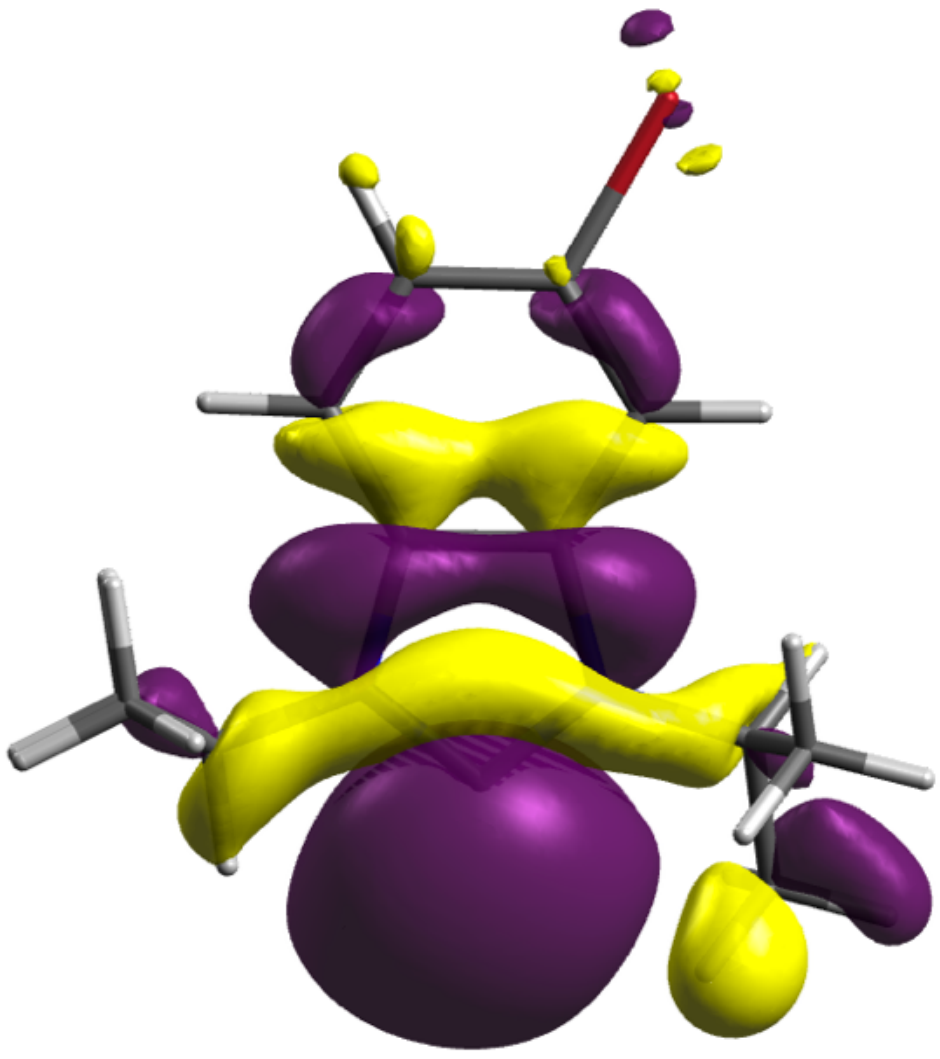

Figure S50 Depiction of the HOMO for R = Br; Hydrogen atoms are depicted in white, carbon in grey, nitrogen in blue and bromine in crismon; isovalue = 0.015 e $bohr^{-3}$.

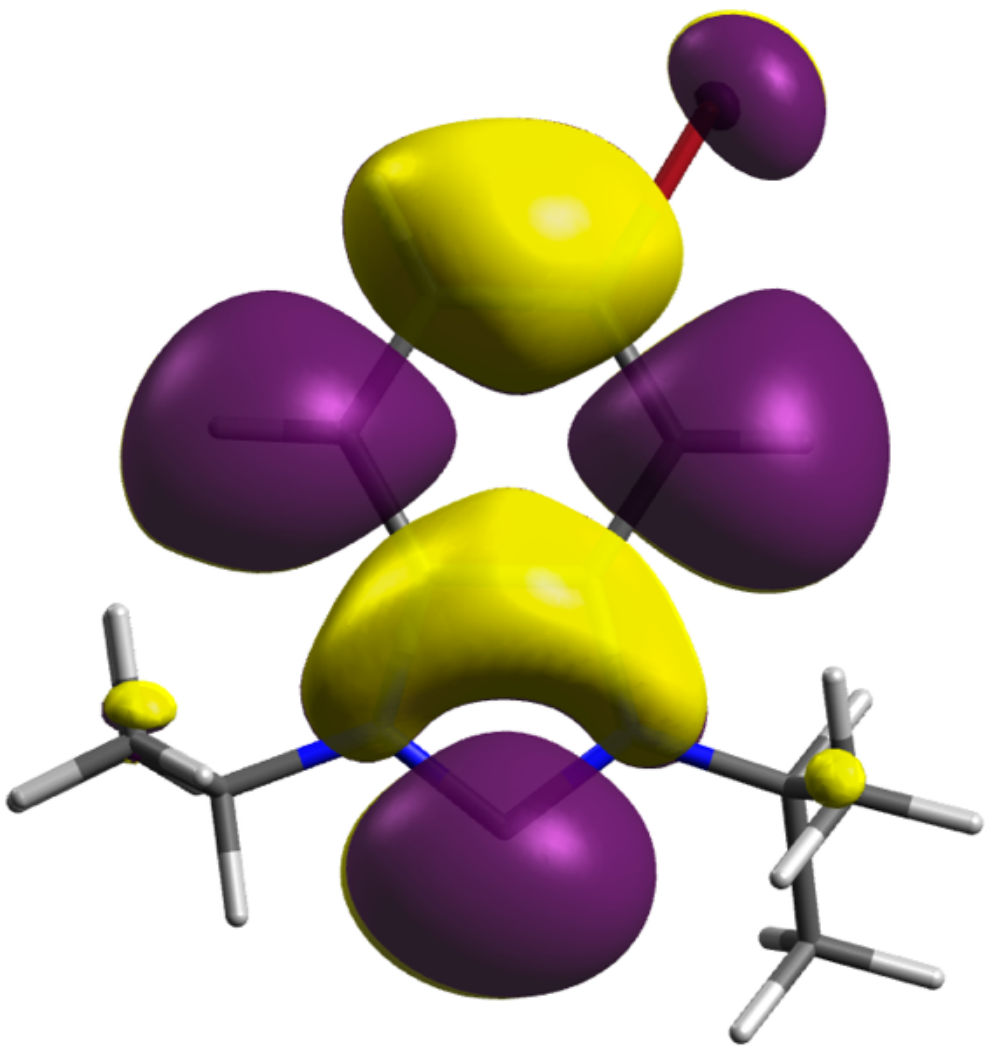

Figure S51 Depiction of the LUMO for R = Br; Hydrogen atoms are depicted in white, carbon in grey, nitrogen in blue and bromine in crismon; isovalue = 0.015 e $bohr^{-3}$.

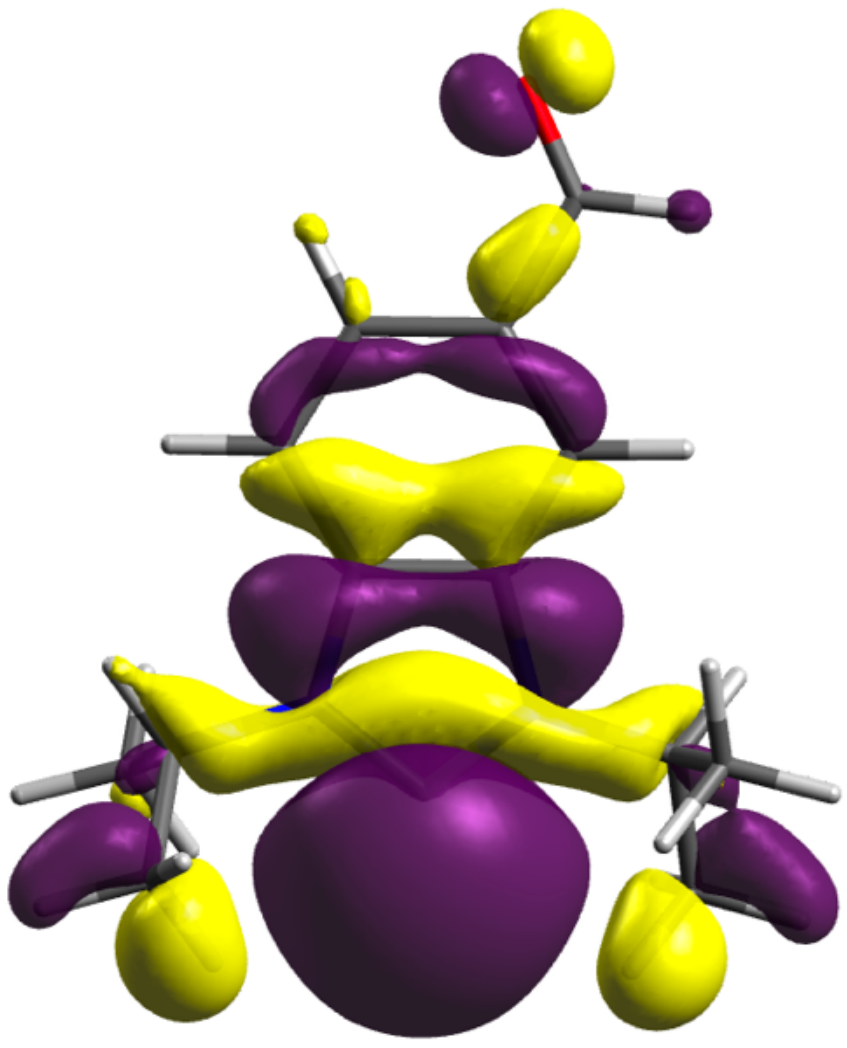

Figure S52 Depiction of the HOMO for R = CHO; Hydrogen atoms are depicted in white, carbon in grey, nitrogen in blue and oxygen in red; isovalue = 0.015 e bohr^{-3}.

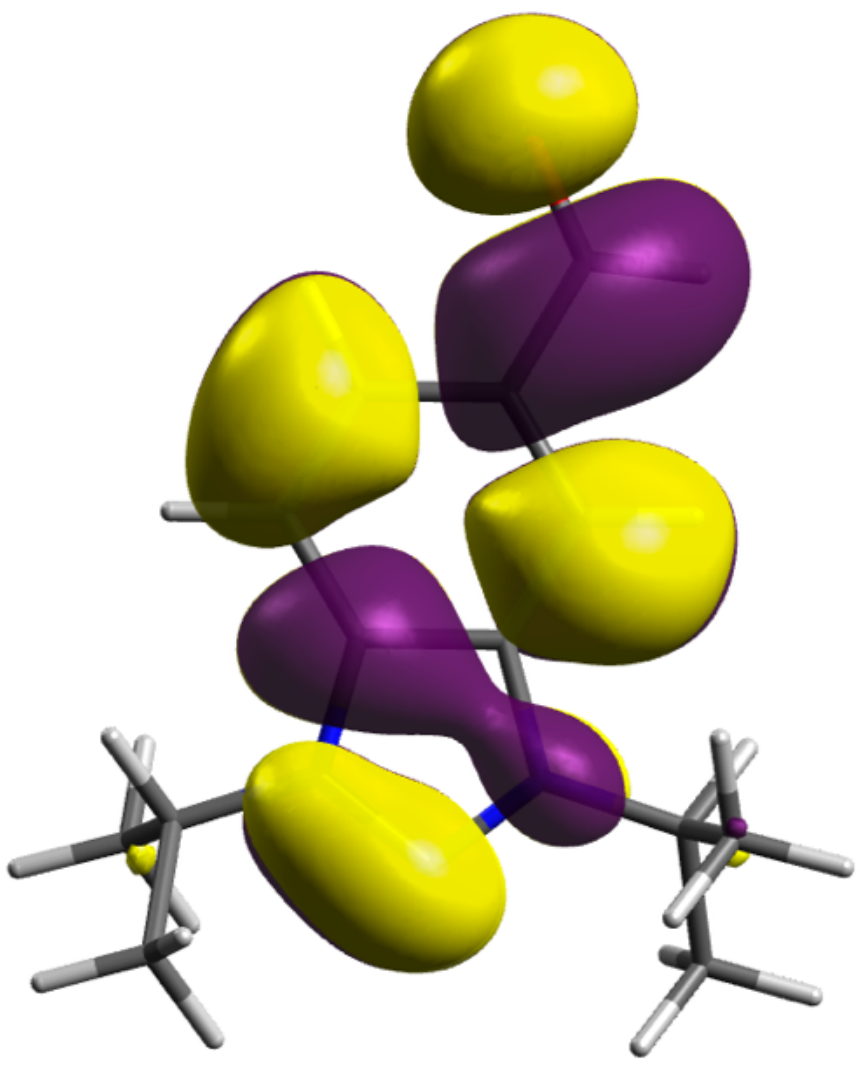

Figure S53 Depiction of the LUMO for R = CHO; Hydrogen atoms are depicted in white, carbon in grey, nitrogen in blue and oxygen in red; isovalue = 0.015 e bohr^{-3}.

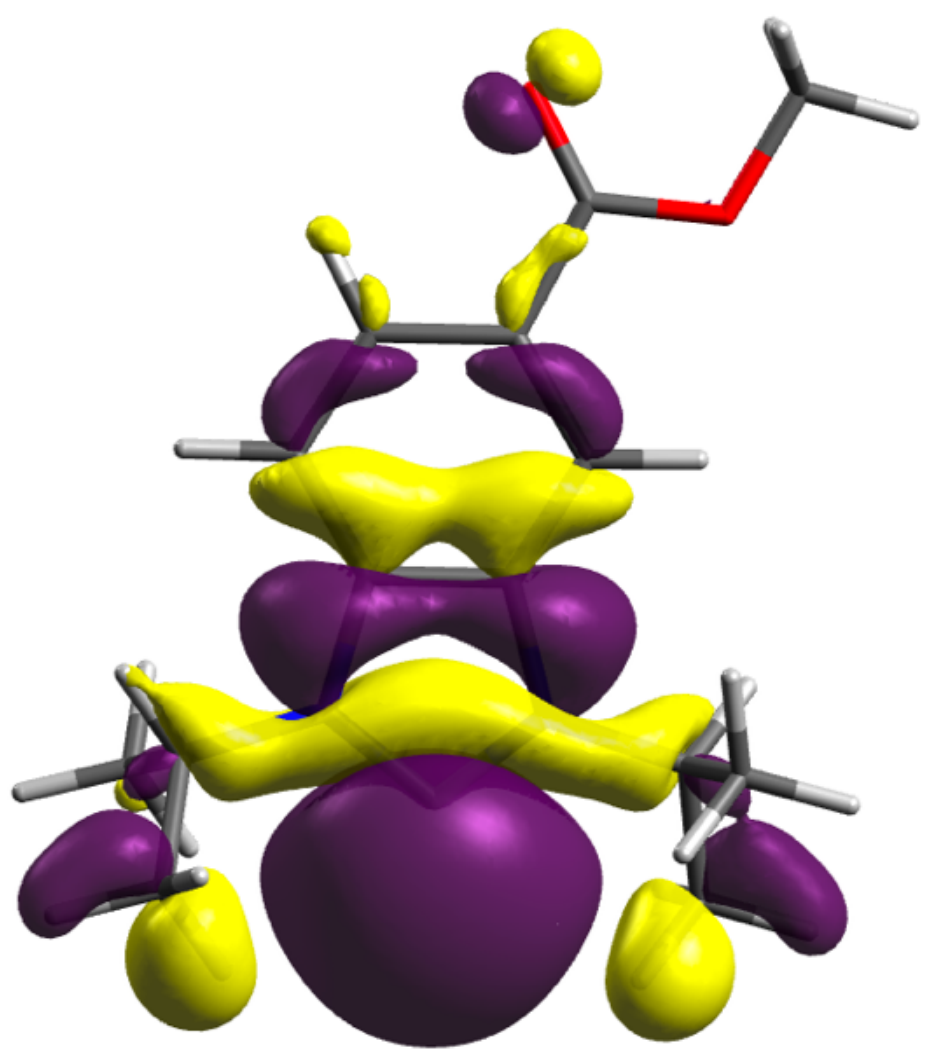

Figure S54 Depiction of the HOMO for R = CO_2Me; Hydrogen atoms are depicted in white, carbon in grey, nitrogen in blue and oxygen in red; isovalue = 0.015 e bohr^{-3}.

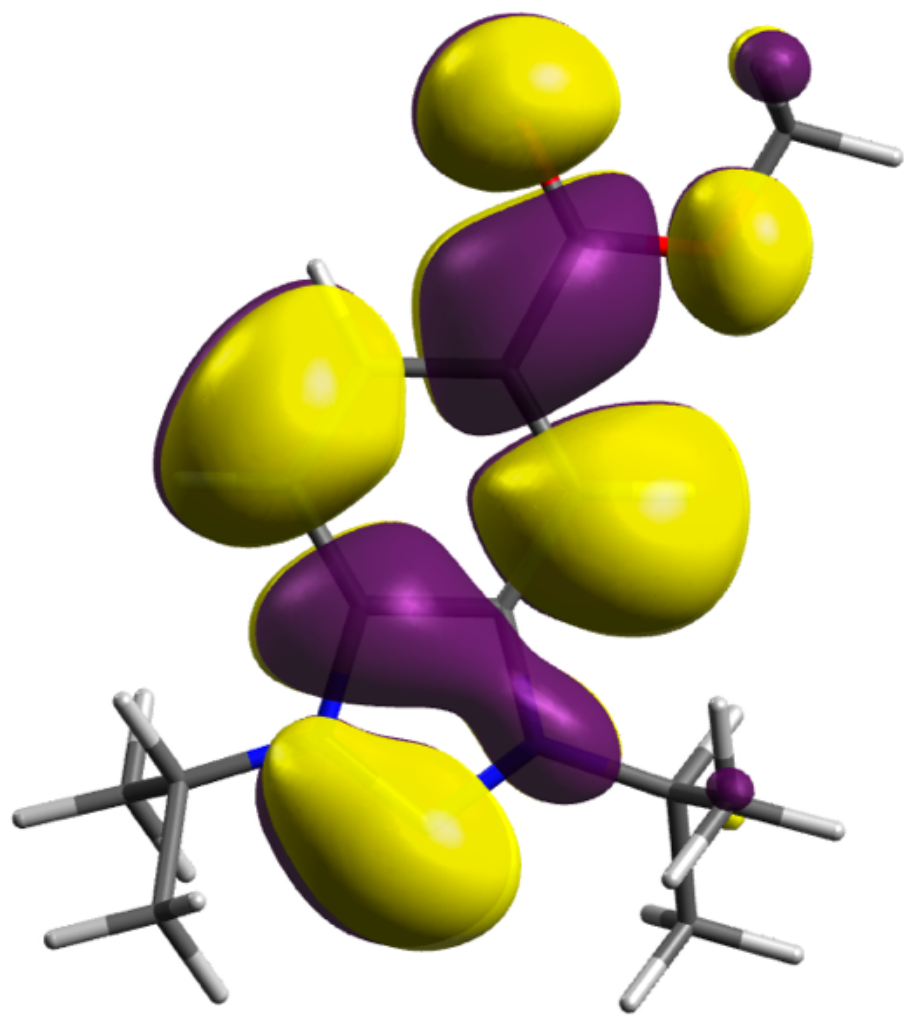

Figure S55 Depiction of the LUMO for R = CO_2Me; Hydrogen atoms are depicted in white, carbon in grey, nitrogen in blue and oxygen in red; isovalue = 0.015 e bohr^{-3}.

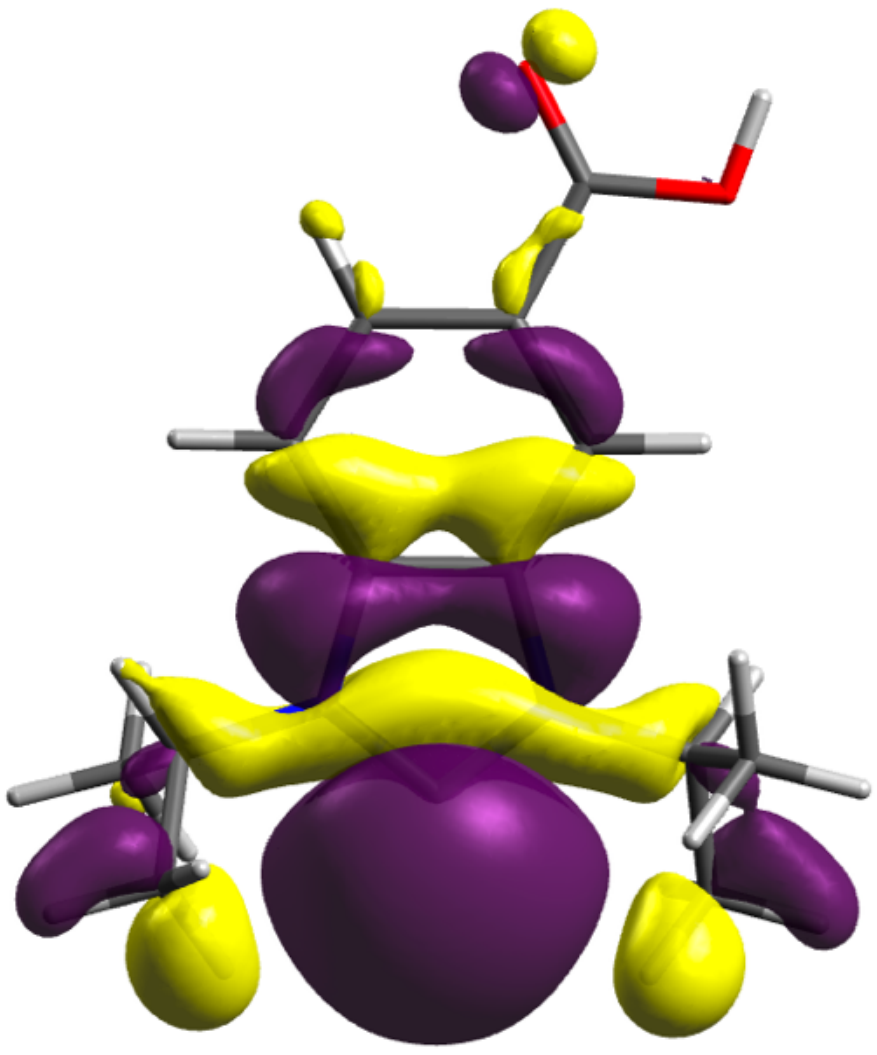

Figure S56 Depiction of the HOMO for R = CO_2H; Hydrogen atoms are depicted in white, carbon in grey, nitrogen in blue and oxygen in red; isovalue = 0.015 e $bohr^{-3}$.

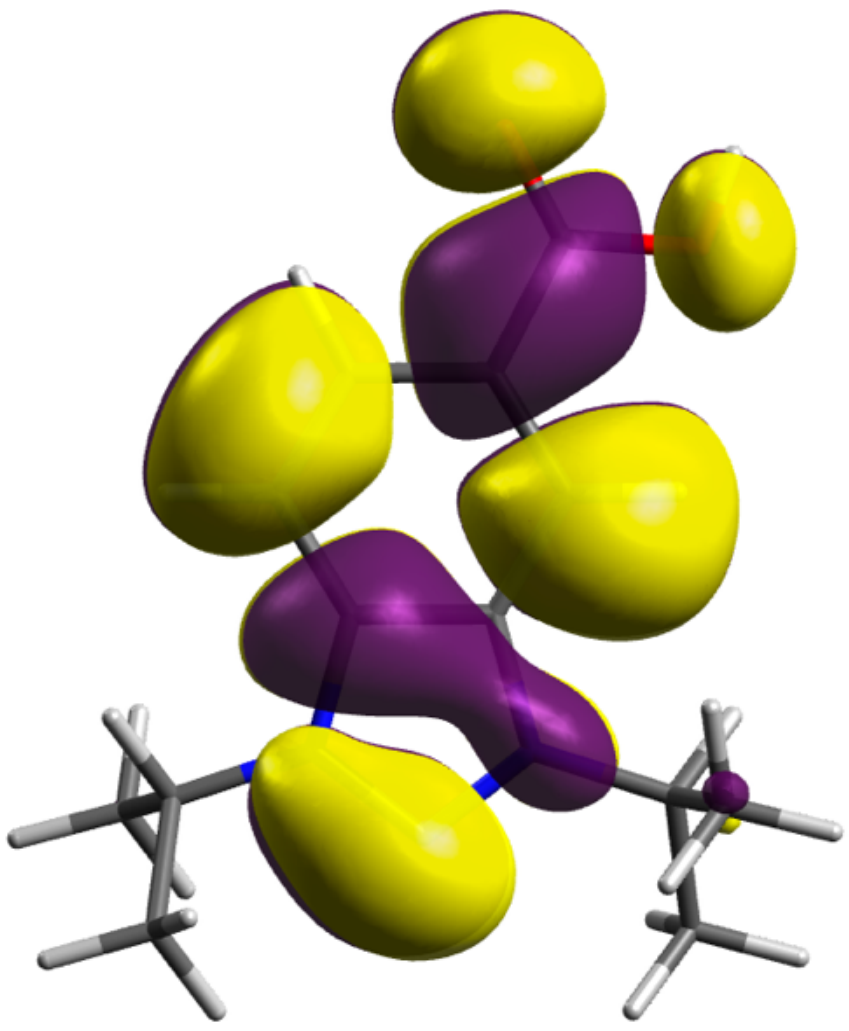

Figure S57 Depiction of the LUMO for R = CO_2H; Hydrogen atoms are depicted in white, carbon in grey, nitrogen in blue and oxygen in red; isovalue = 0.015 e $bohr^{-3}$.

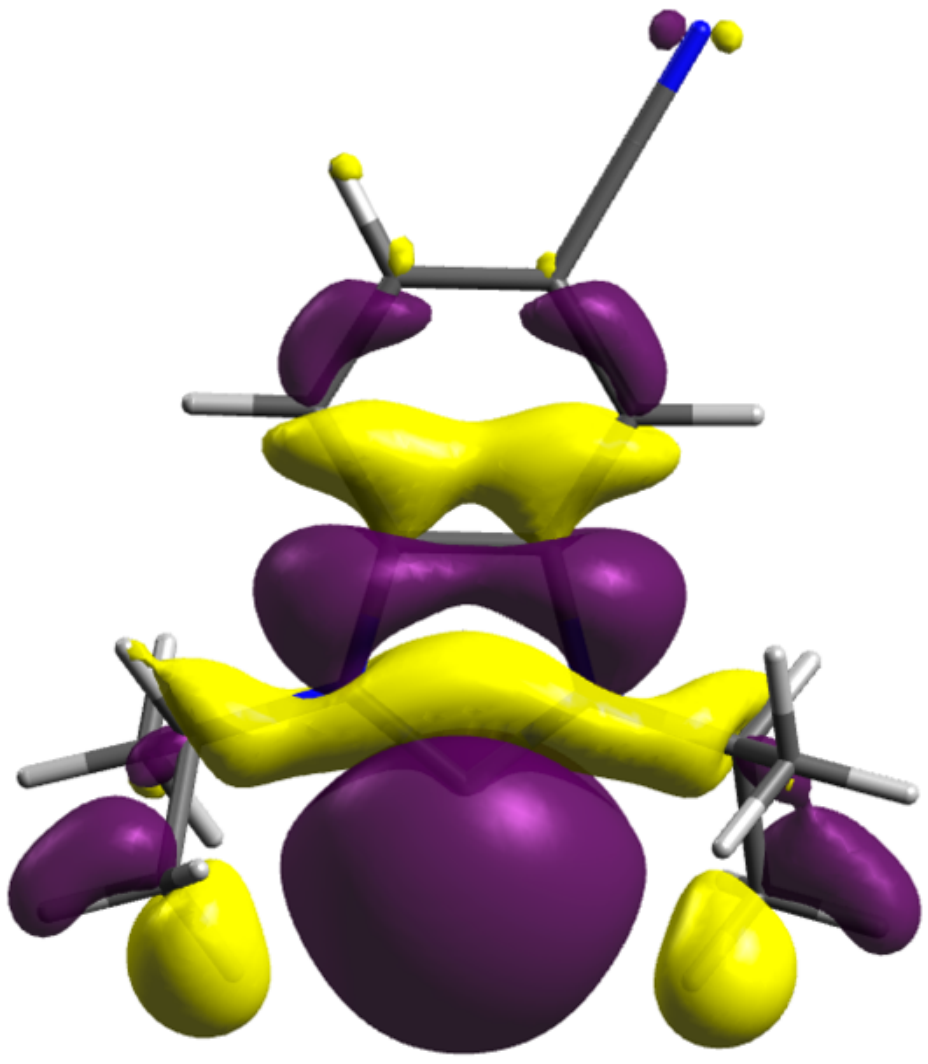

Figure S58 Depiction of the HOMO for R = CO_2H; Hydrogen atoms are depicted in white, carbon in grey and nitrogen in blue; isovalue = 0.015 e bohr^{-3}.

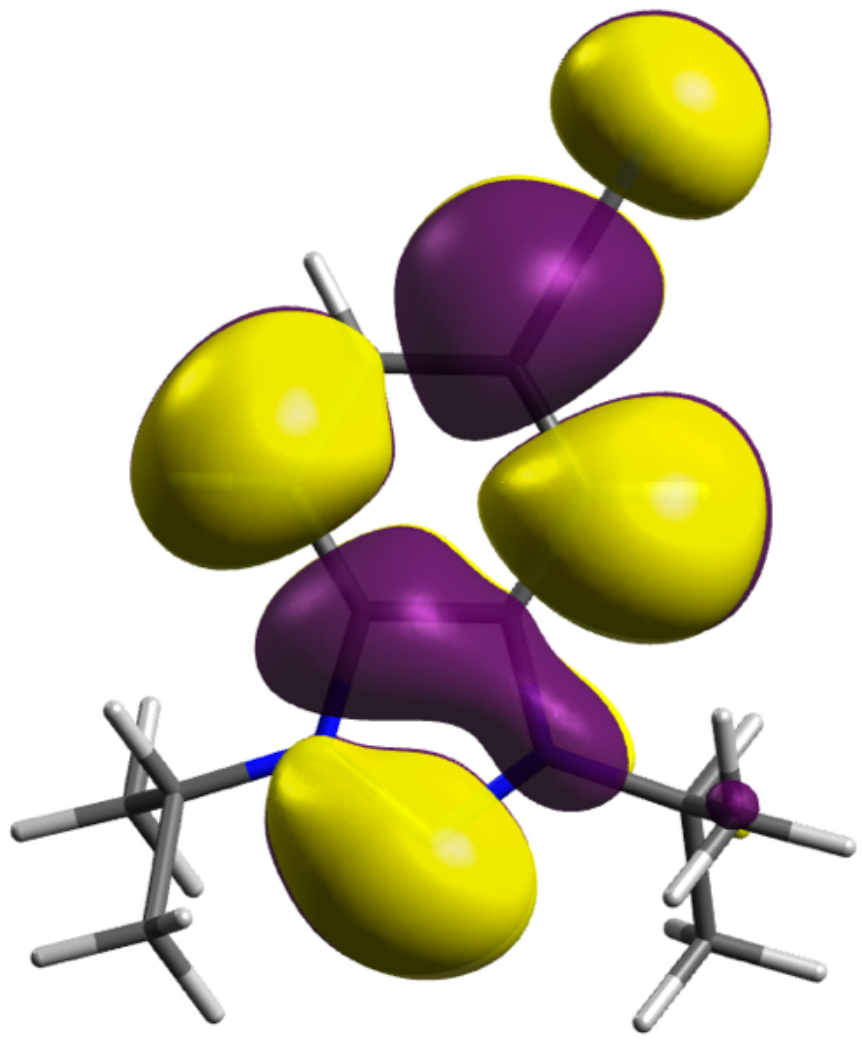

Figure S59 Depiction of the LUMO for R = CN; Hydrogen atoms are depicted in white, carbon in grey and nitrogen in blue; isovalue = 0.015 e bohr^{-3}.

S8 EDA-NOCV Analysis

Tabel S4 Table summarising results from EDA-NOCV: Exchange Repulsion, Dispersion Contribution and Other Orbital Interactions. The values in parenthesis for the later are the % contribution of the the latter with respect to the total orbital energy.

R	σ_p	σ_{p^-}	Exchange Repulsion / kcal mol^{-1}	Dispersion Contribution / kcal mol^{-1}	Other Orbital Interactions / kcal mol^{-1} (%)
NH_2	-0.66	-0.15	269.35	-20.54	-16.39 (18.73)
OMe	-0.27	-0.26	268.4	-20.54	-16.19 (18.58)
H	0.00	0.00	267.83	-20.47	-15.97 (18.40)
F	0.06	-0.03	266.89	-20.47	-15.79 (18.24)
I	0.18	0.27	266.23	-20.53	-15.77 (18.21)
Br	0.23	0.25	266.33	-20.51	-15.77 (18.22)
CHO	0.42	1.03	266.02	-20.48	-15.64 (18.11)
CO_2Me	0.45	0.75	266.66	-20.51	-15.75 (18.19)
CO_2H	0.45	0.77	266.36	-20.49	-15.69 (18.14)
CN	0.66	1.00	265.04	-20.48	-15.51 (18.00)
NO_2	0.81	1.27	264.97	-20.47	-15.43 (17.91)

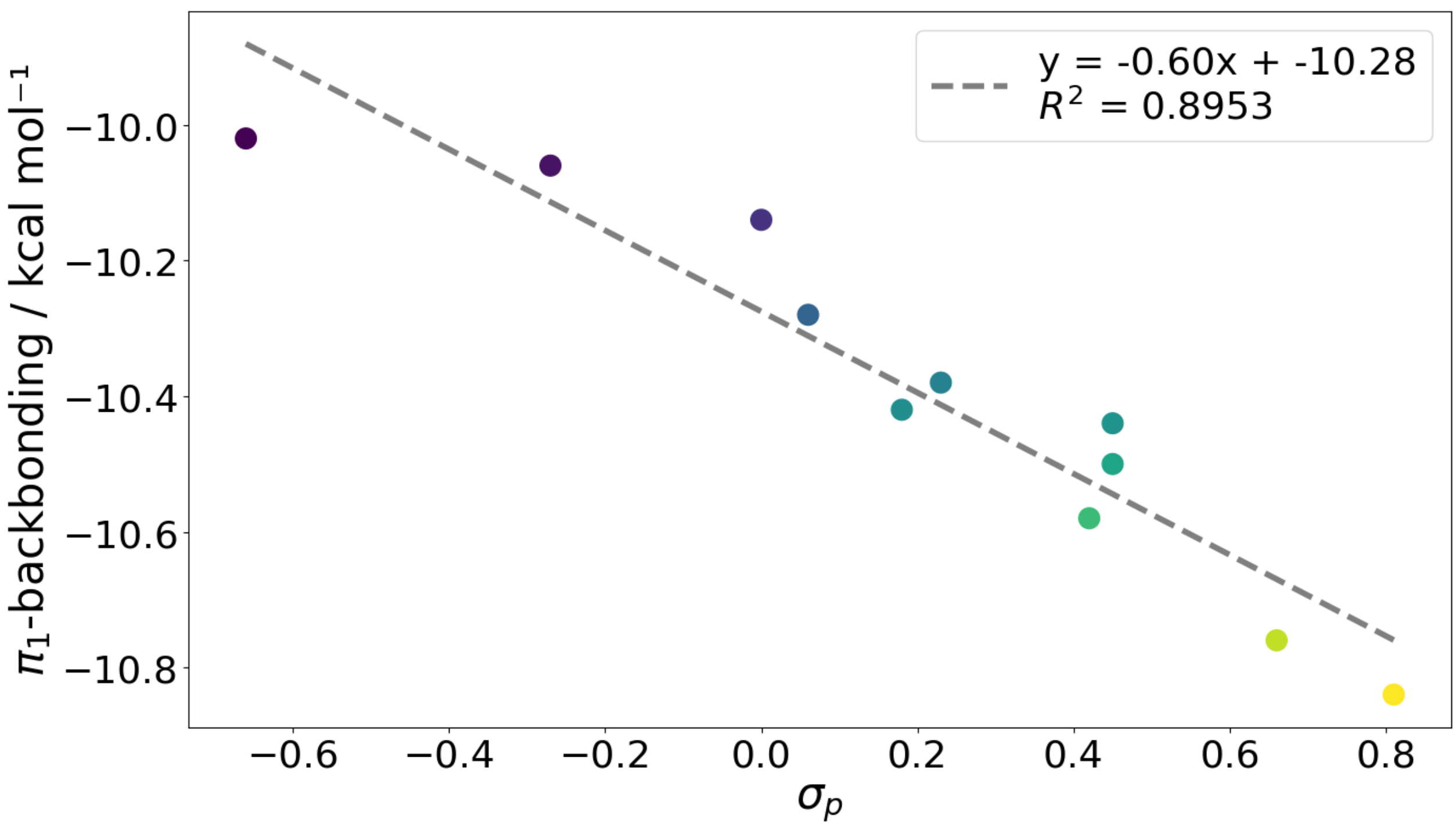

Figure S60 π_1-backbonding plotted as a function of σ_p.

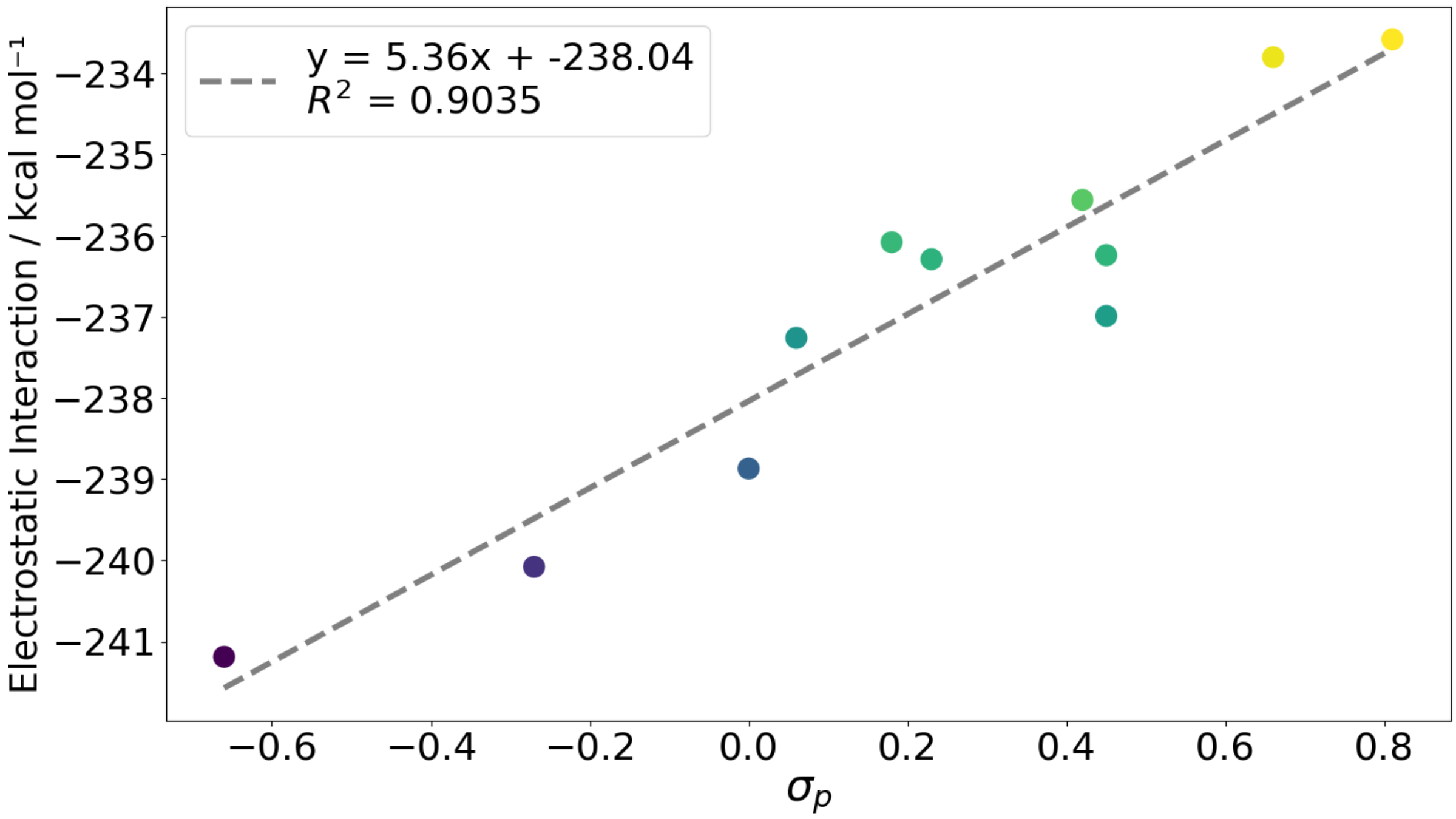

Figure S61 Electrostatic interactions plotted as a function of σ_p.

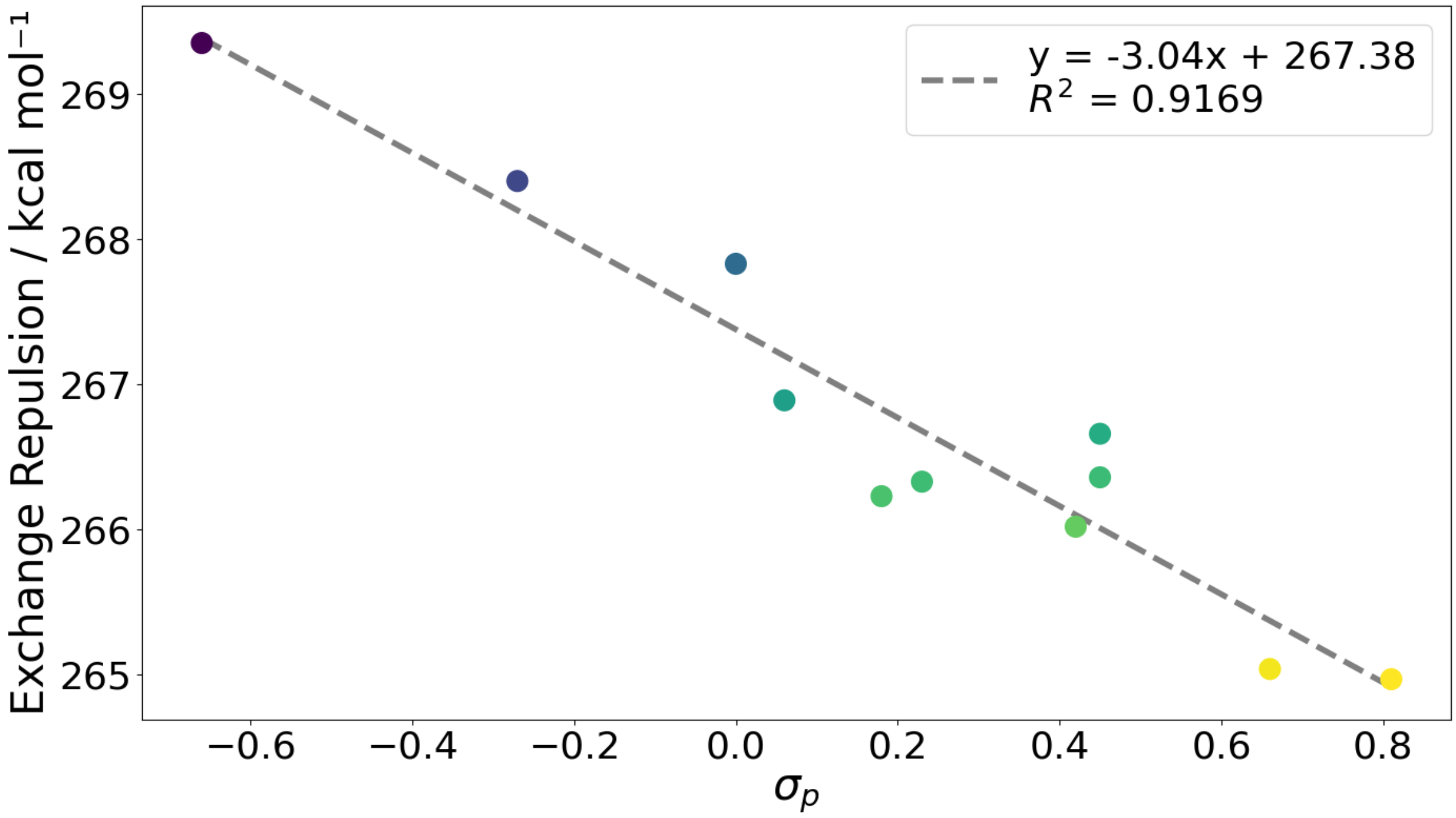

Figure S62 Exchange repulsion plotted as a function of σ_p.

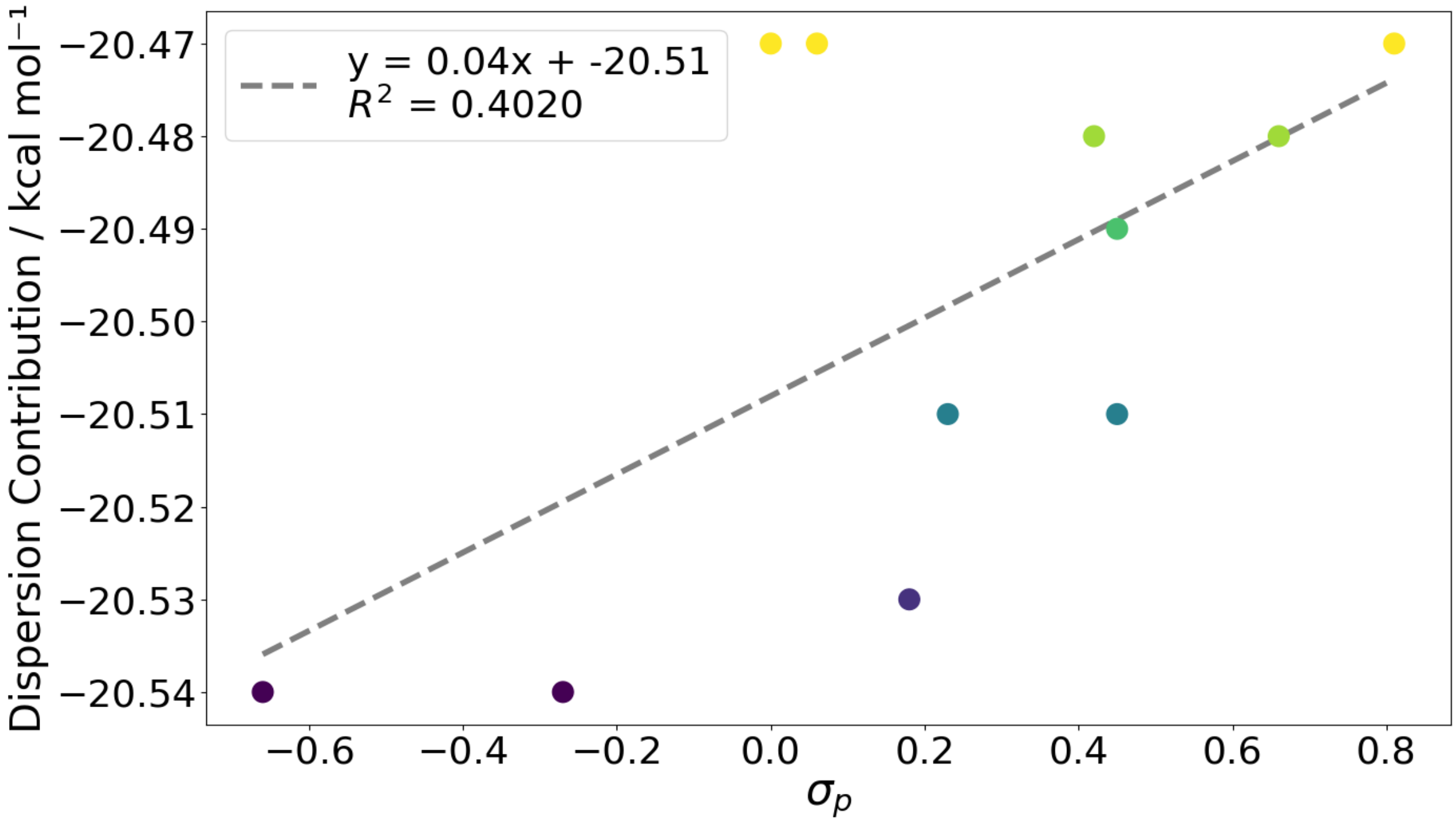

Figure S63 Dispersion contribution plotted as a function of σ_p.

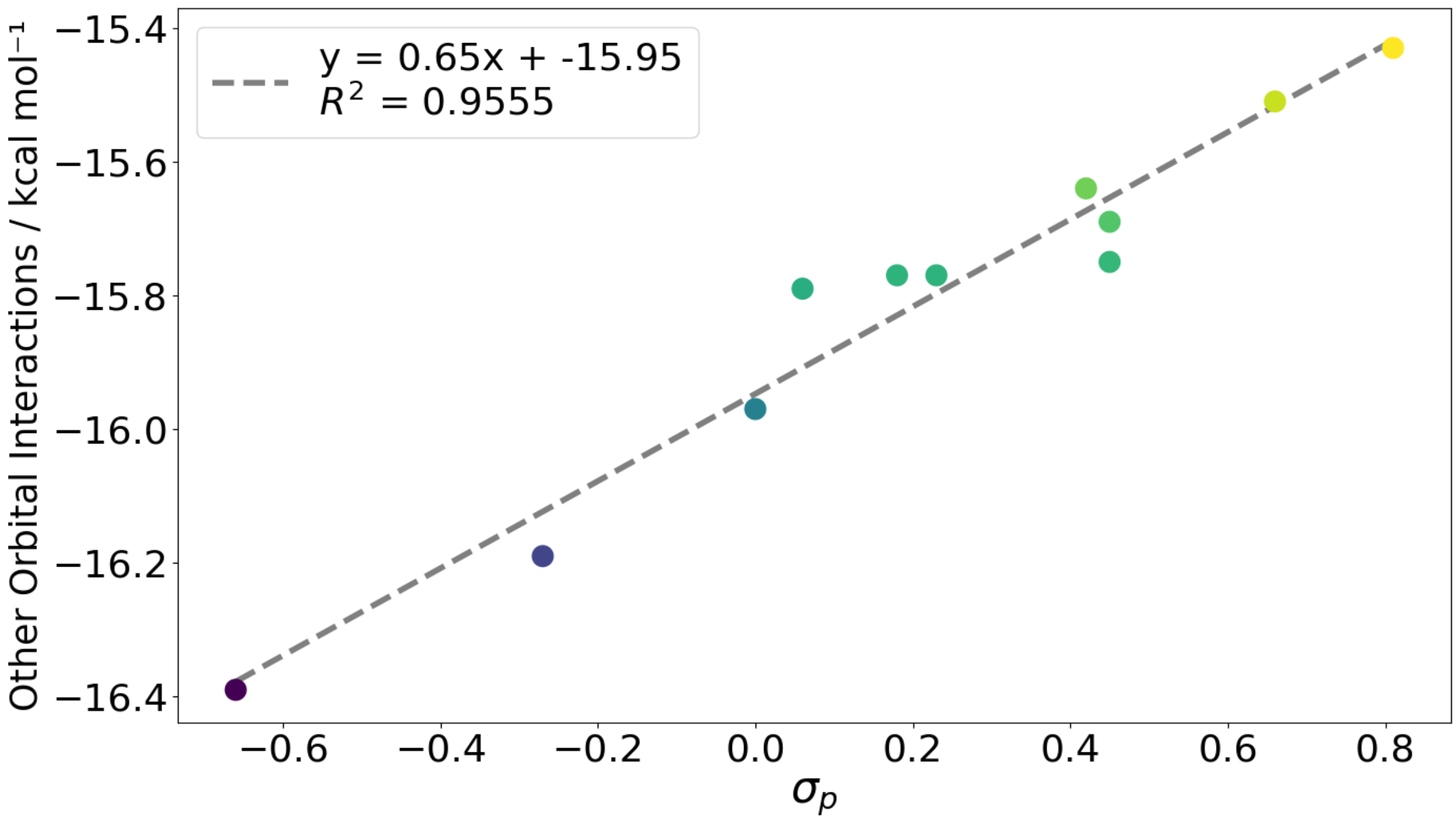

Figure S64 Other orbital interactions plotted as a function of σ_p.

S9 NMR Spectra

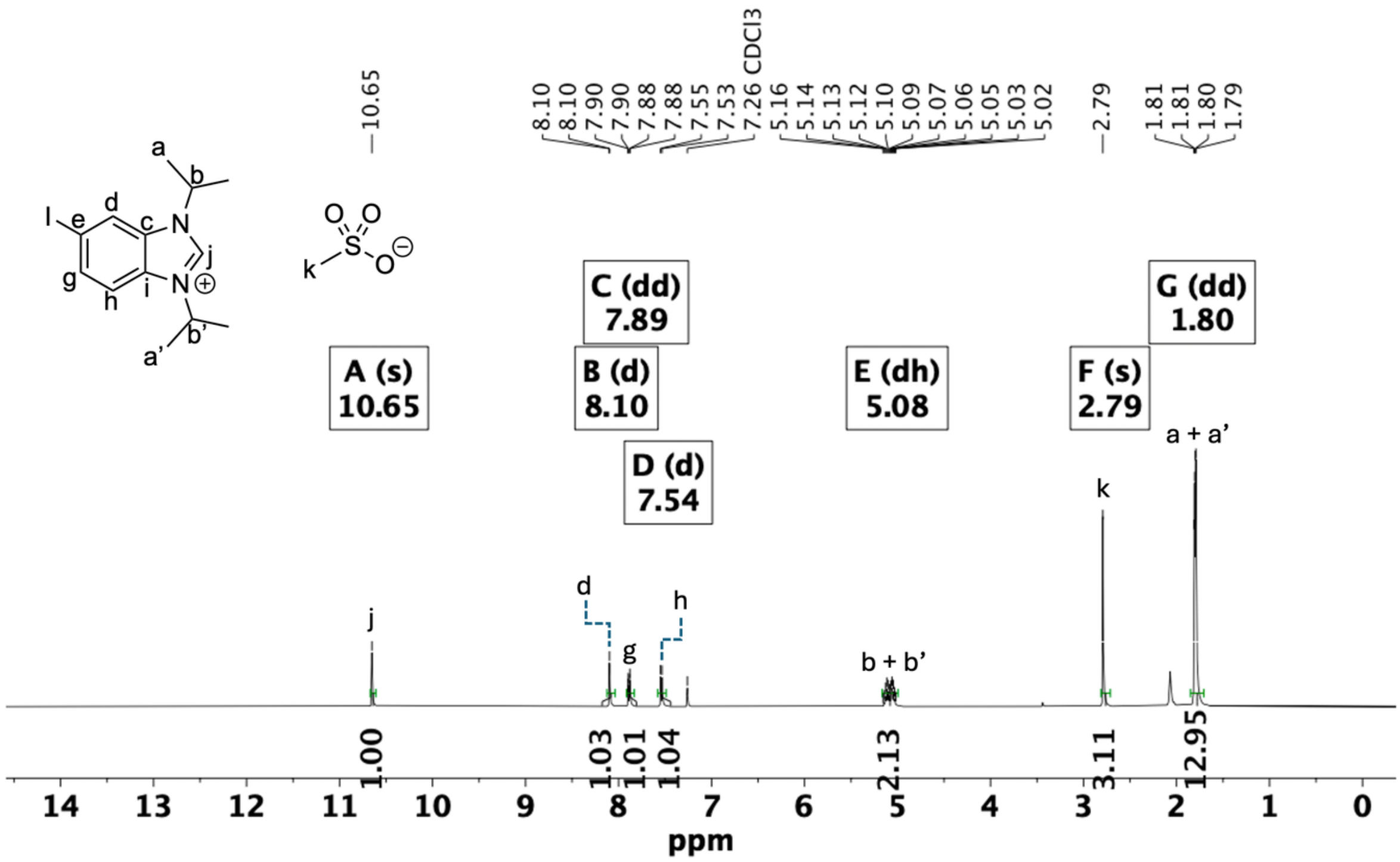

Figure S65 500 MHz ^{1}H NMR spectrum for R = I in $CDCl_3$.

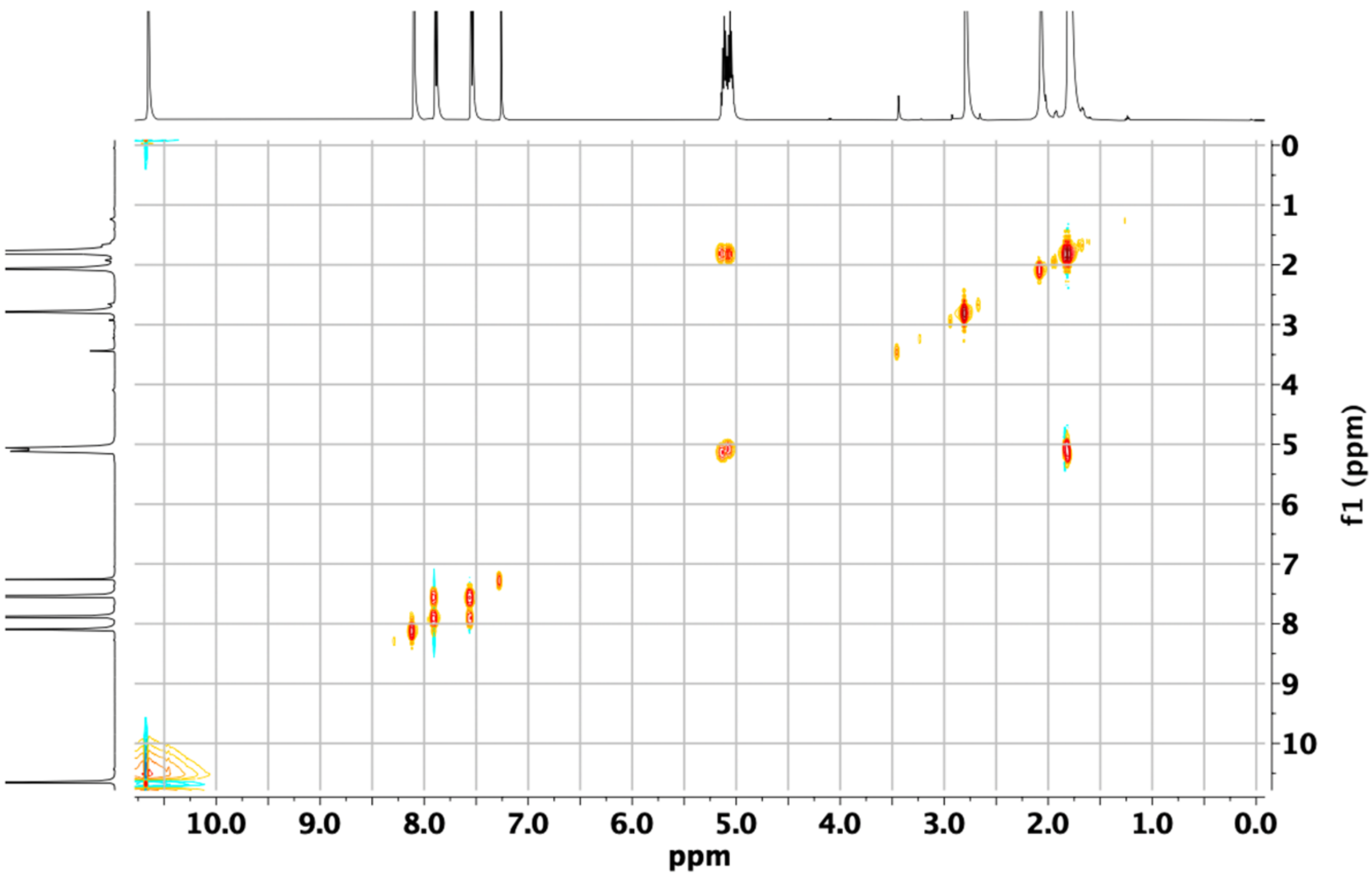

Figure S66 ^{1}H-^{1}H COSY spectrum for R = I in $CDCl_3$.

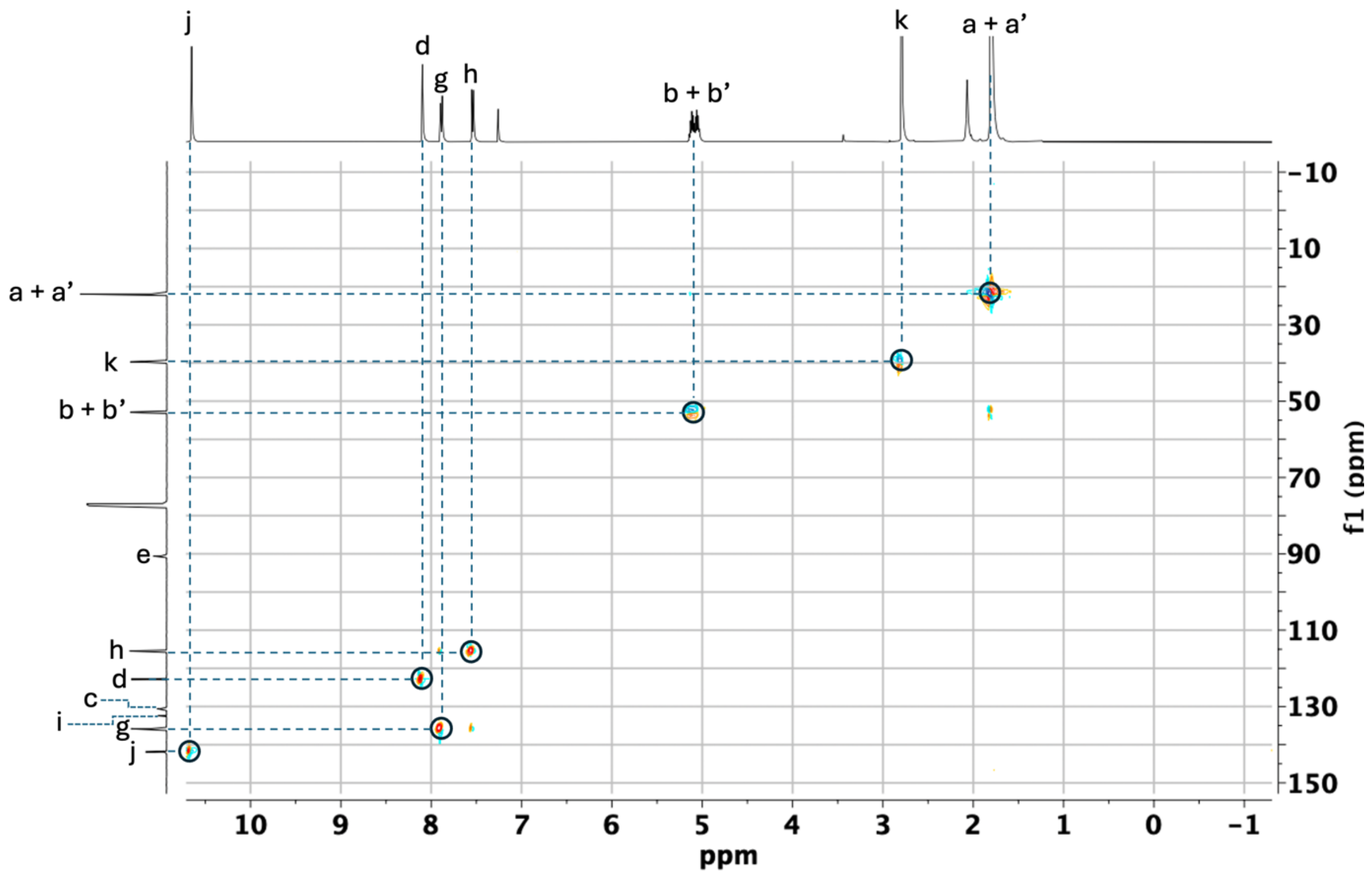

Figure S67 ^1H-^{13}C HSQC spectrum for R = I in $CDCl_3$.

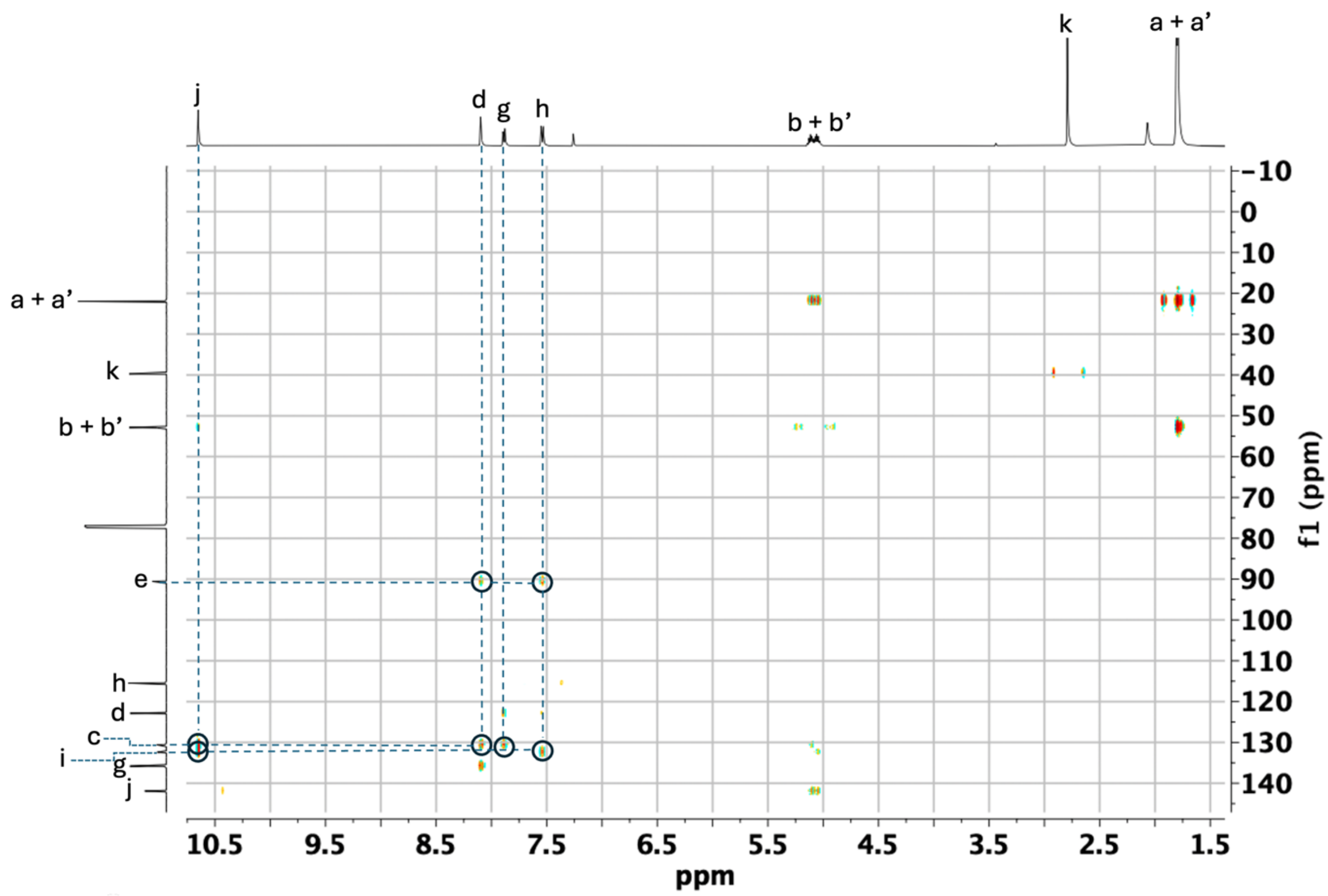

Figure S68 ^1H-^{13}C HMBC spectrum for R = I in $CDCl_3$.

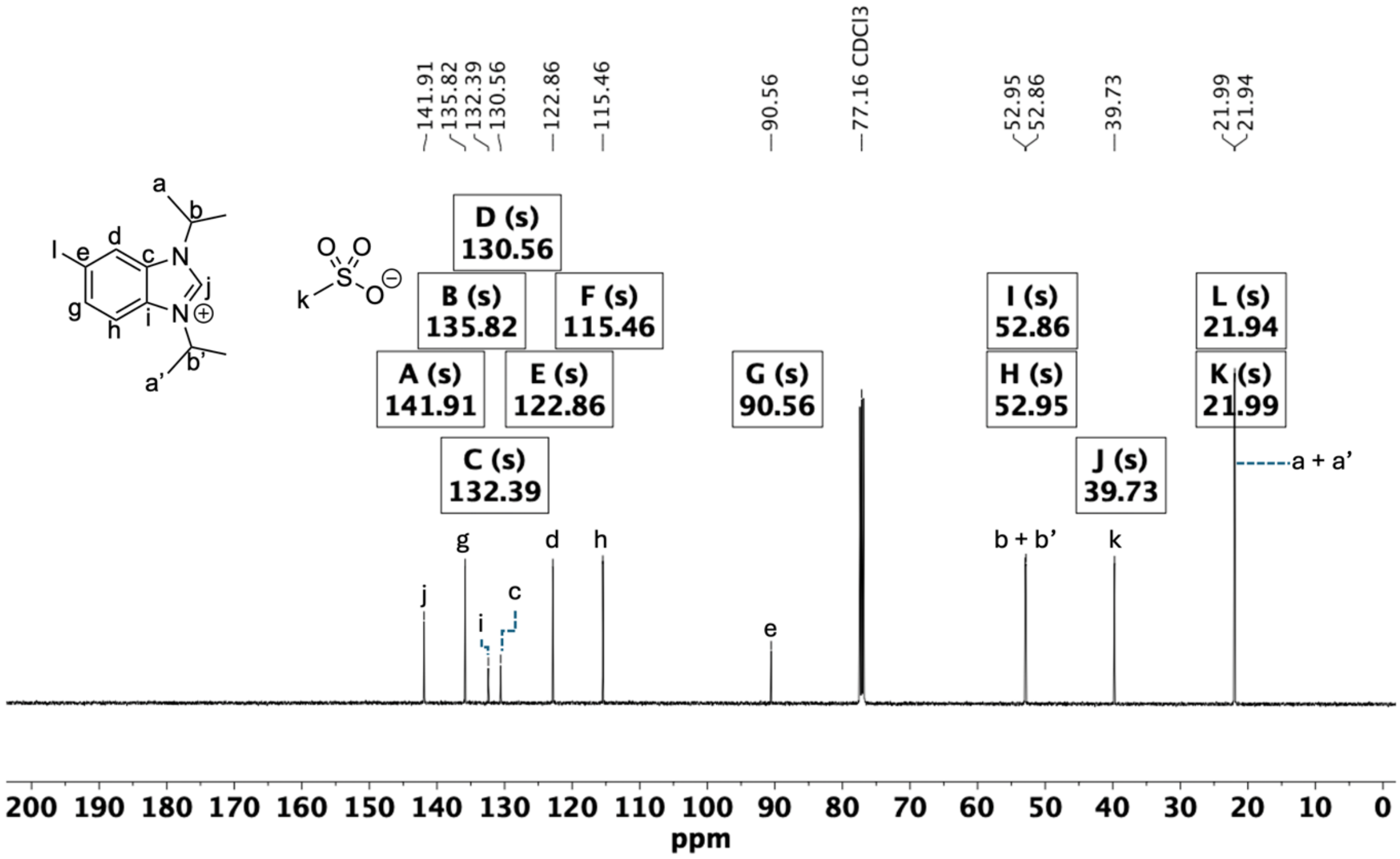

Figure S69 126 MHz ^{13}C NMR spectrum for R = I in $CDCl_3$.

S10 DFT Coordinates

During the optimisation the gold atoms in the two layers were frozen in space, therefore the coordinates will be identical for all derivatives. Hence, the surface here is reported once and then for each derivative the coordinates of the ligand and adatom are reported separately. To obtain the final geometry the coordinates of the surface, and adatom and ligand must be combined. Since the geometries for the EDA—NOCV was extracted from the optimised geometry used to compute the SERS spectra, and only modified by reducing the size of the Au-cluster, the input (and output) geometry can be obtained using the same methodology.

S9.1 DFT Coordinates for the Computed SERS Spectra and NOCV

Surface for computed SERS Spectra:

Au	4.35943	2.78311	-4.35203
Au	-1.34900	3.93492	-1.79444
Au	5.56286	1.46587	-2.22220
Au	0.02166	1.50997	-2.18864
Au	0.00549	-3.34742	-2.64858
Au	5.58672	-3.35504	-2.71102
Au	2.74901	-3.33851	-2.69624
Au	5.73585	0.28669	-4.72013
Au	-1.42770	2.69495	-4.25980
Au	-2.78554	0.22428	-4.50547
Au	-4.06371	3.84161	-1.82188
Au	2.78849	0.20215	-4.52416
Au	-2.72346	1.40909	-1.79270
Au	-5.52501	1.47806	-2.16805
Au	-5.74697	0.31280	-4.67020
Au	-4.35657	2.79795	-4.30892
Au	-5.57377	-3.34248	-2.67369
Au	2.76670	1.40721	-1.82924
Au	1.40239	3.93554	-1.82169
Au	0.00381	0.24368	-4.69691
Au	-1.39587	-2.16206	-4.94936
Au	4.16456	-0.96973	-2.21764
Au	4.23773	-2.23174	-4.90498
Au	1.43907	2.68380	-4.28549
Au	-2.73964	-3.33147	-2.69523
Au	-1.40879	-0.99022	-2.18853
Au	-4.14016	-0.95662	-2.19414
Au	1.42953	-0.99026	-2.18858
Au	6.90514	-0.97558	-2.58980
Au	-6.88447	-0.96281	-2.53361
Au	1.38787	-2.17517	-4.95337
Au	4.11904	3.83670	-1.86628
Au	-4.24911	-2.19965	-4.87629

Surface for EDA–NOCV analysis:

Au	-1.34900	3.93492	-1.79444
Au	0.02156	1.51004	-2.18858
Au	0.00549	-3.34742	-2.64858
Au	2.74901	-3.33851	-2.69624
Au	-1.42769	2.69493	-4.25982
Au	-2.78557	0.22427	-4.50546
Au	2.78850	0.20214	-4.52415
Au	-2.72346	1.40909	-1.79270
Au	2.76670	1.40719	-1.82924
Au	1.40239	3.93554	-1.82170
Au	0.00383	0.24369	-4.69691
Au	-1.39588	-2.16205	-4.94936
Au	4.16456	-0.96973	-2.21764
Au	1.43907	2.68378	-4.28550
Au	-2.73965	-3.33146	-2.69524
Au	-1.40867	-0.99027	-2.18858
Au	-4.14016	-0.95662	-2.19414
Au	1.42952	-0.99027	-2.18858
Au	1.38786	-2.17517	-4.95337

R = NH_2

Au	0.00315	0.00557	0.01372
C	0.01021	-0.71960	1.90041
C	-0.69600	-1.68759	3.82907
C	0.72211	-1.67779	3.82480
C	-2.50079	-0.99644	2.16426
C	2.52055	-0.97598	2.15592
C	-2.87969	0.41404	1.70404
C	2.88877	0.43395	1.68585
C	2.79565	-2.05816	1.10526
C	-2.76906	-2.07449	1.10756

C	1.44145	-2.22906	4.89786
C	-1.43306	-2.24077	4.88545
C	0.71460	-2.77907	5.95737
N	-1.09183	-1.08045	2.63297
N	1.11138	-1.06720	2.62507
H	3.12006	-1.19727	3.06310
H	-3.09965	-1.22669	3.06954
H	-2.65312	1.17140	2.47998
H	2.65521	1.19538	2.45569
H	-2.54704	-3.08876	1.49460
H	2.57636	-3.07141	1.49638
H	-2.34418	0.69922	0.77121
H	2.35231	0.70747	0.75018
H	2.17072	-1.89221	0.20269
H	-2.14190	-1.90256	0.20768
H	-3.96206	0.44884	1.47022
H	3.97128	0.47525	1.45378
H	3.85337	-2.02312	0.77592
H	-3.82568	-2.04186	0.77431
H	-2.53263	-2.24894	4.87840
H	1.25692	-3.21440	6.81119
H	2.54068	-2.23974	4.91398
N	-1.38369	-3.31064	7.07245
C	-0.71593	-2.79556	5.97314
H	-0.86534	-3.94768	7.67805
H	-2.36518	-3.56277	6.95240

R = OMe

Au	0.00312	0.00664	0.01359
C	0.01005	-0.71664	1.90115
C	-0.69282	-1.67856	3.83163
C	0.72333	-1.66561	3.82985
C	-2.50027	-1.00020	2.16384
C	2.52182	-0.96498	2.15974
C	-2.88382	0.40879	1.70339
C	2.88598	0.44608	1.69013
C	2.79837	-2.04636	1.10878
C	-2.76255	-2.07976	1.10741
C	1.44862	-2.20764	4.90713
C	-1.43556	-2.22633	4.89309
C	0.72321	-2.75311	5.96736
N	-1.09051	-1.07824	2.63376
N	1.11212	-1.05938	2.62909
H	3.12181	-1.18539	3.06672
H	-3.09870	-1.23314	3.06883
H	-2.66136	1.16713	2.47958
H	2.65104	1.20673	2.46033
H	-2.53669	-3.09302	1.49484
H	2.58283	-3.06037	1.49997
H	-2.34834	0.69572	0.77116
H	2.34883	0.71841	0.75452
H	2.17238	-1.88212	0.20660
H	-2.13574	-1.90513	0.20786
H	-3.96598	0.43913	1.46818
H	3.96833	0.48995	1.45799
H	3.85564	-2.00796	0.77855
H	-3.81905	-2.05139	0.77360
H	-2.53202	-2.23319	4.87376
H	1.23526	-3.19338	6.83496
H	2.54777	-2.21170	4.92233
H	-3.11740	-3.98940	6.30064
H	-2.93299	-3.88565	8.09549
C	-2.68804	-3.39225	7.13688
O	-1.27144	-3.33462	7.06602
C	-0.70238	-2.76723	5.96894
H	-3.14209	-2.37540	7.12507

R = H

Au	0.00225	0.00702	0.01338
C	0.00790	-0.70216	1.90615
C	-0.69320	-1.62712	3.85267
C	0.72714	-1.61974	3.84956
C	-2.50206	-0.97006	2.17410
C	2.52171	-0.94556	2.16281
C	-2.88275	0.43871	1.71071
C	2.88562	0.46431	1.68923
C	2.79246	-2.02997	1.11360
C	-2.76582	-2.05133	1.11989
C	1.46273	-2.14044	4.92723
C	-1.41855	-2.15523	4.93368
C	0.73495	-2.66587	6.00552
C	-0.68058	-2.67310	6.00870
N	-1.09057	-1.04775	2.64306
N	1.11318	-1.03616	2.63831
H	3.12459	-1.16510	3.06790
H	-3.09856	-1.20154	3.08043
H	-2.65975	1.19825	2.48556
H	2.65572	1.22655	2.45939
H	-2.53936	-3.06402	1.50831
H	2.57905	-3.04289	1.50871
H	-2.34664	0.72255	0.77785
H	2.34478	0.73596	0.75555
H	2.16290	-1.86853	0.21334
H	-2.14254	-1.87807	0.21758
H	-3.96486	0.47022	1.47560
H	3.96694	0.50609	1.45206
H	3.84817	-1.99221	0.77838
H	-3.82354	-2.02343	0.79015

H	-2.51773	-2.16888	4.94085
H	-1.21531	-3.09651	6.87220
H	1.27785	-3.08381	6.86659
H	2.56201	-2.14289	4.92940

R = F

Au	0.00206	0.00790	0.01325
C	0.00977	-0.70300	1.90511
C	-0.69340	-1.63458	3.84736
C	0.72708	-1.62652	3.84558
C	-2.50274	-0.97496	2.17316
C	2.52312	-0.94772	2.16206
C	-2.88377	0.43405	1.71115
C	2.88698	0.46214	1.68887
C	2.79460	-2.03222	1.11326
C	-2.76520	-2.05562	1.11813
C	1.45873	-2.15124	4.92413
C	-1.42712	-2.16395	4.92172
C	0.73703	-2.68132	6.00227
N	-1.09079	-1.05227	2.64223
N	1.11348	-1.03841	2.63584
H	3.12494	-1.16688	3.06808
H	-3.09931	-1.20727	3.07935
H	-2.66127	1.19307	2.48663
H	2.65630	1.22444	2.45872
H	-2.53947	-3.06874	1.50587
H	2.57994	-3.04519	1.50760
H	-2.34813	0.71872	0.77833
H	2.34700	0.73331	0.75462
H	2.16662	-1.86982	0.21211
H	-2.14116	-1.88150	0.21653
H	-3.96593	0.46495	1.47636
H	3.96849	0.50389	1.45270
H	3.85084	-1.99506	0.77978
H	-3.82258	-2.02732	0.78746
H	-2.52428	-2.19113	4.95419
H	1.25077	-3.10848	6.87521
H	2.55774	-2.15554	4.93186
C	-0.67550	-2.67922	5.98464
F	-1.32772	-3.20051	7.04016

R = I

Au	0.00218	0.00869	0.01327
C	0.00765	-0.70511	1.90375
C	-0.69449	-1.64264	3.84324
C	0.72555	-1.63654	3.83991
C	-2.50478	-0.97364	2.17148
C	2.52244	-0.95533	2.16002
C	-2.88268	0.43622	1.70983
C	2.88754	0.45496	1.68928
C	2.79174	-2.03861	1.10952
C	-2.77007	-2.05435	1.11733
C	1.45516	-2.16751	4.91611
C	-1.42750	-2.17655	4.91555
C	0.73288	-2.70194	5.99271
N	-1.09221	-1.05449	2.63928
N	1.11257	-1.04548	2.63419
H	3.12396	-1.17681	3.06568
H	-3.10127	-1.20398	3.07814
H	-2.65703	1.19475	2.48486
H	2.65747	1.21624	2.46032
H	-2.54698	-3.06774	1.50588
H	2.57749	-3.05205	1.50286
H	-2.34833	0.71950	0.77586
H	2.34851	0.72819	0.75510
H	2.16257	-1.87486	0.20943
H	-2.14595	-1.88236	0.21538
H	-3.96523	0.46958	1.47728
H	3.96922	0.49583	1.45381
H	3.84746	-2.00124	0.77450
H	-3.82752	-2.02357	0.78719
H	-2.52562	-2.18928	4.92119
H	1.27272	-3.12720	6.85049
H	2.55436	-2.17463	4.92534
I	-1.70908	-3.52607	7.63065
C	-0.68471	-2.70302	5.98539

R = Br

Au	0.00212	0.00846	0.01322
C	0.00829	-0.70366	1.90434
C	-0.69361	-1.63762	3.84554
C	0.72643	-1.63068	3.84256
C	-2.50407	-0.97351	2.17243
C	2.52288	-0.95129	2.16106
C	-2.88345	0.43587	1.71044
C	2.88725	0.45888	1.68936
C	2.79289	-2.03505	1.11121
C	-2.76806	-2.05450	1.11822
C	1.45641	-2.15860	4.92010
C	-1.42672	-2.16895	4.91874
C	0.73474	-2.69064	5.99775
N	-1.09157	-1.05255	2.64057
N	1.11310	-1.04176	2.63528
H	3.12461	-1.17186	3.06680
H	-3.10032	-1.20462	3.07905
H	-2.65904	1.19477	2.48547

H	2.65694	1.22053	2.45997
H	-2.54359	-3.06763	1.50666
H	2.57881	-3.04834	1.50505
H	-2.34883	0.71957	0.77675
H	2.34780	0.73124	0.75517
H	2.16397	-1.87201	0.21082
H	-2.14425	-1.88167	0.21621
H	-3.96590	0.46802	1.47723
H	3.96885	0.50020	1.45358
H	3.84873	-1.99754	0.77654
H	-3.82557	-2.02506	0.78815
H	-2.52458	-2.18583	4.93194
H	1.26729	-3.11528	6.86025
H	2.55552	-2.16495	4.92941
C	-0.68160	-2.69105	5.98758
Br	-1.60818	-3.43172	7.47861

R = CHO

Au	0.00224	0.00912	0.01293
C	0.00523	-0.69599	1.90584
C	-0.69448	-1.61660	3.85341
C	0.72801	-1.60931	3.85017
C	-2.50549	-0.96259	2.17513
C	2.52250	-0.93810	2.16504
C	-2.88527	0.44614	1.71166
C	2.88315	0.47306	1.69349
C	2.79153	-2.02142	1.11461
C	-2.76898	-2.04499	1.12233
C	1.46872	-2.12824	4.93064
C	-1.41436	-2.14166	4.93387
C	0.74884	-2.64958	6.00745
N	-1.09211	-1.04012	2.64266
N	1.11276	-1.03085	2.64189
H	3.12568	-1.15841	3.06959
H	-3.10034	-1.19338	3.08286
H	-2.66090	1.20614	2.48563
H	2.65239	1.23403	2.46464
H	-2.54258	-3.05756	1.51115
H	2.58221	-3.03527	1.50953
H	-2.35122	0.72854	0.77732
H	2.34313	0.74474	0.75948
H	2.16013	-1.86073	0.21552
H	-2.14709	-1.87200	0.21906
H	-3.96780	0.47756	1.47888
H	3.96461	0.51613	1.45750
H	3.84632	-1.98048	0.77714
H	-3.82700	-2.01706	0.79390
H	-2.51513	-2.16161	4.95357
H	1.26147	-3.07255	6.88477
H	2.56801	-2.12840	4.92840
O	-0.84744	-3.70040	8.16962
C	-1.39311	-3.22939	7.18564
C	-0.67345	-2.65922	6.01478
H	-2.52214	-3.19484	7.09780

R = CO_2Me

Au	0.00257	0.00848	0.01318
C	0.00564	-0.70216	1.90444
C	-0.69552	-1.63010	3.84835
C	0.72594	-1.62372	3.84562
C	-2.50521	-0.97000	2.17276
C	2.52174	-0.94844	2.16333
C	-2.88575	0.43888	1.71031
C	2.88534	0.46225	1.69236
C	2.79104	-2.03140	1.11244
C	-2.76954	-2.05155	1.11918
C	1.45972	-2.14865	4.92465
C	-1.42394	-2.15771	4.92143
C	0.73272	-2.67436	5.99731
N	-1.09231	-1.04801	2.63932
N	1.11229	-1.03961	2.63863
H	3.12397	-1.16991	3.06832
H	-3.09904	-1.20213	3.08063
H	-2.66120	1.19827	2.48484
H	2.65512	1.22329	2.46360
H	-2.54318	-3.06419	1.50778
H	2.57899	-3.04507	1.50638
H	-2.35185	0.72248	0.77615
H	2.34579	0.73520	0.75838
H	2.16075	-1.86896	0.21287
H	-2.14749	-1.87842	0.21601
H	-3.96837	0.47049	1.47778
H	3.96693	0.50404	1.45661
H	3.84636	-1.99244	0.77629
H	-3.82766	-2.02352	0.79092
H	-2.52133	-2.17740	4.94156
H	1.24548	-3.10160	6.87149
H	2.55882	-2.15307	4.92988
H	-3.20380	-3.23375	9.13246
O	-0.78599	-3.74046	8.16257
C	-3.44631	-3.77795	8.19715
C	-1.36697	-3.27205	7.19743
H	-4.51659	-3.66372	7.94863
O	-2.72073	-3.22725	7.08613
C	-0.68768	-2.68217	6.00198
H	-3.19221	-4.84791	8.33986

R = COOH

Au	0.00233	0.00867	0.01307
C	0.00554	-0.69951	1.90507
C	-0.69500	-1.62289	3.85104
C	0.72668	-1.61684	3.84785
C	-2.50546	-0.96572	2.17450
C	2.52208	-0.94500	2.16379
C	-2.88542	0.44317	1.71165
C	2.88531	0.46558	1.69229
C	2.79065	-2.02841	1.11324
C	-2.76991	-2.04756	1.12130
C	1.46152	-2.13911	4.92770
C	-1.42254	-2.14758	4.92612
C	0.73580	-2.66190	6.00228
N	-1.09219	-1.04376	2.64102
N	1.11259	-1.03583	2.63982
H	3.12464	-1.16620	3.06862
H	-3.09937	-1.19734	3.08245
H	-2.66059	1.20277	2.48590
H	2.65557	1.22684	2.46345
H	-2.54374	-3.06018	1.51007
H	2.57899	-3.04196	1.50768
H	-2.35165	0.72615	0.77725
H	2.34537	0.73821	0.75847
H	2.16000	-1.86637	0.21385
H	-2.14823	-1.87470	0.21783
H	-3.96808	0.47497	1.47943
H	3.96679	0.50722	1.45602
H	3.84578	-1.98940	0.77653
H	-3.82815	-2.01941	0.79350
H	-2.52008	-2.16591	4.94492
H	1.24949	-3.08674	6.87708
H	2.56058	-2.14344	4.93184
C	-0.68463	-2.66908	6.00734
C	-1.36571	-3.25366	7.20190
O	-0.78966	-3.72097	8.17007
O	-2.72311	-3.21270	7.10148
H	-3.06072	-3.61841	7.92992

R = CN

Au	0.00173	0.00994	0.01282
C	0.00633	-0.69460	1.90567
C	-0.69359	-1.61591	3.85227
C	0.72855	-1.61041	3.84785
C	-2.50620	-0.95823	2.17667
C	2.52385	-0.94023	2.16262
C	-2.88290	0.45139	1.71372
C	2.88601	0.47034	1.69077
C	2.79106	-2.02445	1.11279
C	-2.77131	-2.04035	1.12413
C	1.46429	-2.13113	4.92633
C	-1.42026	-2.13845	4.92816
C	0.74655	-2.65397	6.00663
N	-1.09194	-1.03837	2.64384
N	1.11365	-1.03093	2.63965
H	3.12683	-1.16066	3.06746
H	-3.10165	-1.18752	3.08427
H	-2.65653	1.21083	2.48764
H	2.65647	1.23184	2.46176
H	-2.54802	-3.05345	1.51330
H	2.58026	-3.03797	1.50784
H	-2.34960	0.73271	0.77865
H	2.34618	0.74225	0.75679
H	2.15989	-1.86291	0.21371
H	-2.14901	-1.86892	0.22084
H	-3.96567	0.48474	1.48251
H	3.96742	0.51183	1.45440
H	3.84580	-1.98511	0.77510
H	-3.82925	-2.00987	0.79579
H	-2.51828	-2.15488	4.94474
H	1.28222	-3.07237	6.87046
H	2.56317	-2.13580	4.92887
C	-0.67950	-2.66061	6.01395
C	-1.37637	-3.20924	7.14304
N	-1.94522	-3.65791	8.06758

R = NO_2

Au	0.00156	0.01023	0.01257
C	0.00543	-0.69173	1.90574
C	-0.69516	-1.60586	3.85404
C	0.72871	-1.60142	3.85120
C	-2.50807	-0.95462	2.17871
C	2.52383	-0.93649	2.16433
C	-2.88625	0.45455	1.71601
C	2.88580	0.47401	1.69219
C	2.79006	-2.02089	1.11445
C	-2.77287	-2.03696	1.12641
C	1.46513	-2.11888	4.93275
C	-1.42339	-2.12356	4.93099
C	0.74586	-2.63775	6.01536
N	-1.09267	-1.03298	2.64383
N	1.11351	-1.02670	2.64201
H	3.12680	-1.15725	3.06904
H	-3.10041	-1.18571	3.08783
H	-2.65992	1.21432	2.48962
H	2.65690	1.23563	2.46324
H	-2.54770	-3.04978	1.51520

H	2.57963	-3.03436	1.50979
H	-2.35426	0.73606	0.78022
H	2.34563	0.74583	0.75839
H	2.15858	-1.85946	0.21557
H	-2.15275	-1.86448	0.22180
H	-3.96934	0.48687	1.48628
H	3.96711	0.51521	1.45535
H	3.84462	-1.98148	0.77627
H	-3.83157	-2.00806	0.80053
H	-2.51940	-2.15536	4.98171
H	1.25062	-3.05971	6.89456
H	2.56395	-2.12388	4.93568
O	-2.62899	-3.17510	7.11727
N	-1.39642	-3.19218	7.16173
O	-0.72142	-3.63558	8.09250
C	-0.66572	-2.63047	5.99490

H	2.57963	-3.03436	1.50979
H	-2.35426	0.73606	0.78022
H	2.34563	0.74583	0.75839
H	2.15858	-1.85946	0.21557
H	-2.15275	-1.86448	0.22180
H	-3.96934	0.48687	1.48628
H	3.96711	0.51521	1.45535
H	3.84462	-1.98148	0.77627
H	-3.83157	-2.00806	0.80053
H	-2.51940	-2.15536	4.98171
H	1.25062	-3.05971	6.89456
H	2.56395	-2.12388	4.93568
O	-2.62899	-3.17510	7.11727
N	-1.39642	-3.19218	7.16173
O	-0.72142	-3.63558	8.09250
C	-0.66572	-2.63047	5.99490

S9.1 DFT Coordinates for Free Ligands Utilised for FMOs analysis

R = NH$_2$

C	-3.55657	-1.00882	-0.93140
C	-2.33835	-1.59289	-0.24654
C	-2.66757	-2.14398	1.13255
N	-1.26628	-0.61082	-0.15286
C	-1.46143	0.71641	0.00747
N	-0.20424	1.21815	0.14590
C	0.09762	2.63975	0.24960
C	-0.98973	3.37524	1.00581
C	0.33145	3.22871	-1.13311
C	0.76925	0.23944	0.06585
C	0.07801	-0.95863	-0.11229
C	0.76296	-2.15755	-0.21170
C	2.14562	-2.12386	-0.14021
C	2.84862	-0.92075	0.02952
N	4.24137	-0.92906	0.03073
C	2.15294	0.28259	0.13289
H	-4.32280	-1.78076	-1.03131
H	-3.30385	-0.63218	-1.92369
H	-3.95719	-0.17709	-0.35228
H	-1.94695	-2.40590	-0.86814
H	-3.06936	-1.34630	1.76151
H	-1.77739	-2.54864	1.61883
H	-3.41257	-2.93954	1.06095
H	1.02699	2.70816	0.82564
H	-1.93798	3.30443	0.47347
H	-0.71293	4.42656	1.11122
H	-1.13260	2.94729	1.99933
H	-0.58984	3.17212	-1.71733
H	1.11087	2.68183	-1.66803
H	0.63334	4.27589	-1.05965
H	0.24623	-3.10120	-0.33903
H	2.70566	-3.04907	-0.22297
H	2.68869	1.21750	0.25171
H	4.65315	-1.80378	0.31309
H	4.66559	-0.15112	0.50971

R = OMe

C	-3.48359	-1.88433	-0.94499
C	-2.15908	-2.11733	-0.24812
C	-2.33918	-2.72985	1.13244
N	-1.39042	-0.88286	-0.15400
C	-1.93659	0.34409	0.00335
N	-0.86389	1.16718	0.14398
C	-0.95733	2.61811	0.24882
C	-2.19881	3.03056	1.01274
C	-0.89970	3.24828	-1.13408
C	0.33824	0.48787	0.06912
C	-0.00326	-0.85587	-0.10850
C	0.97572	-1.82529	-0.20362
C	2.30552	-1.42585	-0.13036
C	2.64109	-0.07828	0.03652
O	3.91852	0.37432	0.11131
C	1.65553	0.90028	0.13749
H	-4.01369	-2.83394	-1.04537
H	-3.33312	-1.45801	-1.93798
H	-4.09766	-1.18843	-0.37379
H	-1.55882	-2.79804	-0.86198
H	-2.94483	-2.06614	1.75368
H	-1.37699	-2.87911	1.62695
H	-2.84331	-3.69615	1.06139
H	-0.07750	2.93426	0.81939
H	-3.09580	2.70649	0.48544
H	-2.21635	4.11735	1.11931
H	-2.21409	2.57947	2.00618
H	-1.77397	2.94404	-1.71408
H	-0.00323	2.93470	-1.67322
H	-0.89298	4.33794	-1.05990
H	0.73094	-2.87287	-0.33020
H	3.08054	-2.17568	-0.20451
H	1.94595	1.93551	0.26171
C	4.95777	-0.56484	0.01057
H	5.88587	-0.00117	0.09064
H	4.93757	-1.08786	-0.95212
H	4.91545	-1.30251	0.81977

R = H

C	3.11886	-1.76052	-0.87130
C	2.68613	-0.49230	-0.16486
C	3.22891	-0.41342	1.25366
N	1.23337	-0.36709	-0.15383
C	0.38092	-1.41691	-0.06915
N	-0.84815	-0.84252	-0.00668
C	-2.05411	-1.65497	0.07980
C	-2.93840	-1.47601	-1.14352
C	-2.79442	-1.42033	1.38650
C	0.57574	0.84939	-0.12672
C	-0.78674	0.54285	-0.03908
C	-1.73799	1.55403	-0.00432
C	-1.28827	2.86475	-0.05258

C	0.07146	3.16502	-0.13409
C	1.02542	2.16021	-0.17167
H	4.20960	-1.80125	-0.90835
H	2.73067	-1.79238	-1.89048
H	2.74410	-2.63791	-0.34469
H	3.05712	0.36621	-0.73492
H	4.32094	-0.42850	1.24792
H	2.87221	-1.26815	1.83282
H	2.90004	0.50066	1.75259
H	-1.66470	-2.67478	0.08007
H	-3.77767	-2.17313	-1.09966
H	-2.37300	-1.67500	-2.05549
H	-3.34679	-0.46583	-1.21006
H	-3.63081	-2.11742	1.46883
H	-3.19623	-0.40765	1.45451
H	-2.12862	-1.57978	2.23637
H	-2.79650	1.34200	0.05986
H	-2.01002	3.67241	-0.02484
H	0.38715	4.20094	-0.16680
H	2.08092	2.39615	-0.23016

R = F

C	-3.52948	-0.99043	-0.95664
C	-2.32838	-1.57767	-0.24489
C	-2.68197	-2.10227	1.13805
N	-1.24637	-0.60406	-0.15111
C	-1.43374	0.72693	0.00336
N	-0.17578	1.22233	0.14218
C	0.13313	2.64450	0.24345
C	-0.93642	3.37857	1.02580
C	0.33769	3.23534	-1.14260
C	0.78971	0.23808	0.06897
C	0.09249	-0.96026	-0.10597
C	0.75863	-2.17132	-0.20008
C	2.14368	-2.15609	-0.12783
C	2.81369	-0.95028	0.03578
C	2.17513	0.26971	0.13734
H	-4.30173	-1.75594	-1.05761
H	-3.25815	-0.63098	-1.95040
H	-3.93090	-0.14715	-0.39513
H	-1.93668	-2.40349	-0.84868
H	-3.08080	-1.28972	1.74948
H	-1.80452	-2.51244	1.64254
H	-3.43710	-2.88813	1.06846
H	1.07454	2.70750	0.79952
H	-1.89592	3.31198	0.51343
H	-0.65547	4.42878	1.12885
H	-1.05765	2.94808	2.02107
H	-0.59541	3.18006	-1.70775
H	1.10679	2.69122	-1.69504
H	0.64074	4.28215	-1.07240
H	0.22729	-3.10661	-0.32439
H	2.71997	-3.06962	-0.19666
H	2.74746	1.17965	0.25979
F	4.15420	-0.97355	0.09767

R = I

C	-4.71614	-1.55862	-0.93704
C	-3.43262	-1.92787	-0.22254
C	-3.69007	-2.48736	1.16772
N	-2.52947	-0.78449	-0.14355
C	-2.94068	0.49697	0.02153
N	-1.78652	1.20026	0.13959
C	-1.72132	2.65429	0.24534
C	-2.90767	3.19755	1.01444
C	-1.60141	3.27393	-1.13807
C	-0.66659	0.39515	0.04485
C	-1.15214	-0.90439	-0.12362
C	-0.28843	-1.98221	-0.23379
C	1.07416	-1.72790	-0.18468
C	1.54556	-0.42417	-0.02700
C	0.69290	0.66319	0.08980
H	-5.34930	-2.44375	-1.02697
H	-4.51026	-1.17085	-1.93591
H	-5.25255	-0.78834	-0.38340
H	-2.90812	-2.68255	-0.81822
H	-4.21590	-1.74496	1.77223
H	-2.75509	-2.74097	1.67194
H	-4.30490	-3.38805	1.11047
H	-0.81046	2.87252	0.81303
H	-3.83670	2.97811	0.48882
H	-2.80361	4.27895	1.12464
H	-2.97030	2.74760	2.00643
H	-2.50574	3.06677	-1.71463
H	-0.74626	2.86715	-1.68205
H	-1.47701	4.35620	-1.06308
H	-0.65069	-2.99591	-0.35272
H	1.77703	-2.54620	-0.26914
H	1.07719	1.66758	0.20776
I	3.60908	-0.09915	0.03353

R = Br

C	2.35147	3.43311	-0.89745
C	1.19459	2.78953	-0.16151
C	1.05457	3.31488	1.25863
N	1.32572	1.33618	-0.14791

C	2.51078	0.67870	-0.09362
N	2.16198	-0.63111	-0.01780
C	3.17425	-1.67802	0.04301
C	3.11732	-2.58157	-1.17773
C	3.10708	-2.44487	1.35350
C	0.24651	0.47646	-0.08904
C	0.78767	-0.81174	-0.01230
C	-0.04424	-1.92101	0.04820
C	-1.41462	-1.71320	0.03675
C	-1.93170	-0.42169	-0.03467
C	-1.12197	0.69877	-0.09818
H	2.19869	4.51361	-0.93738
H	2.42849	3.05099	-1.91645
H	3.29246	3.22204	-0.39002
H	0.27204	3.00235	-0.71214
H	0.88033	4.39284	1.25208
H	1.97084	3.11408	1.81834
H	0.22224	2.83604	1.77883
H	4.11008	-1.11679	0.01647
H	3.95114	-3.28605	-1.15603
H	3.18874	-1.99222	-2.09338
H	2.19296	-3.16109	-1.21702
H	3.94231	-3.14526	1.41535
H	2.18357	-3.01950	1.44683
H	3.16901	-1.76014	2.20097
H	0.34431	-2.92845	0.10481
H	-2.09103	-2.55617	0.08499
Br	-3.80895	-0.19171	-0.04118
H	-1.54718	1.69204	-0.14831

R = CHO

C	-3.41750	-1.78596	-1.04300
C	-2.17598	-2.04932	-0.21532
C	-2.50959	-2.54968	1.18068
N	-1.34180	-0.85351	-0.13200
C	-1.84287	0.40286	0.01861
N	-0.74279	1.18043	0.14366
C	-0.77659	2.63673	0.23947
C	-1.99856	3.10311	1.00317
C	-0.69654	3.25275	-1.14829
C	0.43112	0.45122	0.06891
C	0.03442	-0.88132	-0.09477
C	0.96676	-1.91060	-0.18442
C	2.30163	-1.56759	-0.12052
C	2.70305	-0.23266	0.03151
C	4.13064	0.10610	0.09295
O	5.03714	-0.68545	0.02420
C	1.76794	0.79508	0.12777
H	-3.99900	-2.70594	-1.13087
H	-3.15400	-1.44154	-2.04440
H	-4.03111	-1.01745	-0.57334
H	-1.56888	-2.80163	-0.72841
H	-3.10917	-1.80304	1.70600
H	-1.60319	-2.73422	1.76135
H	-3.07939	-3.47965	1.12774
H	0.11659	2.91937	0.80676
H	-2.90941	2.81613	0.47828
H	-1.96959	4.18991	1.10466
H	-2.03142	2.65809	1.99881
H	-1.58407	2.98128	-1.72415
H	0.18459	2.90030	-1.68877
H	-0.64528	4.34139	-1.08105
H	0.66243	-2.94365	-0.29783
H	3.07484	-2.32345	-0.18647
H	2.09698	1.82235	0.24302
H	4.33648	1.19260	0.21301

R = CO_2Me

C	-4.33359	-1.23868	-0.95746
C	-3.12164	-1.71543	-0.18327
C	-3.48022	-2.17539	1.22067
N	-2.09793	-0.67677	-0.12379
C	-2.37334	0.64659	0.02751
N	-1.15409	1.22479	0.12911
C	-0.93475	2.66484	0.21645
C	-2.05406	3.34070	0.98083
C	-0.75443	3.25092	-1.17503
C	-0.12281	0.30490	0.03904
C	-0.74472	-0.93968	-0.10958
C	-0.00114	-2.10833	-0.20870
C	1.37532	-1.99155	-0.16914
C	1.99992	-0.74586	-0.03210
C	3.47862	-0.72466	-0.00400
O	3.97264	0.51685	0.12504
C	5.39135	0.59943	0.15848
O	4.18042	-1.70082	-0.08632
C	1.25282	0.42517	0.07395
H	-5.06198	-2.04905	-1.02937
H	-4.05558	-0.92683	-1.96555
H	-4.79316	-0.38642	-0.45733
H	-2.66827	-2.55062	-0.72669
H	-3.93008	-1.34898	1.77539
H	-2.59484	-2.51288	1.76377
H	-4.19550	-2.99967	1.18277
H	-0.00348	2.79151	0.77867
H	-3.00310	3.21432	0.46034
H	-1.83560	4.40635	1.07641
H	-2.16054	2.91282	1.97883

H	-1.67768	3.13452	-1.74708
H	0.05064	2.74830	-1.71524
H	-0.51494	4.31458	-1.11398
H	-0.47259	-3.07772	-0.31089
H	2.00711	-2.86744	-0.24386
H	5.82174	0.20438	-0.76284
H	5.79009	0.03305	1.00129
H	5.62557	1.65640	0.26531
H	1.74784	1.38104	0.17724

R = COOH

C	-3.89218	-1.46967	-0.99232
C	-2.66041	-1.86432	-0.20334
C	-3.00403	-2.34819	1.19623
N	-1.71001	-0.75855	-0.13136
C	-2.07717	0.54276	0.02072
N	-0.90179	1.20305	0.13476
C	-0.78275	2.65481	0.22842
C	-1.95316	3.24910	0.98390
C	-0.63004	3.25657	-1.15959
C	0.19073	0.35646	0.05196
C	-0.34289	-0.92785	-0.10539
C	0.47946	-2.04330	-0.20032
C	1.84391	-1.83348	-0.14789
C	2.37972	-0.54774	-0.00220
C	3.84904	-0.42186	0.03986
O	4.26813	0.85218	0.17698
O	4.62561	-1.33975	-0.03746
C	1.55428	0.57024	0.09973
H	-4.56324	-2.32727	-1.07312
H	-3.62364	-1.13903	-1.99699
H	-4.41427	-0.65089	-0.49767
H	-2.14502	-2.66643	-0.74122
H	-3.51483	-1.55418	1.74543
H	-2.10469	-2.62619	1.75000
H	-3.66205	-3.21842	1.14971
H	0.13216	2.84311	0.80010
H	-2.88619	3.06076	0.45331
H	-1.80881	4.32668	1.08564
H	-2.04001	2.81067	1.97921
H	-1.53749	3.07864	-1.74102
H	0.21316	2.81313	-1.69339
H	-0.46536	4.33397	-1.09300
H	0.07577	-3.04205	-0.30905
H	2.53474	-2.66393	-0.21878
H	1.98179	1.55755	0.20983
H	5.23363	0.81176	0.19248

R = CN

C	-3.59287	-1.34966	-1.00766
C	-2.37871	-1.79341	-0.21768
C	-2.74318	-2.27329	1.17784
N	-1.38938	-0.72196	-0.13586
C	-1.70982	0.59008	0.01915
N	-0.51134	1.20887	0.14204
C	-0.34324	2.65642	0.24247
C	-1.48965	3.28422	1.00749
C	-0.17780	3.25938	-1.14331
C	0.54849	0.32528	0.06143
C	-0.02921	-0.93988	-0.10376
C	0.75167	-2.08289	-0.19936
C	2.12411	-1.92851	-0.14033
C	2.70256	-0.65940	0.01344
C	4.12204	-0.54153	0.06582
C	1.91829	0.49060	0.11636
H	-4.29288	-2.18279	-1.09704
H	-3.30971	-1.02119	-2.00897
H	-4.08830	-0.51727	-0.50842
H	-1.89050	-2.60955	-0.75989
H	-3.22803	-1.46559	1.73049
H	-1.85648	-2.58635	1.73334
H	-3.43106	-3.11948	1.12347
H	0.57969	2.81095	0.81133
H	-2.43084	3.13105	0.48008
H	-1.30866	4.35555	1.11503
H	-1.58609	2.84320	2.00075
H	-1.09356	3.11456	-1.72076
H	0.64766	2.79213	-1.68458
H	0.02239	4.33028	-1.07190
H	0.31313	-3.06594	-0.31417
H	2.77226	-2.79229	-0.21267
H	2.38328	1.46080	0.23211
N	5.26951	-0.44442	0.10816

R = NO_2

C	-3.82175	-1.53618	-1.01956
C	-2.60047	-1.90133	-0.20060
C	-2.96325	-2.36708	1.19996
N	-1.66761	-0.77922	-0.12823
C	-2.05970	0.51608	0.02091
N	-0.89769	1.20004	0.13452
C	-0.80737	2.65556	0.22666
C	-1.98314	3.22427	0.99315
C	-0.68007	3.25831	-1.16312
C	0.20870	0.37512	0.05452
C	-0.29959	-0.92200	-0.10095

C	0.54358	-2.02202	-0.19259
C	1.90579	-1.79294	-0.13993
C	2.38849	-0.49412	0.00310
N	3.83344	-0.28685	0.05092
O	4.54771	-1.26405	-0.03009
C	1.56645	0.61744	0.10350
H	-4.47906	-2.40423	-1.09992
H	-3.53843	-1.21775	-2.02412
H	-4.36499	-0.71788	-0.54741
H	-2.06288	-2.70335	-0.71608
H	-3.49379	-1.57117	1.72709
H	-2.07123	-2.62570	1.77463
H	-3.60969	-3.24583	1.15478
H	0.10850	2.86210	0.79012
H	-2.91680	3.01815	0.47035
H	-1.85966	4.30428	1.09468
H	-2.05185	2.78398	1.98905
H	-1.58821	3.05996	-1.73671
H	0.16883	2.83492	-1.70414
H	-0.53902	4.33893	-1.09799
H	0.15959	-3.02830	-0.29950
H	2.61520	-2.60541	-0.20784
H	1.99865	1.60212	0.21136
O	4.23501	0.85288	0.16895

S11 References

(1) Alvaro Fernandez Galiana; Simon Vilms Pedersen; Molly M. Stevens. B-Raman: An Open-Source, Versatile Platform for Automated Brightfield and Raman Microspectroscopy; 2025; Vol. PC13327, p PC133270C. https://doi.org/10.1117/12.3043951.

(2) Georgiev, D.; Fernández-Galiana, Á.; Vilms Pedersen, S.; Papadopoulos, G.; Xie, R.; Stevens, M. M.; Barahona, M. Hyperspectral Unmixing for Raman Spectroscopy via Physics-Constrained Autoencoders. *Proc. Natl. Acad. Sci.* **2024**, *121* (45), e2407439121. https://doi.org/10.1073/pnas.2407439121.

(3) Whitaker, D. A.; Hayes, K. A Simple Algorithm for Despiking Raman Spectra. *Chemom. Intell. Lab. Syst.* **2018**, *179*, 82–84. https://doi.org/10.1016/j.chemolab.2018.06.009.

(4) Eilers, P. H. C. A Perfect Smoother. *Anal. Chem.* **2003**, *75* (14), 3631–3636. https://doi.org/10.1021/ac034173t.

(5) Ye, J.; Tian, Z.; Wei, H.; Li, Y. Baseline Correction Method Based on Improved Asymmetrically Reweighted Penalized Least Squares for the Raman Spectrum. *Appl. Opt.* **2020**, *59* (34), 10933–10943. https://doi.org/10.1364/AO.404863.

(6) Georgiev, D.; Pedersen, S. V.; Xie, R.; Fernández-Galiana, Á.; Stevens, M. M.; Barahona, M. RamanSPy: An Open-Source Python Package for Integrative Raman Spectroscopy Data Analysis. *Anal. Chem.* **2024**, *96* (21), 8492–8500. https://doi.org/10.1021/acs.analchem.4c00383.

(7) *Erb, D. (2022). pybaselines: A Python library of algorithms for the baseline correction of experimental data (1.0.0). Zenodo. https://doi.org/10.5281/zenodo.7255880.*

(8) Albino, M.; Georgiev, D.; Silva, I.; Fernandez Debets, T. F. F.; Liu, M.; Dell, T. N.; Wojciechowski, J. P.; Stevens, M. M. Sieving for Gold─An Efficient Method for Generating *N* -Heterocyclic Carbene Self-Assembled Monolayers on Nanostructured Gold Surfaces. *J. Am. Chem. Soc.* **2025**, *147* (48), 44418–44429. https://doi.org/10.1021/jacs.5c15471.

(9) DeJesus, J. F.; Trujillo, M. J.; Camden, J. P.; Jenkins, D. M. *N* -Heterocyclic Carbenes as a Robust Platform for Surface-Enhanced Raman Spectroscopy. *J. Am. Chem. Soc.* **2018**, *140* (4), 1247–1250. https://doi.org/10.1021/jacs.7b12779.

(10) DeJesus, J. F.; Sherman, L. M.; Yohannan, D. J.; Becca, J. C.; Strausser, S. L.; Karger, L. F. P.; Jensen, L.; Jenkins, D. M.; Camden, J. P. A Benchtop Method for Appending Protic Functional Groups to N-Heterocyclic Carbene Protected Gold Nanoparticles. *Angew. Chem. Int. Ed.* **2020**, *59* (19), 7585–7590. https://doi.org/10.1002/anie.202001440.

(11) Chowdhury, S.; Hu, G.; Jensen, I. M.; Santos, A. V. B.; Jenkins, D. M.; Jensen, L.; Camden, J. P. Vibrational Mode Assignment of Diisopropyl Benzimidazolium *N* -Heterocyclic Carbenes on Gold. *J. Phys. Chem. C* **2024**, *128* (32), 13550–13557. https://doi.org/10.1021/acs.jpcc.4c02228.

(12) Gaussian 16, Revision C.01, M. J. Frisch, G. W. Trucks, H. B. Schlegel, G. E. Scuseria, M. A. Robb, J. R. Cheeseman, G. Scalmani, V. Barone, G. A. Petersson, H. Nakatsuji, X. Li, M. Caricato, A. V. Marenich, J. Bloino, B. G. Janesko, R. Gomperts, B. Mennucci, H. P. Hratchian, J. V. Ortiz, A. F. Izmaylov, J. L. Sonnenberg, D. Williams-Young, F. Ding, F. Lipparini, F. Egidi, J. Goings, B. Peng, A. Petrone, T. Henderson, D. Ranasinghe, V. G. Zakrzewski, J. Gao, N. Rega, G. Zheng, W. Liang, M. Hada, M. Ehara, K. Toyota, R. Fukuda, J. Hasegawa, M. Ishida, T. Nakajima, Y. Honda, O. Kitao, H. Nakai, T. Vreven, K. Throssell, J. A. Montgomery, Jr., J. E. Peralta, F. Ogliaro, M. J. Bearpark, J. J. Heyd, E. N. Brothers, K. N. Kudin, V. N. Staroverov, T. A. Keith, R. Kobayashi, J. Normand, K. Raghavachari, A. P. Rendell, J. C. Burant, S. S. Iyengar, J. Tomasi, M. Cossi, J. M. Millam, M. Klene, C. Adamo,

R. Cammi, J. W. Ochterski, R. L. Martin, K. Morokuma, O. Farkas, J. B. Foresman, and D. J. Fox, Gaussian, Inc., Wallingford CT, 2016.

(13) Becke, A. D. Density-Functional Exchange-Energy Approximation with Correct Asymptotic Behavior. *Phys. Rev. A* **1988**, *38* (6), 3098–3100. https://doi.org/10.1103/PhysRevA.38.3098.

(14) Perdew, J. P. Density-Functional Approximation for the Correlation Energy of the Inhomogeneous Electron Gas. *Phys. Rev. B* **1986**, *33* (12), 8822–8824. https://doi.org/10.1103/PhysRevB.33.8822.

(15) Weigend, F. Accurate Coulomb-Fitting Basis Sets for H to Rn. *Phys. Chem. Chem. Phys.* **2006**, *8* (9), 1057. https://doi.org/10.1039/b515623h.

(16) Weigend, F.; Ahlrichs, R. Balanced Basis Sets of Split Valence, Triple Zeta Valence and Quadruple Zeta Valence Quality for H to Rn: Design and Assessment of Accuracy. *Phys. Chem. Chem. Phys.* **2005**, *7* (18), 3297. https://doi.org/10.1039/b508541a.

(17) Grimme, S.; Ehrlich, S.; Goerigk, L. Effect of the Damping Function in Dispersion Corrected Density Functional Theory. *J. Comput. Chem.* **2011**, *32* (7), 1456–1465. https://doi.org/10.1002/jcc.21759.

(18) C. Adamo and V. Barone, "Toward Reliable Density Functional Methods without Adjustable Parameters: The PBE0 Model," J. Chem. Phys., 110 (1999) 6158-69. DOI: 10.1063/1.478522.

(19) Hanwell, M. D.; Curtis, D. E.; Lonie, D. C.; Vandermeersch, T.; Zurek, E.; Hutchison, G. R. Avogadro: An Advanced Semantic Chemical Editor, Visualization, and Analysis Platform. *J. Cheminformatics* **2012**, *4* (1), 17. https://doi.org/10.1186/1758-2946-4-17.

(20) Neese, F. Software Update: The ORCA Program System—Version 6.0. *WIREs Comput. Mol. Sci.* **2025**, *15* (2), e70019. https://doi.org/10.1002/wcms.70019.

(21) Neese, F. The ORCA Program System. *WIREs Comput. Mol. Sci.* **2012**, *2* (1), 73–78. https://doi.org/10.1002/wcms.81.

(22) Pantazis, D. A.; Chen, X.-Y.; Landis, C. R.; Neese, F. All-Electron Scalar Relativistic Basis Sets for Third-Row Transition Metal Atoms. *J. Chem. Theory Comput.* **2008**, *4* (6), 908–919. https://doi.org/10.1021/ct800047t.

(23) Neese, F. An Improvement of the Resolution of the Identity Approximation for the Formation of the Coulomb Matrix. *J. Comput. Chem.* **2003**, *24* (14), 1740–1747. https://doi.org/10.1002/jcc.10318.

(24) Neese, F.; Wennmohs, F.; Hansen, A.; Becker, U. Efficient, Approximate and Parallel Hartree–Fock and Hybrid DFT Calculations. A 'Chain-of-Spheres' Algorithm for the Hartree–Fock Exchange. *Chem. Phys.* **2009**, *356* (1–3), 98–109. https://doi.org/10.1016/j.chemphys.2008.10.036.

(25) Helmich-Paris, B.; De Souza, B.; Neese, F.; Izsák, R. An Improved Chain of Spheres for Exchange Algorithm. *J. Chem. Phys.* **2021**, *155* (10), 104109. https://doi.org/10.1063/5.0058766.

(26) Meng, G.; Kakalis, L.; Nolan, S. P.; Szostak, M. A Simple 1H NMR Method for Determining the σ-Donor Properties of N-Heterocyclic Carbenes. *Tetrahedron Lett.* **2019**, *60* (4), 378–381. https://doi.org/10.1016/j.tetlet.2018.12.059.
